# The effects of pro-, pre-, and synbiotics supplementation on polycystic ovary syndrome: an umbrella review of meta-analyses of randomized controlled trials

**DOI:** 10.3389/fnut.2023.1178842

**Published:** 2023-09-27

**Authors:** Sepide Talebi, Sheida Zeraattalab-Motlagh, Yahya Jalilpiran, Nastaran Payandeh, Shakila Ansari, Hamed Mohammadi, Kurosh Djafarian, Mahsa Ranjbar, Sara Sadeghi, Mahdiyeh Taghizadeh, Sakineh Shab-Bidar

**Affiliations:** ^1^Department of Clinical Nutrition, School of Nutritional Sciences and Dietetics, Tehran University of Medical Sciences, Tehran, Iran; ^2^Students’ Scientific Research Center (SSRC), Tehran University of Medical Sciences, Tehran, Iran; ^3^Department of Community Nutrition, School of Nutritional Sciences and Dietetics, Tehran University of Medical Sciences, Tehran, Iran; ^4^Department of Community Nutrition, School of Nutrition and Food Sciences, Food Security Research Center, Isfahan University of Medical Sciences, Isfahan, Iran

**Keywords:** synbiotics, meta-analysis, probiotics, prebiotics, polycystic ovary syndrome

## Abstract

**Background:**

Synbiotics, refer to a combination of probiotics and prebiotics in a form of synergism that beneficially affect the host’s health by alternating the composition and/or function of the gut microbiota. Numerous meta-analyses of randomized clinical trials have proven that pro, pre-, and synbiotics supplementation has health outcomes in women with polycystic ovary syndrome (PCOS). However, the strength and quality of this evidence in aggregate have not yet been synthesized in great detail.

**Methods:**

PubMed, Scopus, Web of Sciences, and Google Scholar were searched up to March 2023. We pooled the mean difference and its 95% confidence interval (CI) by applying a random-effects model.

**Results:**

Overall, nine meta-analyses including a total of 12 trials were identified. The results of the present study indicated that probiotic supplementation significantly reduced the homeostatic model assessment for insulin resistance (HOMA-IR; WMD: −0.29, 95% CI: −0.57 to −0.02, *p* = 0.03, *n* = 4; moderate certainty) and fasting glucose concentration (FGC; WMD: −7.5 mg/dL, 95% CI: −13.60 to −0.51, *p* = 0.03; *n* = 4; low certainty). Moreover, synbiotic supplementation had beneficial effects on glycemic control, lipid profile, and hormonal parameters, but the certainty of the evidence was rated as low to very low. However, supplementation with pro−/synbiotics did not affect inflammation and oxidative stress in women with PCOS. Furthermore, waist/hip circumference, fasting glucose concentration, lipid profile, dehydroepiandrosterone sulfate, high-sensitivity C-reactive protein, and hirsutism score were significantly reduced after prebiotics supplementation with low certainty of evidence.

**Conclusion:**

Although pro-, pre-, and synbiotics supplementation had beneficial effects on some PCOS-related outcomes, the certainty of the evidence was rated as low to very low. Therefore, further well-designed RCTs might help to confirm our findings in women with PCOS.

## Introduction

Polycystic ovary syndrome (PCOS) is a common endocrinopathy that affects women of reproductive age, particularly in the early to late reproductive stages (15–35 years) (1, 2). As defined in 2003 by the Rotterdam Consensus Declaration, the onset of two out of these following features is a sign of PCOS: oligo or anovulation, hyperandrogenism, and polycystic ovaries (3, 4). Depending on diagnostic criteria it is estimated that between 5 and 21% of women worldwide are affected by PCOS (5). Major complications of PCOS include insulin resistance (IR), glucose intolerance, type 2 diabetes mellitus, dyslipidemia, cardiovascular disease (6), hirsutism (7), acne, alopecia (8), and high C-reactive protein (9). The financial burden of PCOS, including the costs of initial diagnosis and reproductive endocrine complications, was estimated at $ 3.7 million per year in the United States and taking into account the cost of pregnancy-related and long-term complications, it has risen to $8 million per year (10).

Multiple pathophysiological mechanisms are assumed due to the heterogeneity of the PCOS characteristics. Hyperinsulinemia and insulin resistance, exaggerated LH pulse frequency and amplitude, and enhanced ovarian or adrenal androgen production, are the main presumed causes of PCOS (11, 12).

Recent studies regarding probiotics, “live microorganisms which when administered in adequate amounts confer a health benefit on the host,” demonstrated that the administration of probiotics can decrease intestinal permeability, modify the immune system of the gastrointestinal tract and prevent the growth of pathogenic bacteria (13–15). The term prebiotic is used as “a substrate that is selectively utilized by host microorganisms conferring a health benefit” (16). Short-chain fatty acids (SCFAs) from the metabolism of prebiotics, decrease inflammatory markers and subsequently reduce insulin resistance (17). The presence of a combination of living microorganisms and substrate(s) that host microorganisms use to their advantage and which benefits the host’s health is called synbiotics (18). Synbiotics administration was associated with significant improvement in fasting plasma glucose (FPG), homeostatic model assessment for insulin resistance (HOMA-IR) and body mass index (BMI) (19).

A substantial number of systematic reviews and meta-analyses (SRMAs) of randomized controlled trials on the effects of pro-, pre-, and synbiotics supplementation on PCOS-related outcomes (6, 20–22) have been conducted in recent years. Regardless of the high number of SRMAs, there is still some uncertainty about the efficacy of each prebiotic, probiotics, and synbiotics supplement separately. There is also currently no available data to support the certainty of the evidence for each estimate and the amount of impact detected based on the minimal clinically important differences (MCID). Also, the strength and quality of this evidence in aggregate have not yet been synthesized in great detail. Therefore, this umbrella review aims to examine systematic reviews to determine the effectiveness of pro-, pre-, and synbiotics on hormonal parameters, glycemic control markers, blood lipids, anthropometric indices, and inflammatory and oxidative stress biomarkers in women with PCOS and update the evidence.

## Methods

The current umbrella review was designed based on the protocols of the Cochrane Handbook for Systematic Reviews of Interventions on overviews of systematic reviews (23). The protocol of this umbrella review was registered in the International Prospective Register of Systematic Reviews (PROSPERO) database (https://www.crd.york.ac.uk/PROSPERO, CRD42021281029).

### Search strategy

The systematic search was conducted in major databases including PubMed, Web of Science, Scopus, and Google Scholar until 22 March 2023, with no restrictions on publication time or language. Detailed information relating to the search strategy of databases as well as the medical subject headings (MeSH) and text words in our search strategy to identify relevant studies are provided in Supplementary Table 1. We also added other literature that was found by manually reviewing related published SRMAs of RCTs evaluating the effects of pro-, pre-, and synbiotics supplementation in women with PCOS. Moreover, the references list of any related meta-analyses was manually reviewed to collect further eligible studies.

### Eligibility and study selection

Relevant studies were selected based on the PICOS (population/intervention/comparison/outcome) framework: P (women with polycystic ovary syndrome), I (pro-, pre- and synbiotics supplementation), C (placebo), O (PCOS-related outcomes), and study design (SRMAs of RCTs). Two authors (ST and NP) independently selected meta-analyses in this umbrella review if they met the following criteria: (1) SRMAs of RCTs that were conducted in the people of any age with a diagnosis of polycystic ovary syndrome; (2) received at least one oral probiotic, prebiotic, or synbiotics supplementation compared to a control group; (3) reported weighted or standardized mean differences (MDs) along with 95% confidence intervals (CIs); (4) reported at least one potential outcomes in published SRMAs of RCTs including hormonal parameters [dehydroepiandrosterone (DHEA), total testosterone (TT), and sex hormone-binding globulin (SHBG)], hirsutism score, fasting glucose concentration (FGC levels), markers for insulin (fasting insulin levels, HOMA-IR, and QUICKI), blood lipids [total cholesterol (TC), high-density lipoprotein cholesterol (HDL-C), low-density lipoprotein cholesterol (LDL-C), very low-density lipoprotein cholesterol (VLDL-C), and triglyceride (TG) levels], anthropometric indices (body weight, BMI, and waist circumference), inflammatory- and oxidative stress biomarkers [total antioxidant capacity (TAC), glutathione (GSH), malondialdehyde (MDA), nitric oxide (NO), and high-sensitivity c-reactive protein (hs-CRP)]. We excluded studies with insufficient data and other study designs. We also excluded primary trials in the meta-analysis if they: (1) were trials without a control group; (2) used pro-, pre-, and synbiotics supplementation in combination with other nutrients. If more than one published meta-analysis for a given outcome was available, we selected only the publication with the higher number of primary trials (24). Also, we have manually reviewed the reference lists of other meta-analyses to identify additional relevant trials.

### Data extraction

NP extracted the following data from eligible meta-analyses using a pre-designed abstraction form: first author’s name, country, publication year, number of primary studies, and participant number. Furthermore, for each primary RCTs from included meta-analyses, we also extracted the following required data: First author, country, publication year, effect size, participant number, duration of intervention, and the dose of supplementation.

### Assessment of methodological quality

A Measurement Tool to Assess Systematic Reviews (AMSTAR-2) scale (25) was used to evaluate the methodological quality of included meta-analysis by two independent researchers (ST and SZM). Disagreements were resolved by consensus with the third researcher (SSH). We also carried out the quality of primary trials including each eligible meta-analysis using the Cochrane risk-of-bias tool for randomized trials (RoB) (26). According to this systematic bias assessment, the overall quality of primary studies was scored as good, fair, or weak (Supplementary Table 2).

The AMSTAR 2 tool (25) was applied to assess the quality of conduct of the included meta-analyses of randomized controlled trials. Instrument (AMSTAR 2) retains 10 of the original domains, and has 16 items in total.

### Data synthesis and statistical analysis

For each health outcome, the largest meta-analysis with a maximum number of RCTs was selected, as well as primary trials that were ignored in the biggest meta-analyses were also added (Table 1). Then, we recalculated the MD and its 95% CI by applying a random-effects model in each meta-analysis that was included in our umbrella review (27). To evaluate the possibility of publication bias, we used Egger’s test method (28). Heterogeneity across studies was estimated by Cochran Q and *I*^2^ statistics, in which *I*^2^ values greater than 50% or *p* < 0.05 were considered as significant (29). Statistical analyses were conducted using STATA version 14 software (Stata Corp, College Station, Texas, United States).

**Table 1:** General characteristics of the published meta-analyses investigating the effects of pro-pre/synbiotic supplementation in patients with polycystic ovary syndrome.

Author, year	No. of primary trials	Number of primary trials included from other meta-analyses	Types of supplementation	Outcome	Sample	Dose (range, mg)	Follow-up (range, weeks)	ES	Effect size (95%CI)	*p* value	I^2^ (%)	*p*_heterogeneity_
					**Size**							
(6)	12	0	Probiotics	Body weight	731	≥2 × 10^8^ CFU	8–24 weeks	SMD	−0.02 (−0.36,0.31)	0.892	66.20%	0.007
			Synbiotic			<2 × 10^8^ CFU			−0.12 (−0.49,0.25)	0.534	53.50%	0.009
			Prebiotics						−0.61 (−1.12,−0.10)	0.019	NA	NA
(6)	13	1	Probiotics	BMI	791	≥2 × 10^8^ CFU	8–24 weeks	SMD	−0.03 (−0.24,0.19)	0.823	31.80%	0.174
			Prebiotics			<2 × 10^8^ CFU			−0.13 (−0.53,0.26)	0.508	58.90%	0.063
			Synbiotic						−0.66 (−1.17,-0.15)	0.012	NA	NA
(6)	5	0	Probiotics	WC	316	≥2 × 10^8^ CFU	8–24 weeks	SMD	Overall	Overall	Overall	Overall
			Prebiotics			<2 × 10^8^ CFU			0.37 (−0.78,1.53)	0.052	95.50%	0
			Synbiotic									
(6)	4	-	Probiotics	HC	256	≥2 × 10^8^ CFU	8–24 weeks	SMD	Overall	Overall	Overall	Overall
			Prebiotics			<2 × 10^8^ CFU			−0.25 (−0.78,0.27)	0.34	76.9	0.005
			Synbiotic									
(19)	3	0	Probiotics	Ferriman–Gallway score	855	≥2 × 10^9^ CFU	8–12 weeks	SMD	0.15 (−0.21,−0.51)	0.07	0	0.41
			Prebiotics			<2 × 10^9^ CFU			−0.56 (−1.07,−0.06)		-	-
			Synbiotic						−0.23 (−0.74,0.28)		-	-
(6)	8	0	Probiotics	FGC	496	≥2 × 10^8^ CFU	8–24 weeks	SMD	−0.96 (−1.86,−0.07)	0	90.50%	0.03
			Prebiotics			<2 × 10^8^C			−6.98 (−8.32,−5.63)	-	NA	0
			Synbiotic						−0.36 (−0.87,0.15)	0.04	67.70%	0.16
(6)	7	0	Probiotics	HOMA-IR	434	≥2 × 10^8^ CFU	8–24 weeks	SMD	−0.74 (−1.25,−0.23)	0.005	73.10%	0.011
			Synbiotic			<2 × 10^8^ CFU			−0.74 (−1.59,0.11)	0.08	87.20%	0
(6)	6	0	Probiotics	Insulin-sensitivity check index	379	≥2 × 10^8^ CFU	8–24 weeks	SMD	3.65 (0.71,6.58)	0.015	98.10%	0
			Synbiotic			<2 × 10^8^ CFU			0.92 (−0.12,1.96)	0.084	91.00%	0
(6)	7	0	Probiotics	FINS	434	≥2 × 10^8^ CFU	8–24 weeks	SMD	−0.70 (−1.13,-0.26)	0.002	63.60%	0.041
			Synbiotic			<2 × 10^8^ CFU			−0.67 (−1.54,0.20)	0.13	87.90%	0
(6)	7	1	Probiotics	TG	428 + 118	≥2 × 10^8^ CFU	8–12 weeks	SMD	−0.50 (−0.80,−0.20)	0.001	0.00%	0.92
			Prebiotics			<2 × 10^8^ CFU			−4.41 (−5.35,−3.48)	0	NA	NA
			Synbiotic						−0.14 (−0.47,0.20)	0.42	25.20%	0.26
(6)	7	1	Probiotics	TC	428 + 118	≥2 × 10^8^ CFU	8–12 weeks	SMD	−0.26 (−0.85,0.32)	0.4	0.00%	0.43
			Prebiotics			<2 × 10^8^ CFU			−7.52 (−8.95,−6.08)	0	NA	NA
			Synbiotic						−0.28 (−0.56,0.01)	0.12	0.00%	0.5
(6)	7	1	Probiotics	HDL-c	428 + 118	≥2 × 10^8^ CFU	8–12 weeks	SMD	−0.17 (−0.98,0.63)	0.67	85.90%	0.001
			Prebiotics			<2 × 10^8^ CFU			4.28 (3.37,5.20)	0	NA	NA
			Synbiotic						0.09 (−0.48,0.65)	0.76	72.70%	0.026
(6)	7	1	Probiotics	LDL-c	428 + 118	≥2 × 10^8^ CFU	8–12 weeks	SMD	−0.13 (−0.42,0.17)	0.4	0.00%	0.43
			Prebiotics			<2 × 10^8^ CFU			−5.57 (−6.69,-4.46)	0	NA	NA
			Synbiotic						−0.22 (−0.51,0.06)	0.12	0.00%	0.5
(6)	4	1	Probiotics	VLDL-c	235 + 118	≥2 × 10^8^ CFU	8–12 weeks	SMD	−0.48 (−0.78,−0.18)	0.002	0.00%	0.95
			Synbiotic			<2 × 10^8^ CFU			−0.32 (−0.83,0.19)	0.21	NA	NA
(6)	9	1	Probiotics	CRP	558 + 118	≥2 × 10^8^ CFU	8–12 weeks	SMD	Overall	Overall	Overall	Overall
			Synbiotic			<2 × 10^8^ CFU			−0.63 (−1.37,0.10)	0.089	93.90%	0
			Prebiotics									
(22)	4	0	Probiotics	NO	240	≥2 × 10^8^ CFU	8–12 weeks	SMD	Overall	Overall	Overall	Overall
			Synbiotic			<2 × 10^8^ CFU			0.33 (0.08, 0.59)	0.01	0.00%	0.39
(22)	4	1	Probiotics	TAC	240 + 86	≥2 × 10^8^ CFU	8–12 weeks	SMD	Overall	Overall	Overall	Overall
			Synbiotic			<2 × 10^8^ CFU			0.64 (0.38,0.90)	<0.001	0.00%	0.58
(22)	4	0	Probiotics	GSH	240	≥2 × 10^8^ CFU	8–12 weeks	SMD	Overall	Overall	Overall	Overall
			Synbiotic			<2 × 10^8^ CFU			0.26 (0.01,0.52)	0.04	0.00%	0.57
(22)	4	1	Probiotics	MDA	240 + 86	≥2 × 10^8^ CFU	8–12 weeks	SMD	Overall	Overall	Overall	Overall
			Synbiotic			<2 × 10^8^ CFU			−0.90 (−1.16,−0.63)	<0.001	0.00%	0.63
(22)	6	1	Probiotics	TT	326	≥2 × 10^8^ CFU	8–12 weeks	SMD	Overall	Overall	Overall	Overall
			Synbiotic			<2 × 10^8^ CFU			−0.58 (−0.82,−0.34)	<0.001	10.40%	0.34
(58)	3	1	Probiotics	DHEAS	182 + 62	≥2 × 10^8^ CFU	8–12 weeks	SMD	0.00 (−0.51,0.51)	1	Overall	Overall
			Synbiotic			<2 × 10^8^ CFU			−0.31 (−0.82,0.20)	0.24	0.00%	0.57
			Prebiotics						−0.36 (−0.86,0.14)	0.16		
(22)	4	0	Probiotics	SHBG	240	≥2 × 10^8^ CFU	8–12 weeks	SMD	Overall	Overall	Overall	Overall
			Synbiotic			<2 × 10^8^ CFU			0.46 (0.08,0.85)	0.01	55.70%	0.08

Overall: synbiotic, prebiotic, probiotic supplementation. BMI, Body mass index; CI, Confidence interval; DHEAS, Dehydroepiandrosterone sulfate; FGC, Fasting glucose concentration; FI, Fasting insulin; GSH; Glutathione; HOMA-IR, Homeostasis model assessment-estimated insulin resistance; HDL, High density lipoprotein; hs-CRP, High sensitive c-reactive protein; LDL, Low density lipoprotein; MDA, Malondialdehyde; NO, Nitric oxide; QUICKI, Quantitative insulin sensitivity check index; SHBG, Sex hormone binding globulin; TC, Total cholesterol; TG, Triglycerides; TAC, Total antioxidant capacity; TT, Total testosterone; VLDL, Very low density lipoprotein; WC, Waist circumference; and wk, Week.

### Grading of the evidence

The certainty of the evidence was rated according to the Grading of Recommendations Assessment, Development and Evaluations (GRADE) (30). The GRADE consists of five domains: risk of bias in the individual studies, inconsistency, indirectness, imprecision, and publication bias. As a result, high, medium, low, or very low-GRADE ratings were considered for the certainty of evidence. The MCID for the estimations was determined using previous data in the literature, and in the absence of sufficient evidence, we used half of the baseline SDs for that outcome (31). Supplementary Table 3 demonstrates the MCID values utilized in the current umbrella review.

## Results

### Literature search

**Figure 1:**
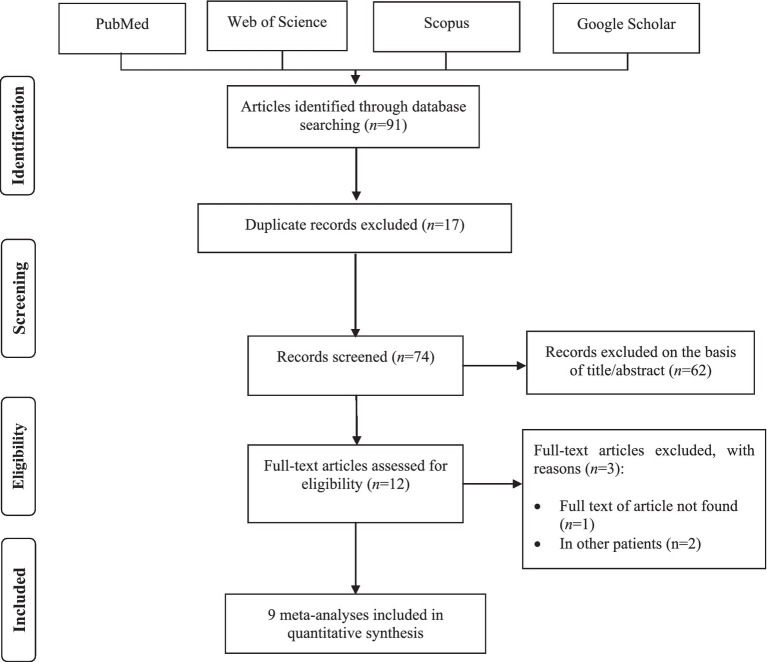
Literature search and review flow diagram for selection of umbrella review meta-analyses.

We identified a total of 91 meta-analyses studies through initial electronic searches. After removing 17 duplicated studies, 62 publications were assessed based on reviewing titles and abstracts. Of those, 12 records remained for full-text revision. Among them, three articles were excluded due to the full text being unavailable (32) and performed on other patients (33, 34). Overall, nine meta-analyses were finally included in this umbrella review. The flow diagram of the study selection process is illustrated in Figure 1. Through the screening primary studies of included meta-analyses, five RCTs were excluded for either of the following reasons: full text being unavailable (*n* = 2) (35, 36) and using probiotics in combination with other interventions (*n* = 3) (37–39). Detailed reasons for the exclusion of primary trials by full-text assessing are provided in Supplementary Table 4. Overall, nine meta-analyses (6, 20–22, 40–44) reporting 12 RCTs (45–55) met the eligibility criteria for the final analysis in this umbrella review.

### Study characteristics (Description of original RCTs)

Of the 12 primary trials included in this review, four studies with six arms used synbiotics (21, 46, 52), two trials used prebiotics (53, 54), and the remaining used probiotics (45, 47–49, 51, 55). Seven trials were double-blind (45, 47–50, 52, 55) and four trials were triple-blind placebo-controlled trials (46, 53, 54), while one trial was a single-blinded clinical trial (51). Included trials were published between 2017 and 2021. All primary studies were conducted in Iran (45–50, 52–55) and Egypt (51). The follow-up duration among primary studies varied between 8 and 12 weeks and the dosage of probiotic or synbiotic supplementation ranged from 2 × 10^8^ to 3 × 10^10^ CFU/day. Characteristics of eligible primary studies are illustrated in Table 2.

**Table 2:** Characteristics of eligible primary studies on the effects of pro-pre/synbiotic supplementation in patients with polycystic ovary syndrome.

First author (Country; year)	RCT design (Blinding)	Supplementation	Strains	Mean age (year)	Mean BMI (kg/m^2^)	Sample size (Supplementation/Placebo)	Duration (weeks)	Intervention		Outcomes
								Treatment group	Control group	
(59)	Parallel (Double)	Probiotic	*Lactobacillus acidophilus*, *Lactobacillus casei*, and *Bifidobacterium bifidum*	25	25	60 (30/30)	12	2 × 10^9^ CFU	Placebo (ND)	FGC, TC, LDL, HDL, TG, Insulin, HOMA-IR, QUICKI, Weight, and BMI
(60)	Parallel (Double)	Synbiotic	*Lactobacillus casei*, *Lactobacillus ramnosousa, Lactobacillus plantroum*, and *Bacillus koagolans*, indicousa	30	26	56 (23/23)	8	2 × 10^8^ CFU	Placebo (water + pomegranate flavoring)	FGC, Insulin, HOMA-IR, QUICKI, Weight, BMI, Testosterone, LH, and FSH
(61)	Parallel (Double)	Probiotic	*Lactobacillus acidophilus, Lactobacillus plantarum, Lactobacillus fermentum,* and *Lactobacillus gasseri*	30	26	60 (30/30)	12	2 × 10^9^ CFU	Placebo (ND)	Weight, BMI, HsCRP, and WC
(62)	Parallel (Double)	Probiotic	*Lactobacillus acidophilus, Lactobacillus casei*, *and Bifidobacterium bifidum*	27	23	60 (30/30)	12	2 × 10^9^ CFU	Placebo (starch)	Testosterone, SHBG, DHEA, HsCRP, NO, TAC, GSH, MDA, NO, and mF-G
(63)	Parallel (Double)	Synbiotic	*Lactobacillus acidophilus* 3 × 10^10^ CFU/g, *Lactobacillus casei* 3 × 10^9^ CFU/g, *Lactobacillus bulgaricus* 5 × 10^8^ CFU/g, *Lactobacillus rhamnosus* 7 × 10^9^ CFU/g, *Bifidobacterium longum* 1 × 10^9^ CFU/g, *Bifidobacterium breve* 2 × 10^10^ CFU/g, *Streptococcus thermophilus* 3 × 10^8^ CFU/g, and prebiotic Inulin (fructooligosaccharide)	28	32	99 (50/49)	12	500 mg	Placebo (starch)	FGC, Cho, LDL, HDL, TG, BP, Weight, BMI, and WHR
(64)	Parallel (Double)	Synbiotic	*Lactobacillus acidophilus* 2 × 10^9^ CFU/g, *Lactobacillus casei* 2 × 10^9^ CFU/g, and *Bifidobacterium bifidum* 2 × 10^9^ CFU/g plus 0.8 g inulin	25	27	60 (30/30)	12	2 × 10^9^ CFU	Placebo (ND)	Testosterone, SHBG, DHEA, HsCRP, NO, TAC, GSH, MDA, NO, mF-G, Weight, and BMI
(65)	Parallel (Double)	Probiotic	Lactobacillus delbruekii, *Lactobacillus fermentum*	30	34	60 (30/30)	12	1 × 10^9^ CFU	ND	FGC, Cho, LDL, HDL, TG, Insulin, HOMA-IR, HsCRP, Weight, and BMI
(66)	Parallel (Double)	Synbiotic	*Lactobacillus acidophilus* 2 × 10^9^ CFU/g, *Lactobacillus casei* 2 × 10^9^ CFU/g, *Bifidobacterium bifidum* 2 × 10^9^ CFU/g plus 0.8 g inulin	27	27	60 (30/30)	12	2 × 10^9^ CFU	Placebo (starch)	FGC, TC, LDL, HDL, TG, Insulin, HOMA-IR, QUICKI, Weight, and BMI
(67)	Parallel (Double)	Prebiotic	20 g of resistant Dextrin	31	25	62 (31/31)	12	20 g	Placebo (Maltodextrin)	Weight, BMI, and WC
(56)	Parallel (Double)	Probiotic	*Lactobacillus casei* 7 × 10^9^ CFU/g, *Lactobacillus acidophilus* 2 × 10^9^ CFU/g, *Lactobacillus rhamnosus* 1.5 × 10^9^ CFU/g, *Lactobacillus bulgaricus* 2 × 10^8^ CFU/g, *Bifidobacterium breve* 2 × 10^10^ CFU/g, *Bifidobacterium longum* 7 × 10^9^ CFU/g, and *Streptococcus thermophiles* 1.5 × 10^9^ CFU/g	25	25	72 (36/35)	8	500 mg	Placebo (Maltodextrin)	FGC, Insulin, HOMA-IR, and CRP
(68)	Parallel (Double)	Prebiotic	20 g of resistant Dextrin	31	25	62 (31/31)	12	20 g	Placebo (Maltodextrin)	TC, LDL, HDL, TG, HsCRP, Testosterone, and DHEA
(47)	Parallel (Double)	Synbiotic	*Lactobacillus casei*, *Lactobacillus ramnosousa,* and *Lactobacillus plantroum*, and *Bacillus koagolans*, indicousa	30	26	56 (23/23)	8	2 × 10^8^ CFU	Placebo (water + pomegranate flavoring)	TC, LDL, HDL, TG, HsCRP, TAC, MDA, and BP

BMI, Body mass index; FGC, Fasting glucose concentration; HOMA-IR, Homeostatic model assessment of insulin resistance; QUICKI, Quantitative insulin sensitivity check index; LH, Luteinizing hormone; FSH, Follicle stimulating hormone; BP, Blood pressure; MDA, Malondialdehyde; TAC, Total antioxidant capacity; Hs-CRP, High sensitive C-reactive protein; TG, Triglyceride; TC, Total cholesterol; LDL, Low-density lipoprotein; HDL, High-density lipoprotein; GSH, Glutathione; NO, nitric oxide; DHEA, Dehydroepiandrosterone; and SHBG, Sex hormone binding globulin.

### Methodological quality

According to AMSTAR 2 scores, two meta-analyses were classified as high-quality studies (6, 42), four meta-analyses were performed with a low-quality method (21, 22, 40, 44), and the other three meta-analyses were performed with a critically low-quality method (20, 41, 43). Detailed AMSTAR scores for each meta-analysis are presented in Supplementary Table 5.

### Findings from the meta-analysis

#### Probiotic supplementation in patients with PCOS

Six primary trials from nine systematic reviews and meta-analyses evaluated the impact of probiotic supplementation in patients with PCOS. We found moderate-certainty evidence that probiotic supplementation significantly reduced HOMA-IR compared to the control group (WMD: −0.29, 95% CI: −0.57 to −0.02, *p* = 0.03) with no significant between-study heterogeneity (*I*^2^ = 33.8%, *p* = 0.20). There was also low certainty of evidence that probiotic supplementation had a significant effect on FGC (WMD: −7.5 mg/dL, 95% CI: −13.60 to −0.51, *p* = 0.03), VLDL-C (WMD: −50.40 mg/dL, 95% CI: −9.91 to −0.89, *p* = 0.01), WC (WMD: 0.86 cm, 95% CI: 0.38–1.33, *p* < 0.001), TT (WMD: −0.40 ng/mL, 95% CI: −0.73 to −0.07, *p* = 0.017), SHBG level (WMD: 25.40 nmol/L, 95% CI: 12.50–38.30, *p* < 0.001), TAC (WMD: 107.10 mmol/L, 95% CI: 8.95–1.61, *p* < 0.001), MDA (WMD: 1.10 μmol/L, 95% CI: 0.59–1.61, *p* < 0.001), and hirsutism score (WMD: -1.50, 95% CI: −2.50 to −0.85, *p* < 0.001). However, supplementation with probiotics had no significant effects on other outcomes (Table 3). The results of GRADE are described in Supplementary Table 6. We could not perform subgroup analyses due to the small number of primary studies.

**Table 3:** The effects of probiotic supplementation in women with PCOS.

Outcomes (unit)	Number of trials (arms)	Number of participants	Follow-up (range), wk	Dose (range), CFU	Effect size (95% CI)	*p* value	I^2^ (%)	*p*_heterogeneity_	Egger’s test	Certainty of evidence (GRADE)^1^
Body weight (kg)	4	309	12	1–3 × 10^9^	0.25 (−1.37, 1.88)	0.759	97.1	<0.001	0.500	Low
BMI (kg/m^2^)	5	409	12	1 × 10^9^–2 × 10^12^	0.44 (−0.23, 1.12)	0.199	94.3	<0.001	0.264	Low
Waist circumference (cm)	2	189	12	1 × 10^9^–3 × 10^10^	0.86 (0.38, 1.33)	<0.001	0.0	0.496	-	Low
Hip circumference (cm)	1	99	12	3 × 10^10^	−0.60 (−1.09, 2.29)	0.487	-	-	-	Low
Fasting glucose concentration (mg/dL)	4	331	8–12	2 × 10^9^–3 × 10^10^	−7.05 (−13.60, −0.51)	0.035	93.7	<0.001	0.781	Low
Fasting insulin	4	331	8–12	2 × 10^9^–3 × 10^10^	−0.40 (−0.94, 0.15)	0.152	82.6	0.01	0.098	Low
HOMA-IR	4	331	8–12	2 × 10^9^–3 × 10^10^	−0.29 (−0.57, −0.02)	0.037	33.8	0.209	0.536	Moderate
QUICKI	3	231	8–12	2–7 × 10^9^	0.01 (−0.0, 0.01)	0.240	62.0	0.072	0.627	Low
Triglycerides (mg/dL)	3	259	12	2 × 10^9^–2 × 10^12^	−39.51 (−95.42, 16.40)	0.166	97.5	<0.001	0.224	Low
Very low-density lipoprotein (mg/dL)	1	60	12	2 × 10^9^	−50.40 (−9.91, −0.89)	0.019	-	-	-	Low
Total cholesterol (mg/dL)	3	259	12	2 × 10^9^–2 × 10^12^	−4.29 (−19.62, 11.04)	0.584	89.4	<0.001	0.428	Low
HDL cholesterol (mg/dL)	3	259	12	2 × 10^9^–2 × 10^12^	6.20 (−5.38, 17.77)	0.294	53.5	0.117	0.013	Low
LDL cholesterol (mg/dL)	3	259	12	2 × 10^9^–2 × 10^12^	−3.80 (−8.93, 1.32)	0.146	99.0	<0.001	0.874	Low
Total testosterone (ng/mL)	1	60	12	2 × 10^9^	−0.40 (−0.73, −0.07)	0.017	-	-	-	Low
Dehydroepiandrosterone sulfate (μg/mL)	1	60	12	2 × 10^9^	0.17 (−0.01, 0.35)	0.063	-	-	-	Low
Sex hormone-binding globulin (nmol/L)	1	60	12	2 × 10^9^	25.40 (12.50, 38.30)	<0.001	-	-	-	Low
C-reactive protein (mg/L)	2	171	8–12	7 × 10^9^–3 × 10^10^	0.92 (−0.57, 2.40)	0.226	73.3	0.053	-	Very low
high-sensitivity C-reactive protein (mg/L)	3	250	12	1 × 10^9^–2 × 10^12^	0.50 (−1.92, 2.93)	0.684	99.1	<0.001	0.307	Low
Nitric oxide (μmol/L)	1	60	12	2 × 10^9^	1.80 (−1.49, 5.09)	0.284	-	-	-	Low
Total antioxidant capacity (mmol/L)	1	60	12	2 × 10^9^	107.10 (8.95, 205.25)	0.032	-	-	-	Low
Glutathione (GSH; μmol/L)	1	60	12	2 × 10^9^	70.80 (−5.39, 146.99)	0.069	-	-	-	Low
Malondialdehyde (μmol/L)	1	60	12	2 × 10^9^	1.10 (0.59, 1.61)	<0.001	-	-	-	Low
Hirsutism score	1	60	12	2 × 10^9^	−1.50 (−2.15, −0.85)	<0.001	-	-	-	Low

1 GRADE, Grading of recommendations assessment, development, and evaluation.

BMI, Body mass index; CFU, Colony-forming unit; CI, confidence interval; and wk, week.

#### Synbiotics supplementation in patients with PCOS

Overall, four primary clinical trials with six arms from nine meta-analyses were included in the analyses to evaluate the effects of synbiotics supplementation in women with PCOS. There was low certainty of evidence that synbiotic supplementation had a significant reduction in WC (WMD: −2.70 cm, 95% CI: −4.28 to −1.12, *p* = 0.001), fasting insulin (SMD: −0.90, 95% CI: −1.24 to −0.57, *p* < 0.001), HOMA-IR (WMD: −0.82, 95% CI: −1.09 to −0.56, *p* < 0.001), VLDL-C (WMD: −4.40 mg/dL, 95% CI: −7.19 to −1.61, *p* = 0.002), TC (WMD: −10.57 mg/dL, 95% CI: −20.83 to −0.31, *p* = 0.04), LDL-C (WMD: −21.58 mg/dL, 95% CI: −41.62 to −1.53, *p* = 0.03), TT (WMD: −0.13 ng/mL, 95% CI: −0.18 to −0.09, *p* < 0.001), and hirsutism score (WMD: −1.20, 95% CI: −2.11 to −0.29, *p* = 0.01). We also observed that synbiotics supplementation significantly increased SHBG (WMD: 19.30 nmol/L, 95% CI: 2.26–36.34, *p* = 0.02) compared to the placebo with low certainty of evidence. Moreover, pooled analysis suggested the significant effect of synbiotics consumption on QUICKI (WMD: 0.01, 95% CI: 0.00–0.01, *p* = 0.03), and TG (WMD: −15.37 mg/dL, 95% CI: −22.53 to −8.21, *p* = 0.001), but the certainty of the evidence was rated as very low. Intake of synbiotics supplementation had no significant effect on other outcomes in women with PCOS (Table 4). Detailed GRADE scores for each outcome are shown in Supplementary Table 7.

**Table 4:** The effects of synbiotic supplementation in women with PCOS.

Outcomes (unit)	Number of trials (arms)	Number of participants	Follow-up (range), wk	Dose (range), mg/d	Effect size (95% CI)	*p* value	*I*^2^ (%)	*p*_heterogeneity_	Egger’s test	Certainty of evidence (GRADE)^1^
Body weight (kg)	3 (4)	304	8–12	2 × 10^9^−2 × 10^8^	−0.19 (−0.79, 0.42)	0.546	40.2	0.170	0.131	Moderate
BMI (kg/m^2^)	3 (4)	304	8–12	2 × 10^9^−2 × 10^8^	−0.06 (−0.33, 0.21)	0.668	56.8	0.074	0.070	Low
Waist circumference (cm)	1 (2)	184	8	2 × 10^8^	−2.70 (−4.28, −1.12)	0.001	0.0	0.747	-	Low
Hip circumference (cm)	1 (2)	184	8	2 × 10^8^	−0.03 (−1.75, 1.69)	0.970	0.0	0.964	-	Low
Fasting glucose concentration (mg/dL)	2 (3)	244	8–12	2 × 10^8^–2 × 10^9^	−1.94 (−3.95, 0.08)	0.060	0.0	0.722	0.040	Low
Fasting insulin	2 (3)	244	8–12	2 × 10^8^–2 × 10^9^	−0.90 (−1.24, −0.57)	<0.001	0.0	0.479	0.644	Low
HOMA-IR	2 (3)	244	8–12	2 × 10^8^–2 × 10^9^	−0.82 (−1.09, −0.56)	<0.001	0.0	0.438	0.717	Low
QUICKI	2 (3)	244	8–12	2 × 10^8^–2 × 10^9^	0.01 (0.0, 0.01)	0.037	85.6	0.001	0.647	Very low
Triglycerides (mg/dL)	2 (3)	232	8–12	2 × 10^8^–2 × 10^9^	−15.37 (−22.53, −8.21)	0.001	0.0	0.554	0.742	Very low
Very low density lipoprotein (mg/dL)	1	60	12	2 × 10^9^	−4.40 (−7.19, −1.61)	0.002	-	-	-	Low
Total cholesterol (mg/dL)	2 (3)	232	8–12	2 × 10^8^–2 × 10^9^	−10.57 (−20.83, −0.31)	0.043	35.3	0.217	0.245	Low
HDL cholesterol (mg/dL)	2 (3)	232	8–12	2 × 10^8^–2 × 10^9^	3.02 (−2.57, 8.62)	0.289	80.4	0.006	0.937	Very low
LDL cholesterol (mg/dL)	2 (3)	232	8–12	2 × 10^8^–2 × 10^9^	−21.58 (−41.62, −1.53)	0.035	47.0	0.151	0.424	Low
Total testosterone (ng/mL)	2 (3)	244	8–12	2 × 10^8^–2 × 10^9^	−0.13 (−0.18, −0.09)	<0.001	22.7	0.274	0.245	Low
Dehydroepiandrosterone sulfate (μg/mL)	1	60	12	2 × 10^9^	−0.30 (−0.72, 0.12)	0.160	-	-	-	Low
Sex hormone-binding globulin (nmol/L)	1	60	12	2 × 10^9^	19.30 (2.26, 36.34)	0.026	-	-	-	Low
high-sensitivity C-reactive protein (mg/L)	2 (3)	232	8–12	2 × 10^8^–2 × 10^9^	−0.15 (−0.39, 0.09)	0.216	90.0	<0.001	0.626	Very low
Nitric oxide (μmol/L)	1	60	12	2 × 10^9^	5.20 (1.52, 8.88)	0.006	-	-	-	Low
Total antioxidant capacity (mmol/L)	2 (3)	232	8–12	2 × 10^9^−3 × 10^10^	−0.10 (−0.42, 0.23)	0.566	62.5	0.070	0.306	Very low
Glutathione (GSH; μmol/L)	1	60	12	2 × 10^9^	−2.60 (−49.70, 44.50)	0.914	-	-	-	Low
Malondialdehyde (μmol/L)	2 (3)	232	8–12	2 × 10^9^−3 × 10^10^	−0.27 (−0.45, 0.09)	0.003	40.4	0.187	0.258	Low
Hirsutism score	1	60	12	2 × 10^9^	−1.20 (−2.11, −0.29)	0.010	-	-	-	Low

1 GRADE, Grading of recommendations assessment, development, and evaluation.

BMI, Body mass index; CFU, Colony-forming unit; CI, confidence interval; and wk, Week.

#### Prebiotic supplementation in patients with PCOS

The effect of prebiotic supplementation in women with PCOS was examined in two primary studies from two meta-analyses. There was low certainty of evidence that supplementation with prebiotics significantly reduced WC (WMD: −5.10 cm, 95% CI: −8.60 to −1.60, *p* = 0.004), hip circumference (HC; WMD: −4.60 cm, 95% CI: −7.47 to −1.73, *p* = 0.002), FGC (WMD: −15.14 mg/dL, 95% CI: −20.38 to −9.90, *p* = 0.003), TG (WMD: −31.12 mg/dL, 95% CI: −49.63 to −12.61, *p* = 0.06), TC (WMD: −34.83 mg/dL, 95% CI: −52.47 to −17.19, *p* < 0.001) LDL-C (WMD: −37.65 mg/dL, 95% CI: −52.09 to −22.69, *p* < 0.001), DHEA-S (WMD: −0.84 μg/mL, 95% CI: −1.52 to −0.16, *p* = 0.01), hs-CRP (WMD: −1.94 mg/L, 95% CI: −3.27 to −0.61, *p* = 0.00), and hirsutism score (WMD: -1.68, 95% CI: −3.19 to −0.17, *p* = 0.02). However, prebiotic supplementation did not have a significant effect on other outcomes in women with PCOS (Table 5). Detailed GRADE evidence for prebiotic supplementation in patients with PCOS was presented in Supplementary Table 8.

**Table 5:** The effects of prebiotic supplementation in women with PCOS.

Outcomes (unit)	Number of trials (arms)	Number of participants	Follow-up (range), wk	Dose (range), mg/d	Effect size (95% CI)	*p* value	*I*^2^ (%)	*p*_heterogeneity_	Egger’s test	Certainty of evidence (GRADE)^1^
Body weight (kg)	1	62	24	20,000	−2.80 (−6.83, 1.23)	0.173	-	-	-	Low
BMI (kg/m^2^)	1	62	24	20,000	−1.40 (−2.95, 0.15)	0.077	-	-	-	Low
Waist circumference (cm)	1	62	24	20,000	−5.10 (−8.60, −1.60)	0.004	-	-	-	Low
Hip circumference (cm)	1	62	24	20,000	−4.60 (−7.47, −1.73)	0.002	-	-	-	Low
Fasting glucose concentration (mg/dL)	1	62	12	20,000	−15.14 (−20.38, −9.90)	0.003	-	-	-	Low
Triglycerides (mg/dL)	1	62	12	20,000	−31.12 (−49.63, −12.61)	0.064	-	-	-	Low
Total cholesterol (mg/dL)	1	62	12	20,000	−34.83 (−52.47, −17.19)	<0.001	-	-	-	Low
HDL cholesterol (mg/dL)	1	62	12	20,000	7.20 (−1.63, 11.40)	0.001	-	-	-	Low
LDL cholesterol (mg/dL)	1	62	12	20,000	−37.65 (−52.09, −22.69)	<0.001	-	-	-	Low
Dehydroepiandrosterone sulfate (μg/mL)	1	62	12	20,000	−0.84 (−1.52, −0.16)	0.016	-	-	-	Low
High-sensitivity C-reactive protein (mg/L)	1	62	12	20,000	−1.94 (−3.27, −0.61)	0.004	-	-	-	Low
Hirsutism score	1	62	12	20,000	−1.68 (−3.19, −0.17)	0.029	-	-	-	Low

1 GRADE, Grading of recommendations assessment, development, and evaluation.

BMI, Body mass index; CI, Confidence interval; and wk, week.

#### Publication bias

We found statistically significant publication bias regarding the levels of HDL-C (Egger’s = 0.01) following intake of probiotic supplementation, and the levels of FGC after supplementation with synbiotics (Egger’s = 0.04). Therefore, we did the trim-and-fill method to detect sources of bias and found results similar to the original. No evidence of publication bias based on Egger’s tests was observed in other outcomes (Tables 3–5).

## Discussion

The present work was performed on meta-analyses of RCTs to comprehensively assess the effects of pro-, pre-, and synbiotics supplementation on PCOS-related outcomes. We evaluated the evidence using the well-known GRADE tool and to provide better comparisons between outcomes, the available data were reanalyzed using random effects analysis. Our findings are important because there is limited evidence-based support for use of pro-, pre-, and synbiotics supplements in the management of PCOS-related outcomes.

The results of the present study showed probiotic supplementation significantly reduced HOMA-IR, FGC, and VLDL. In addition, synbiotics supplementation was found to have beneficial effects in the reduction of WC, fasting insulin, HOMA-IR, TG, VLDL, TC, LDL-c, TT, and hirsutism score. Moreover, we found prebiotic supplementation significantly reduced WC, HC, FGC, TG, TC, LDL-c, dehydroepiandrosterone sulfate, hs-CRP, and hirsutism score. In contrast, our study showed that probiotic supplementation significantly increased WC, SHBG, TAC, and MDA parameters. It was also found synbiotics supplementation significantly increased SHBG and QUICKI. These findings should, however, be interpreted with some caution due to the following reasons: Firstly, almost all of the significant findings in the analyses received low and very low-quality evidence based on the GRADE tool. Only moderate quality of evidence was found for the effects of probiotics on the HOMA-IR index. None of the included meta-analyses considered this critical point and their findings were judged based on statistical differences. The included meta-analyses in this umbrella review were also evaluated for methodological accuracy using the AMSTAR tool. According to this method, three meta-analyses showed critically low quality, three showed low quality, and two showed high quality. The meta-analyses were rated as low and critically low-quality methods because did not register the protocol of the meta-analysis, had no comprehensive search strategies, did not report the reasons for excluded studies, and did not discuss the possible risk of bias in primary studies. Secondly, most of the analyses were performed on limited number of studies (≤5) with less than 12 months of follow up duration. It is interesting that for some outcomes only one RCT was available, so the results seem unreliable. Thirdly, our results showed high evidence of statistical heterogeneity between the studies in some analyses which weakens the clinical certainty of the results (56, 57). Unfortunately, a low number of primary RCTs made it impossible to conduct subgroup analyses, so we were unable to find sources of heterogeneity between studies (*n* < 10). Fourthly, the effects of an intervention on selected outcomes are not solely based on statistical significance but should also be judged on clinical relevance. For example, the results of the current umbrella review showed inconsistent findings regarding the potential effects of pro-, pre-, and synbiotics supplementation on WC in patients with PCOS. Accordingly, probiotic supplementation slightly, but not clinically important, increased WC (0.86 cm) compared to the control group. In contrast, synbiotics and prebiotic supplementation decreased WC by nearly −2.7 and − 5.10 cm, respectively. Of course, these findings with low-quality evidence were obtained from data from only two trials for probiotics and one trial for synbiotics and prebiotics. Also, possible explanations for this inconsistency might be the short duration of the interventions. It is recommended that extend the treatment period for central obesity beyond 12 weeks (70, 71). Fifthly, it is imperative to consider strain-specific efficacy when using probiotics or symbiotics in the treatment or prevention of disease. The efficacy of potential probiotic strains varies according to experimental studies (72). As a result, it is important to determine whether the microbes can survive from ingestion to delivery to the target organ, whether the microbes are capable of interfering with pathogenesis (usually using animal models of disease), and whether they can be sustained from ingestion to administration (73). Interestingly, among 127 studied Lactobacillus strains, only 3% were found to be capable of being used as probiotics due to their ability to survive in the target organ and to withstand bile and stomach acidity (74). In addition, over 170 Lactobacillus species were examined in depth, revealing significant differences in resistance to antibiotics and probiotic potential (75). A probiotic strain’s presence or absence of the different factors could explain why some strains are effective in some types of diseases but are not effective in others. However, a direct comparison of different strains is relatively uncommon, and multiple trials for the same strain or mixture are not common for the same disease. Strain-specificity can be accounted for by including only probiotics belonging to the same strain in meta-analyses. Another strategy is conducting subgroup analyses with the same probiotic strains within each sub-group. The results of previous research showed that not all probiotic strains are as effective as originally believed based on subgroup analyses and re-analysis of the data (76–78). This critical point was not taken into account by any of the meta-analyses that included in this umbrella review. Our review on the primary included RCTs also showed that all of those studies intervened by mixture of probiotic strains. Among them, two trials intervened by symbiotic formulas with the same probiotic and prebiotic mixture (50, 52) and two by capsules with the same probiotic mixture (45, 48) while others contained different strains of probiotics. Accordingly, due to the lack of included primary studies, we were unable to perform subgroup analyses to cover this important note in detail.

The main mechanisms behind these beneficial effects of pro-, pre-, and synbiotics on PCOS-related outcomes are still unclear. However, one possible explanation may be due to the effects of these compounds on short-chain fatty acids (SCFAs), the main by-products of fermentation in the intestinal lumen. The production of SCFAs has been shown to influence intestinal mucosal integrity, resulting in reduced inflammation, microbial endotoxins, and insulin resistance. In addition, the SCFAs play a role in the regulation of food intake and blood glucose homeostasis through the regulation of the secretion of gut peptides such as peptide YY and glucagon-like peptide-1 (79). Moreover, it has been suggested that the SCFAs inhibit the activation of the rate-limiting enzyme in the cholesterol production pathway, hydroxymethylglutaryl-CoA reductase (HMG-CoA reductase), which leads to lower cholesterol metabolism and better lipid metabolism (80). Regarding sex hormones and hirsutism score, it has been found that probiotics or synbiotic supplements increase mucin formation, enhance bowel function, and reduce the quantity of gram-negative (inappropriate) bacteria in the colon. These modifications lessen the transmission of lipopolysaccharides (LPS) along the mucous wall and metabolic endotoxemia, which can ultimately result in improvements in insulin receptor function, lower levels of insulin, and increased levels of normal ovarian function, which in turn reduce the production of androgens such as DHEA, FAI, and testosterone (81, 82). As well, a limited number of RCTs with a short duration (less than 12 weeks) make it impossible to draw any conclusions regarding the impact of pro-pre- and synbiotic supplementation on PCOS-related outcomes, which adds to the importance of further studies in this area.

Our study had some strengths. This is the first study evaluating the effects of pro-, pre-, and synbiotic supplementation on several outcomes in patients with PCOS. To conduct this review, we selected the largest meta-analyses for each outcome, excluded RCTs without inclusion criteria, and recalculated effect sizes for each outcome, whenever possible. In addition, the certainty of the evidence was assessed using the GRADE tool. As a valid and acceptable tool, it helps the findings of systematic reviews to be more elucidative and informative. Accordingly, our review showed that, in most cases, the results of the meta-analyses were accompanied by small effect sizes and low or very low certainty of the evidence.

Our study has some limitations that should be considered. First, since the primary studies were limited to Iran and Egypt, these findings seem to have limited generalizability. Second, the number of studies for each outcome was limited and only one study has been conducted on the effects of prebiotics on PCOS-related outcomes. Third, the validity of our findings is impacted by considerable heterogeneity in some pooled results. Of course, we were unable to perform subgroup analyses to detect potential sources of heterogeneity because there were less than 10 trials available for each analysis. Forth, different probiotic and synbiotic supplementation across trials and the pooling of their effects added uncertainty to the interpretation of specific findings to each outcome. For example, although in the pooled data analysis probiotic supplementation improved FGC levels, synbiotic supplementation did not show any significant result. Fifth, the included meta-analyses did not obtain data from unpublished information, which may lead to publication bias. Sixth, it is impossible to fully control the confounding effects of other components of the diet via statistical methods, therefore, the effects of a pro-prebiotic and synbiotic supplementation may be partially mediated by other diet components. Seventh, the results of this study may be also cofounded by other PCOS-related lifestyle factors, such as body weight, age, and levels of physical activity. There were few primary studies, so we were unable to conduct subgroup analyses to take these factors into account.

## Conclusion

In conclusion, the results of the present umbrella review suggests the beneficial effects of probiotics and synbiotics supplementation on the HOMA-IR index. However, the results originated from pooled data of the low number of RCTs with a maximum duration of 12 weeks. Also, we could not find a conclusive finding for other outcomes because of some important limitations such as small sample sizes in primary trials, small pooled effect sizes, and low or very low certainty in the evidence. Therefore, further well-designed RCTs with the following criteria might help to confirm or reject our findings in patients with PCOS: studies with different races and larger sample sizes; comparing the effects of different types of pro-, pre-, and synbiotic supplements on specific outcomes, RCTs with longer periods and larger sample sizes to assess and compare the effects of different dose of supplements, reporting all potential side effects following probiotics supplementation, and comparing the effects of different probiotics, prebiotics, and synbiotics to the promotion of evidence about the effects of these different interventions.

Our review generated several key messages for clinicians and patients, notably those who are eager for an adjuvant approach to the treatment of PCOS. Even though there are a variety of pathways that support the advantages of pro/pre and synbiotic supplementation in women with PCOS, it is critical to highlight that the magnitude of the effect was not clinically important, and the certainty of the evidence was low and very low. It is critical to highlight that there is insufficient data to support their obvious and long-term clinical effects.

## Data availability statement

The original contributions presented in the study are included in the article/Supplementary material, further inquiries can be directed to the corresponding author.

## Author contributions

ST, SS-B, and KD performed data interpretation, design, search, and statistical analysis. NP collated the data. SZ-M, MR, SS, and MT arbitrated the study quality. ST, YJ, and SA contributed to writing the manuscript. HM and SS-B revised the draft manuscript. All authors contributed to the article and approved the submitted version.

## Abbreviations

PCOS: Polycystic ovary syndrome
GRADE: Grading of recommendations assessment development and evaluations
CI: Confidence interval
WMD: Weighted mean differences
RCTs: Randomized clinical trials
IR: Insulin resistance
SCFAs: Short-chain fatty acids
FPG: Fasting plasma glucose
HOMA-IR: Homeostatic model assessment for insulin resistance
BMI: Body mass index
SRMAs: Systematic reviews and meta-analyses
MCID: Minimal clinically important differences
TC: Total cholesterol
HDL-C: High-density lipoprotein cholesterol
LDL-C: Low-density lipoprotein cholesterol
VLDL-C: Very low-density lipoprotein cholesterol
TG: Triglyceride
WC: Waist circumference
TAC: Total antioxidant capacity
GSH: Glutathione
MDA: Malondialdehyde
NO: Nitric oxide
hs-CRP: High-sensitivity c-reactive protein

## References

[1] Azziz R Carmina E Chen Z Dunaif A Laven JSE Legro RS . Polycystic ovary syndrome. Nat Rev Dis Primers. (2016) 2:16057. doi: 10.1038/nrdp.2016.57 27510637

[2] Norman RJ Dewailly D Legro RS Hickey TE. Polycystic ovary syndrome. Lancet. (2007) 370:685–97. doi: 10.1016/S0140-6736(07)61345-2, PMID: 17720020

[3] Rotterdam ESHRE/ASRM-Sponsored PCOS Consensus Workshop Group. Revised 2003 consensus on diagnostic criteria and long-term health risks related to polycystic ovary syndrome. Fertil Steril. (2004) 81:19–25. doi: 10.1016/j.fertnstert.2003.10.004, PMID: 14711538

[4] Giampaolino P Della Corte L De Rosa N Mercorio A Bruzzese D Bifulco G. Ovarian volume and PCOS: A controversial issue. Gynecol Endocrinol. (2018) 34:229–32. doi: 10.1080/09513590.2017.1391205 29043882

[5] Brakta S Lizneva D Mykhalchenko K Imam A Walker W Diamond MP . Perspectives on polycystic ovary syndrome: is polycystic ovary syndrome research underfunded? J Clin Endocrinol Metab. (2017) 102:4421–7. doi: 10.1210/jc.2017-01415, PMID: 29092064

[6] Li Y Tan Y Xia G Shuai J. Effects of probiotics, prebiotics, and synbiotics on polycystic ovary syndrome: a systematic review and meta-analysis. Crit Rev Food Sci Nutr. (2021). 63:522–538. doi: 10.1080/10408398.2021.1951155 34287081

[7] Glintborg D Andersen M. An update on the pathogenesis, inflammation, and metabolism in hirsutism and polycystic ovary syndrome. Gynecol Endocrinol. (2010) 26:281–96. doi: 10.3109/09513590903247873, PMID: 20141388

[8] Quinn M Shinkai K Pasch L Kuzmich L Cedars M Huddleston H. Prevalence of androgenic alopecia in patients with polycystic ovary syndrome and characterization of associated clinical and biochemical features. Fertil Steril. (2014) 101:1129–34. doi: 10.1016/j.fertnstert.2014.01.003, PMID: 24534277

[9] Boulman N Levy Y Leiba R Shachar S Linn R Zinder O . Increased C-reactive protein levels in the polycystic ovary syndrome: a marker of cardiovascular disease. J Clin Endocrinol Metab. (2004) 89:2160–5. doi: 10.1210/jc.2003-031096, PMID: 15126536

[10] Riestenberg C Jagasia A Markovic D Buyalos RP Azziz R. Health care-related economic burden of polycystic ovary syndrome in the united states: pregnancy-related and long-term health consequences. J Clin Endocrinol Metab. (2021) 107:575–85. doi: 10.1210/clinem/dgab613, PMID: 34546364

[11] Giampaolino P Foreste V Di Filippo C Gallo A Mercorio A Serafino P . Microbiome and PCOS: State-of-art and future aspects. Int J Mol Sci. (2021) 22:1–16. doi: 10.3390/ijms22042048 PMC7922491 33669557

[12] Tsilchorozidou T Overton C Conway GSJCE. The pathophysiology of polycystic ovary syndrome. Clin Endocrinol. (2004) 60:1–17. doi: 10.1046/j.1365-2265.2003.01842.x, PMID: 14678281

[13] Hill C Guarner F Reid G Gibson GR Merenstein DJ Pot B . Expert consensus document: The International Scientific Association for Probiotics and Prebiotics consensus statement on the scope and appropriate use of the term probiotic. Nat Rev Gastroenterol Hepatol. (2014) 11:506–14. doi: 10.1038/nrgastro.2014.66, PMID: 24912386

[14] Strowski MZ Wiedenmann B. Probiotic carbohydrates reduce intestinal permeability and inflammation in metabolic diseases. Gut. (2009) 58:1044–5. doi: 10.1136/gut.2009.179325 19592687

[15] Yurtdaş G Akdevelioğlu Y. A new approach to polycystic ovary syndrome: the gut microbiota. J Am Coll Nutr. (2020) 39:371–82. doi: 10.1080/07315724.2019.1657515, PMID: 31513473

[16] Gibson GR Hutkins RW Sanders ME Prescott SL Reimer RA Salminen SJ . The International Scientific Association for Probiotics and Prebiotics (ISAPP) consensus statement on the definition and scope of prebiotics. Nat Rev Gastroenterol Hepatol. (2017) 14:491–502. doi: 10.1038/nrgastro.2017.75 28611480

[17] Voltolini C Battersby S Etherington SL Petraglia F Norman JE Jabbour HNJE. A novel antiinflammatory role for the short-chain fatty acids in human labor. Endocrinology. (2012) 153:395–403. doi: 10.1210/en.2011-1457 22186417

[18] Swanson KS Gibson GR Hutkins R Reimer RA Reid G Verbeke K . The International Scientific Association for Probiotics and Prebiotics (ISAPP) consensus statement on the definition and scope of synbiotics. Nat Rev Gastroenterol Hepatol. (2020) 17:687–701. doi: 10.1038/s41575-020-0344-2, PMID: 32826966 PMC7581511

[19] Cozzolino M Vitagliano A Pellegrini L Chiurazzi M Andriasani A Ambrosini G . Therapy with probiotics and synbiotics for polycystic ovarian syndrome: a systematic review and meta-analysis. Eur J Nutr. (2020) 59:2841–56. doi: 10.1007/s00394-020-02233-0, PMID: 32372265

[20] Heshmati J Farsi F Yosaee S Razavi M Rezaeinejad M Karimie E . The effects of probiotics or synbiotics supplementation in women with polycystic ovarian syndrome: a systematic review and meta-analysis of randomized clinical trials. Probiot Antimicrob Proteins. (2019) 11:1236–47. doi: 10.1007/s12602-018-9493-9, PMID: 30547393

[21] Miao CY Guo QG Fang XJ Chen Y Zhao Y Zhang Q. Effects of probiotic and synbiotic supplementation on insulin resistance in women with polycystic ovary syndrome: a meta-analysis. J Int Med Res. (2021) 49:03000605211031758. doi: 10.1177/03000605211031758, PMID: 34311599 PMC8320576

[22] Tabrizi R Ostadmohammadi V Akbari M Lankarani KB Vakili S Peymani P . The effects of probiotic supplementation on clinical symptom, weight loss, glycemic control, lipid and hormonal profiles, biomarkers of inflammation, and oxidative stress in women with polycystic ovary syndrome: a systematic review and meta-analysis of randomized controlled trials. Probiot Antimicrob Proteins. (2019) 14:1–14. doi: 10.1007/s12602-019-09559-0, PMID: 31165401

[23] Higgins JP Altman DG Sterne JA. Assessing risk of bias in included studies. The Cochrane Collaboration In: Higgins JPT Green S, editors. Cochrane Handbook for Systematic Reviews of Interventions Version 5.1. 0 (2011). The Cochrane Collaboration; 2011:243–296. Available at: www.handbook.cochrane.org

[24] Neuenschwander M Ballon A Weber KS Norat T Aune D Schwingshackl L . Role of diet in type 2 diabetes incidence: umbrella review of meta-analyses of prospective observational studies. BMJ. (2019) 366:l2368. doi: 10.1136/bmj.l2368, PMID: 31270064 PMC6607211

[25] Shea BJ Reeves BC Wells G Thuku M Hamel C Moran J . AMSTAR 2: a critical appraisal tool for systematic reviews that include randomised or non-randomised studies of healthcare interventions, or both. BMJ. (2017) 358:j4008. doi: 10.1136/bmj.j4008, PMID: 28935701 PMC5833365

[26] Higgins JP Altman DG Gøtzsche PC Jüni P Moher D Oxman AD . The Cochrane Collaboration’s tool for assessing risk of bias in randomised trials. BMJ. (2011) 343:d5928. doi: 10.1136/bmj.d5928, PMID: 22008217 PMC3196245

[27] Dersimonian R Laird N. Meta-analysis in clinical trials. Control Clin Trials. (1986) 7:177–88. doi: 10.1016/0197-2456(86)90046-2 3802833

[28] Egger M Smith GD Schneider M Minder C. Bias in meta-analysis detected by a simple, graphical test. BMJ. (1997) 315:629–34. doi: 10.1136/bmj.315.7109.629, PMID: 9310563 PMC2127453

[29] Higgins JP Thompson SG Deeks JJ Altman DG. Measuring inconsistency in meta-analyses. BMJ. (2003) 327:557–60. doi: 10.1136/bmj.327.7414.557, PMID: 12958120 PMC192859

[30] Guyatt GH Oxman AD Vist GE Kunz R Falck-Ytter Y Alonso-Coello P . GRADE: an emerging consensus on rating quality of evidence and strength of recommendations. BMJ. (2008) 336:924–6. doi: 10.1136/bmj.39489.470347.AD, PMID: 18436948 PMC2335261

[31] Norman GR Sloan JA Wyrwich KW. Interpretation of changes in health-related quality of life: the remarkable universality of half a standard deviation. Med Care. (2003) 41:582–92. doi: 10.1097/01.MLR.0000062554.74615.4C, PMID: 12719681

[32] Liao D Zhong C Li C Mo L Liu Y. Meta-analysis of the effects of probiotic supplementation on glycemia, lipidic profiles, weight loss and C-reactive protein in women with polycystic ovarian syndrome. Minerva Med. (2018) 109:479–87. doi: 10.23736/S0026-4806.18.05728-2, PMID: 30256077

[33] López-Moreno A Aguilera M. Vaginal probiotics for reproductive health and related dysbiosis: systematic review and meta-analysis. J Clin Med. (2021) 10:1461. doi: 10.3390/jcm10071461, PMID: 33918150 PMC8037567

[34] Pourrajab B. Fatahi S. Sohouli M.H. Găman M.-A. Shidfar F.J.C.R.I.F.S., and Nutrition (2021). The effects of probiotic/synbiotic supplementation compared to placebo on biomarkers of oxidative stress in adults: a systematic review and meta-analysis of randomized controlled trials. Crit Rev Food Sci Nutr 62, 490–507. doi: 10.1080/10408398.2020.1821166, PMID: 33016089

[35] Ye C Ming-Hui Z Chan-Ni W. Effect of probiotic supplementation on blood sugar and lipids in patients with polycystic ovary syndrome. J Int Obstetr Gynecol. (2018) 45:199.

[36] Zhu X Xia P He Y. Effects of vitamin D and probiotics supplementation on bacterial diversity, metabolism and hormone level in patients with polycystic ovary syndrome. Chin J Microecol. (2020) 32:317–21.

[37] Jamilian M Mansury S Bahmani F Heidar Z Amirani E Asemi Z. The effects of probiotic and selenium co-supplementation on parameters of mental health, hormonal profiles, and biomarkers of inflammation and oxidative stress in women with polycystic ovary syndrome. J Ovar Res. (2018) 11:1–7. doi: 10.1186/s13048-018-0457-1 PMC6137747 30217229

[38] Ostadmohammadi V Jamilian M Bahmani F Asemi Z. Vitamin D and probiotic co-supplementation affects mental health, hormonal, inflammatory and oxidative stress parameters in women with polycystic ovary syndrome. J Ovarian Res. (2019) 12:5. doi: 10.1186/s13048-019-0480-x, PMID: 30665436 PMC6340184

[39] Shabani A Noshadian M Jamilian M Chamani M Mohammadi S Asemi Z. The effects of a novel combination of selenium and probiotic on weight loss, glycemic control and markers of cardio-metabolic risk in women with polycystic ovary syndrome. J Funct Foods. (2018) 46:329–34. doi: 10.1016/j.jff.2018.04.071

[40] Cozzolino M Vitagliano A. PROBIOTICS and synbiotics for polycystic ovarian syndrome: a systematic review and meta-analysis. Fertil Steril. (2019) 112:E391. doi: 10.1016/j.fertnstert.2019.07.1117 32372265

[41] Hadi A Moradi S Ghavami A Khalesi S Kafeshani M. Effect of probiotics and synbiotics on selected anthropometric and biochemical measures in women with polycystic ovary syndrome: a systematic review and meta-analysis. Eur J Clin Nutr. (2020) 74:543–7. doi: 10.1038/s41430-019-0434-9, PMID: 31053754

[42] Kazemi A Soltani S Ghorabi S Keshtkar A Daneshzad E Nasri F . Effect of probiotic and synbiotic supplementation on inflammatory markers in health and disease status: A systematic review and meta-analysis of clinical trials. Clin Nutr. (2020) 39:789–819. doi: 10.1016/j.clnu.2019.04.004, PMID: 31060892

[43] Shamasbi SG Ghanbari-Homayi S Mirghafourvand M. The effect of probiotics, prebiotics, and synbiotics on hormonal and inflammatory indices in women with polycystic ovary syndrome: a systematic review and meta-analysis. Eur J Nutr. (2020) 59:433–50. doi: 10.1007/s00394-019-02033-1, PMID: 31256251

[44] Zhang C Sheng Y Jiang J Xue Y Yu L Tian F . Probiotics supplementation for management of type II diabetes risk factors in adults with polycystic ovarian syndrome: a meta-analysis of randomized clinical trial. Food Sci Human Wellness. (2023) 12:1053–63. doi: 10.1016/j.fshw.2022.10.023

[45] Ahmadi S Jamilian M Karamali M Tajabadi-Ebrahimi M Jafari P Taghizadeh M . Probiotic supplementation and the effects on weight loss, glycaemia and lipid profiles in women with polycystic ovary syndrome: a randomized, double-blind, placebo-controlled trial. Hum Fertil. (2017) 20:254–61. doi: 10.1080/14647273.2017.1283446, PMID: 28142296

[46] Esmaeilinezhad Z Barati-Boldaji R Brett N De Zepetnek J Bellissimo N Babajafari S . The effect of synbiotics pomegranate juice on cardiovascular risk factors in PCOS patients: a randomized, triple-blinded, controlled trial. J Endocrinol Investig. (2020) 43:539–48. doi: 10.1007/s40618-019-01139-x, PMID: 31713129

[47] Esmaeilinezhad Z Barati-Boldaji R Brett NR De Zepetnek JO Bellissimo N Babajafari S . The effect of synbiotics pomegranate juice on cardiovascular risk factors in PCOS patients: a randomized, triple-blinded, controlled trial. Journal of endocrinological investigation. (2020) 43:539–48. 31713129 10.1007/s40618-019-01139-x

[48] Ghanei N Rezaei N Amiri GA Zayeri F Makki G Nasseri E. The probiotic supplementation reduced inflammation in polycystic ovary syndrome: a randomized, double-blind, placebo-controlled trial. J Funct Foods. (2018) 42:306–11. doi: 10.1016/j.jff.2017.12.047, PMID: 30217229

[49] Karamali M Eghbalpour S Rajabi S Jamilian M Bahmani F Tajabadi-Ebrahimi M . Effects of probiotic supplementation on hormonal profiles, biomarkers of inflammation and oxidative stress in women with polycystic ovary syndrome: a randomized, double-blind, placebo-controlled trial. Arch Iran Med. (2018) 21:1–7. 29664663

[50] Karimi E Heshmati J Shirzad N Vesali S Hosseinzadeh-Attar MJ Moini A . The effect of synbiotics supplementation on anthropometric indicators and lipid profiles in women with polycystic ovary syndrome: a randomized controlled trial. Lipids Health Dis. (2020) 19:1–9. doi: 10.1186/s12944-020-01244-4 32248805 PMC7132870

[51] Nasri K Jamilian M Rahmani E Bahmani F Tajabadi-Ebrahimi M Asemi Z. The effects of synbiotic supplementation on hormonal status, biomarkers of inflammation and oxidative stress in subjects with polycystic ovary syndrome: a randomized, double-blind, placebo-controlled trial. BMC Endocr Disord. (2018) 18:1–8. doi: 10.1186/s12902-018-0248-0 29649996 PMC5898079

[52] Rashad NM Amal S Amin AI Soliman MH. Effects of probiotics supplementation on macrophage migration inhibitory factor and clinical laboratory feature of polycystic ovary syndrome. J Funct Foods. (2017) 36:317–24. doi: 10.1016/j.jff.2017.06.029

[53] Samimi M Dadkhah A Haddad Kashani H Tajabadi-Ebrahimi M Seyed Hosseini E Asemi Z. The effects of synbiotic supplementation on metabolic status in women with polycystic ovary syndrome: a randomized double-blind clinical trial. Probiot Antimicrob Proteins. (2019) 11:1355–61. doi: 10.1007/s12602-018-9405-z, PMID: 29532416

[54] Shamasbi SG Dehgan P Charandabi SM-A Aliasgarzadeh A Mirghafourvand M. The effect of resistant dextrin as a prebiotic on metabolic parameters and androgen level in women with polycystic ovarian syndrome: a randomized, triple-blind, controlled, clinical trial. Eur J Nutr. (2019) 58:629–40. doi: 10.1007/s00394-018-1648-7, PMID: 29480399

[55] Shamasbi SG Dehghan P Charandabi SM-A Aliasgarzadeh A Mirghafourvand M. Effect of prebiotic on anthropometric indices in women with polycystic ovarian syndrome: a triple-blind, randomized, controlled clinical trial. Iran Red Crescent Med J. (2018) 20:201–8. doi: 10.1016/j.numecd.2018.07.002, PMID: 30538082

[56] Shoaei T Heidari-Beni M Tehrani HG. Effects of probiotic supplementation on pancreatic β-cell function and c-reactive protein in women with polycystic ovary syndrome: a randomized double-blind placebo-controlled clinical trial. Int J Prev Med. (2015) 6:27. doi: 10.4103/2008-7802.153866, PMID: 25949777 PMC4387688

[57] Aune D Norat T Romundstad P Vatten LJ. Whole grain and refined grain consumption and the risk of type 2 diabetes: a systematic review and dose–response meta-analysis of cohort studies. Eur J Epidemiol. (2013) 28:845–58. doi: 10.1007/s10654-013-9852-5, PMID: 24158434

[58] Shamasbi SG Ghanbari-Homayi S Mirghafourvand M. The effect of probiotics, prebiotics, and synbiotics on hormonal and inflammatory indices in women with polycystic ovary syndrome: a systematic review and meta-analysis. European journal of nutrition. (2020) 59:433–50. 31256251 10.1007/s00394-019-02033-1

[59] Ahmadi S Jamilian M Karamali M Tajabadi-Ebrahimi M Jafari P Taghizadeh M . Probiotic supplementation and the effects on weight loss, glycaemia and lipid profiles in women with polycystic ovary syndrome: a randomized, double-blind, placebo-controlled trial. Human Fertility. (2017) 20:254–61. 28142296 10.1080/14647273.2017.1283446

[60] Esmaeilinezhad Z Babajafari S Sohrabi Z Eskandari MH Amooee S Barati-Boldaji R. Effect of synbiotic pomegranate juice on glycemic, sex hormone profile and anthropometric indices in PCOS: A randomized, triple blind, controlled trial. Nutrition, Metabolism and Cardiovascular Diseases. (2019) 29:201–8. 10.1016/j.numecd.2018.07.002 30538082

[61] Ghanei N Rezaei N Amiri GA Zayeri F Makki G Nasseri E. The probiotic supplementation reduced inflammation in polycystic ovary syndrome: a randomized, double-blind, placebo-controlled trial. Journal of functional foods. (2018) 42:306–11.

[62] Karamali M Eghbalpour S Rajabi S Jamilian M Bahmani F Tajabadi-Ebrahimi M . Effects of probiotic supplementation on hormonal profiles, biomarkers of inflammation and oxidative stress in women with polycystic ovary syndrome: a randomized, double-blind, placebo-controlled trial. Archives of Iranian medicine. (2018) 21:1–7. 29664663

[63] Karimi E Heshmati J Shirzad N Vesali S Hosseinzadeh-Attar MJ Moini A . The effect of synbiotics supplementation on anthropometric indicators and lipid profiles in women with polycystic ovary syndrome: a randomized controlled trial. Lipids in health and disease. (2020) 19:1–9. 32248805 10.1186/s12944-020-01244-4 PMC7132870

[64] Nasri K Jamilian M Rahmani E Bahmani F Tajabadi-Ebrahimi M Asemi Z. The effects of synbiotic supplementation on hormonal status, biomarkers of inflammation and oxidative stress in subjects with polycystic ovary syndrome: a randomized, double-blind, placebo-controlled trial. BMC endocrine disorders. (2018) 18:1–8. 29649996 10.1186/s12902-018-0248-0 PMC5898079

[65] Rashad NM Amal S Amin AI Soliman MH. Effects of probiotics supplementation on macrophage migration inhibitory factor and clinical laboratory feature of polycystic ovary syndrome. Journal of functional foods. (2017) 36:317–24.

[66] Samimi M Dadkhah A Haddad Kashani H Tajabadi-Ebrahimi M Seyed Hosseini E Asemi Z. The effects of synbiotic supplementation on metabolic status in women with polycystic ovary syndrome: a randomized double-blind clinical trial. Probiotics and antimicrobial proteins. (2019) 11:1355–61. 29532416 10.1007/s12602-018-9405-z

[67] Shamasbi SG Dehghan P Charandabi SM Aliasgarzadeh A Mirghafourvand M. Effect of prebiotic on anthropometric indices in women with polycystic ovarian syndrome: a triple-blind, randomized, controlled clinical trial. Iran Red Crescent Med J. (2018) 20:e67270

[68] Gholizadeh Shamasbi S Dehgan P Mohammad-Alizadeh Charandabi S Aliasgarzadeh A Mirghafourvand M. The effect of resistant dextrin as a prebiotic on metabolic parameters and androgen level in women with polycystic ovarian syndrome: a randomized, triple-blind, controlled, clinical trial. European journal of nutrition. (2019) 58:629–40. 29480399 10.1007/s00394-018-1648-7

[69] De Munter JSL Hu FB Spiegelman D Franz M Van Dam RM. Whole grain, bran, and germ intake and risk of type 2 diabetes: a prospective cohort study and systematic review. PLoS Med. (2007) 4:e261. doi: 10.1371/journal.pmed.0040261, PMID: 17760498 PMC1952203

[70] Lerchbaum E Trummer C Theiler-Schwetz V Kollmann M Wölfler M Pilz S . Effects of vitamin D supplementation on body composition and metabolic risk factors in men: a randomized controlled trial. Nutrients. (2019) 11:1894. doi: 10.3390/nu11081894, PMID: 31416155 PMC6723889

[71] Niklowitz P Rothermel J Lass N Barth A Reinehr T. Link between chemerin, central obesity, and parameters of the Metabolic Syndrome: findings from a longitudinal study in obese children participating in a lifestyle intervention. Int J Obes. (2018) 42:1743–52. doi: 10.1038/s41366-018-0157-3, PMID: 30030480

[72] Goldstein EJ Tyrrell KL Citron DM. Lactobacillus species: taxonomic complexity and controversial susceptibilities. Clin Infect Dis. (2015) 60:S98–S107. doi: 10.1093/cid/civ072, PMID: 25922408

[73] Millette M Nguyen A Amine KM Lacroix M. Gastrointestinal survival of bacteria in commercial probiotic products. Int J Probiot Prebiot. (2013) 8:149.

[74] Domig K Kiss H Petricevic L Viernstein H Unger F Kneifel W. Strategies for the evaluation and selection of potential vaginal probiotics from human sources: an exemplary study. Benefic Microbes. (2014) 5:263–72. doi: 10.3920/BM2013.0069, PMID: 24675230

[75] Azaïs-Braesco V Bresson J Guarner F Corthier G. Not all lactic acid bacteria are probiotics,… but some are. Br J Nutr. (2010) 103:1079–81. doi: 10.1017/S0007114510000723, PMID: 20230653

[76] Lau CS Chamberlain RS. Probiotics are effective at preventing *Clostridium difficile*-associated diarrhea: a systematic review and meta-analysis. Int J Gen Med. (2016) 9:27–37. doi: 10.2147/IJGM.S98280, PMID: 26955289 PMC4769010

[77] Mcfarland LV. An observation on inappropriate probiotic subgroup classifications in the meta-analysis by Lau and Chamberlain. Int J Gen Med. (2016) 9:333–6. doi: 10.2147/IJGM.S119970, PMID: 27757047 PMC5053372

[78] Sun J Buys NJ. Glucose- and glycaemic factor-lowering effects of probiotics on diabetes: a meta-analysis of randomised placebo-controlled trials. Br J Nutr. (2016) 115:1167–77. doi: 10.1017/S0007114516000076, PMID: 26899960

[79] Frost G Sleeth ML Sahuri-Arisoylu M Lizarbe B Cerdan S Brody L . The short-chain fatty acid acetate reduces appetite via a central homeostatic mechanism. Nat Commun. (2014) 5:3611. doi: 10.1038/ncomms4611, PMID: 24781306 PMC4015327

[80] Zhuang G Liu X-M Zhang Q-X Tian F-W Zhang H Zhang H-P . Research advances with regards to clinical outcome and potential mechanisms of the cholesterol-lowering effects of probiotics. Clin Lipidol. (2012) 7:501–7. doi: 10.2217/clp.12.40

[81] Arab A Hossein-Boroujerdi M Moini A Sepidarkish M Shirzad N Karimi E. Effects of probiotic supplementation on hormonal and clinical outcomes of women diagnosed with polycystic ovary syndrome: A double-blind, randomized, placebo-controlled clinical trial. J Funct Foods. (2022) 96:105203. doi: 10.1016/j.jff.2022.105203

[82] Darvishi S Rafraf M Asghari-Jafarabadi M Farzadi L. Synbiotic supplementation improves metabolic factors and obesity values in women with polycystic ovary syndrome independent of affecting apelin levels: a randomized double-blind placebo-controlled clinical trial. Int J Fertil Sterility. (2021) 15:51. doi: 10.22074/ijfs.2021.6186, PMID: 33497048 PMC7838763

