# Supplementary material for: The effects of pro-, pre-, and synbiotics supplementation on polycystic ovary syndrome: an umbrella review of meta-analyses of randomized controlled trials
Source: Front Nutr. 2023 Sep 27;10:1178842. doi: 10.3389/fnut.2023.1178842 (PMC10565230; doi:10.3389/fnut.2023.1178842)
Supplement: Supplementary file 1 [file Data_Sheet_1.docx]

**The effects of pro-, pre- and synbiotics supplementation and polycystic ovary syndrome: an umbrella review of meta-analyses of randomized controlled trials**

*Sepide Talebi^1^, Sheida Zeraattalab-Motlagh^2^, Yahya Jalilpiran^1^, Nasim Payandeh^2^, Shakila Ansari^3^, Hamed Mohammadi^1^, Kurosh Djafarian^1^, Mahsa Ranjbar^1^, Sara Sadeghi^1^, Mahdiyeh Taghizadeh^1^, Sakineh Shab-Bidar^2^*

Online Supplementary Material

Supplementary data includes 8 supplementary Tables and 26 supplementary figures.

| **Supplementary Table 1.** Search strategies including the key terms and the queries for each database | |
| --- | --- |
| **Database**  **3/22/2023**  **(March 22, 2023)** | **Key terms and the queries** |
| PubMed | #1 ("Probiotics"[Title/Abstract] OR "Synbiotics"[Title/Abstract] OR "Prebiotics"[Title/Abstract] OR "Inulin"[Title/Abstract] OR "resistant dextrin"[Title/Abstract] OR "Microbiota"[Title/Abstract] OR "Microbiotas"[Title/Abstract] OR "Microbiome"[Title/Abstract] OR "Microbiomes"[Title/Abstract] OR "gut microflora"[Title/Abstract] OR "human microbiome"[Title/Abstract] OR "human microbiomes"[Title/Abstract] OR "microbiomes human"[Title/Abstract] OR "microbiome human"[Title/Abstract] OR "Bacteroides"[Title/Abstract] OR "Bacteroidetes"[Title/Abstract] OR "Bifidobacterium"[Title/Abstract] OR "Eubacterium"[Title/Abstract] OR "Clostridium"[Title/Abstract] OR "Lactobacillus"[Title/Abstract] OR "Fusobacterium"[Title/Abstract] OR "Firmicutes"[Title/Abstract] OR ("Probiotics"[MeSH Terms] OR "Synbiotics"[MeSH Terms] OR "Prebiotics"[MeSH Terms] OR "Inulin"[MeSH Terms] OR "Microbiota"[MeSH Terms] OR "Gastrointestinal Microbiome"[MeSH Terms] OR "Bacteroidetes"[MeSH Terms] OR "Bifidobacterium"[MeSH Terms] OR "Eubacterium"[MeSH Terms] OR "Clostridium"[MeSH Terms] OR "Lactobacillus"[MeSH Terms] OR "Fusobacterium"[MeSH Terms] OR "Firmicutes"[MeSH Terms]))  #2 ("Polycystic Ovary Syndrome"[MeSH Terms] OR ("polycystic ovarian syndrome"[Title/Abstract] OR "PCOS"[Title/Abstract]))  #3 "Meta-Analysis"[Title/Abstract] OR "meta-analyses"[Title/Abstract] OR "Meta-Analysis"[Title/Abstract] OR "meta-analyze"[Title/Abstract] OR "Systematic Review"[Title/Abstract] OR "Systematic Review"[Publication Type] OR "Systematic Reviews as Topic"[MeSH Terms] OR "Meta-Analysis as Topic"[MeSH Terms] OR "Meta-Analysis"[Publication Type]  #4 #1 AND #2 AND #3 |
| Web of Science (ISI) | #1 TOPIC: "probiotics" OR TOPIC: "synbiotics" OR TOPIC: "prebiotics" OR TOPIC: TOPIC: "Inulin" OR TOPIC: "Resistant Dextrin" OR TOPIC: "Microbiota" OR TOPIC: "Microbiotas" OR TOPIC: "Microbiome" OR TOPIC: "Microbiomes" OR TOPIC: "Gut microflora" OR TOPIC: "Human Microbiome" OR TOPIC: "Human Microbiomes" OR TOPIC: "Microbiomes, Human" OR TOPIC: "Microbiome, Human" OR TOPIC: "Bacteroides" OR TOPIC: "Bacteroidetes" OR TOPIC: "Bifidobacterium" OR TOPIC: "Eubacterium" OR TOPIC: "Clostridium" OR TOPIC: "Lactobacillus" OR TOPIC: "Fusobacterium" OR TOPIC: "Firmicutes"  #2 TOPIC: ("Polycystic Ovary Syndrome") OR TOPIC: (“PCOS”)  #3 TOPIC: ( "meta-analyses") OR TOPIC: ("meta-analysis") OR TOPIC: (" meta-analyze") OR TOPIC: (" meta analysis") OR TOPIC: (" Systematic Review")  #4 #1 AND #2 AND #3 |
| Scopus | #1 ( TITLE-ABS-KEY ( probiotics ) OR TITLE-ABS-KEY ( synbiotics ) OR TITLE-ABS-KEY ( prebiotics ) OR TITLE-ABS-KEY ( inulin ) OR TITLE-ABS-KEY ( resistant AND dextrin ) OR TITLE-ABS-KEY ( microbiota ) OR TITLE-ABS-KEY ( microbiotas ) OR TITLE-ABS-KEY ( microbiome ) OR TITLE-ABS-KEY ( microbiomes ) OR TITLE-ABS-KEY ( "Gut microflora" ) OR TITLE-ABS-KEY ( "Human Microbiome" ) OR TITLE-ABS-KEY ( "Human Microbiomes" ) OR TITLE-ABS-KEY ( "Microbiomes, Human" ) OR TITLE-ABS-KEY ( "Microbiome, Human" ) OR TITLE-ABS-KEY ( bacteroides ) OR TITLE-ABS-KEY ( bacteroidetes ) OR TITLE-ABS-KEY ( bifidobacterium ) OR TITLE-ABS-KEY ( eubacterium ) OR TITLE-ABS-KEY ( clostridium ) OR TITLE-ABS-KEY ( lactobacillus ) OR TITLE-ABS-KEY ( fusobacterium ) OR TITLE-ABS-KEY ( firmicutes ) )  #2 ( TITLE-ABS-KEY ( "Polycystic ovarian syndrome " ) OR TITLE-ABS-KEY ( "PCOS" ) )  #3 ( TITLE-ABS-KEY ( "meta-analyses" ) OR TITLE-ABS-KEY ( "meta-analysis" ) OR TITLE-ABS-KEY ( "meta-analyze" ) OR TITLE-ABS-KEY ( "meta analysis" ) OR TITLE-ABS-KEY ( "Systematic Review" ) )  #4 #1 AND #2 AND #3 |

23 + 24 +44

**Supplementary Table 2.** Cochrane Risk of Bias Assessment

| Study | Random Sequence Generation | Allocation concealment | Selective outcome reporting | Other sources of bias | Blinding of participants and personnel | Blinding of outcome assessment | Incomplete outcome data |
| --- | --- | --- | --- | --- | --- | --- | --- |
| Samimi et al. (Iran,2018) (Samimi et al., 2019) | L | L | L | L | L | H | L |
| Sevda et al. (Iran,2018) (Shamasbi et al., 2018) | L | L | L | L | L | L | L |
| Shoaei et al. (Iran, 2015) (Shoaei et al., 2015) | L | U | L | L | L | L | L |
| Esmaeilinezhad et al. (Iran, 2019) (Esmaeilinezhad et al., 2020) | L | L | L | L | L | L | L |
| Karamali et al. (Iran,2018) (Karamali et al., 2018) | L | L | L | L | L | U | L |
| Karimi et al (Iran,2020) (Karimi et al., 2020) | L | L | L | L | L | U | L |
| Nasri et al (Iran,2018) (Nasri et al., 2018) | L | L | L | L | L | U | L |
| Rashad et al (Egypt,2017) (Rashad et al., 2017) | H | H | L | H | H | H | H |
| Ahmadi et al. (Iran, 2017) (Ahmadi et al., 2017) | L | L | L | L | U | U | L |
| Esmaeilinezhad et al. (Iran, 2018) (Esmaeilinezhad et al., 2019) | L | L | L | L | L | L | L |
| Ghanei et al. (Iran, 2018) (Ghanei et al., 2018) | L | L | L | L | U | U | L |
| Gholizadehshamasbi et al. (Iran, 2018) (Shamasbi et al., 2019) | L | L | L | L | L | L | L |

L, low risk of bias; H, high risk of bias; U, unclear risk of bias.

| Supplementary Table 3. Minimal clinically important differences (MCID) for outcomes^1^. | |
| --- | --- |
| Variable (ref) | MCID (unit) |
| Body weight (Ge et al., 2020) | 2.5 kg |
| Body mass index (Ge et al., 2020) | 0.95 kg/m^2^ |
| Waist circumference (Jovanovski et al., 2020) | 2 cm |
| Fasting insulin (Goldenberg et al., 2021) | 5 pmol/L |
| Fasting plasma glucose (Goldenberg et al., 2021) | 1.6 mmol/L |
| Total cholesterol (Goldenberg et al., 2021) | 0.26 mmol/L (10.05 mg/dL) |
| Low density lipoprotein cholesterol (Goldenberg et al., 2021) | 0.1 mmol/L (3.87 mg/dL) |
| High density lipoprotein cholesterol (Goldenberg et al., 2021) | 0.1 mmol/L (3.87 mg/dL) |
| Triglycerides (Goldenberg et al., 2021) | 0.09 mmol/L (7.97 mg/dL) |
| Very low density lipoprotein (VLDL) (Goldenberg et al., 2021) | -4.3 mg/dl |
| C-reactive protein (Goldenberg et al., 2021) | 0.5 mg/L |
| Homeostasis model assessment of insulin resistance (Goldenberg et al., 2021) | 0.05 |
| QUICKI (Goldenberg et al., 2021) | 0.07 |
| Malondialdehyde (MDA) (Norman et al., 2003) | 0.59 mmol/mL |
| Total Antioxidant Capacity (TAC) (Norman et al., 2003) | 0.08 mmol/l |
| Nitric oxide (NO) (Norman et al., 2003) | 1.7 μmol/l |
| Glutathione (GSH) (Norman et al., 2003) | -37.8 μmol/l |
| Total testosterone (Revicki et al., 2008) | -0.2 ng/ml |
| Sex hormone binding globulin (Norman et al., 2003) | 15.95 nmol/l |
| Dehydroepiandrosterone (Norman et al., 2003) | 0.6 μg/mL |
| Ferriman-Gallwey (Norman et al., 2003) | -2.15 |
| ^1^ Numbers in parenthesis refer to supplemental references. | |

**Supplementary Table 4**: Reason for exclusion of retrieved articles

| References | Reason for exclusion |
| --- | --- |
| 1. Zhu, Z., X. Pengbin, and H. Yaqiong. 2020. Effects of vitamin D and probiotics supplementation on bacterial diversity, metabolism and hormone level in patients with polycystic ovary syndrome. Chinese Journal of Microecology 32 (3):317–21.  2. Chen, Y., Z. Minghui, and W. Channi. 2018. Effect of probiotic supplementation on blood sugar and lipids in patients with polycystic ovary syndrome. International Journal of Gynecology & Obstetrics 45(2):199–202 | **Full text of articles not found** |
| 3. Jamilian, M., S. Mansury, F. Bahmani, Z. Heidar, E. Amirani, and Z.Asemi. 2018. The effects of probiotic and selenium co-supplementation on parameters of mental health, hormonal profiles, and biomarkers of inflammation and oxidative stress in women with polycystic ovary syndrome. Journal of Ovarian Research 11 (1): 80.doi: 10.1186/s13048-018-0457-1.  4. Shabani, A., M. Noshadian, M. Jamilian, M. Chamani, S. Mohammadi,and Z. Asemi. 2018. The effects of a novel combination of selenium and probiotic on weight loss, glycemic control and markers of cardio-metabolic risk in women with polycystic ovary syndrome. Journal of Functional Foods 46:329–34. doi: 10.1016/j.jff.2018.04.071.  5. Ostadmohammadi, V., M. Jamilian, F. Bahmani, and Z. Asemi. 2019. Vitamin D and probiotic co-supplementation affects mental health, hormonal, inflammatory and oxidative stress parameters in women with polycystic ovary syndrome. Journal of Ovarian Research 12 (1): 5. doi: 10.1186/s13048-019-0480-x. | **Probiotic was used in combination with other medication**  **(co-supplement)** |

| **Author, year** | **Q1** | **Q2** | **Q3** | **Q4** | **Q5** | **Q6** | **Q7** | **Q8** | **Q9** | **Q10** | **Q11** | **Q12** | **Q13** | **Q14** | **Q15** | **Q16** | **Level of evidence** |
| --- | --- | --- | --- | --- | --- | --- | --- | --- | --- | --- | --- | --- | --- | --- | --- | --- | --- |
| Cozzolino et al., 2020 (Cozzolino et al., 2020) | Yes | Yes | Yes | PY | Yes | Yes | PY | Yes | Yes | Yes | Yes | Yes | No | Yes | Yes | Yes | Low |
| Gholizadeh Shamasbi et al., 2020 (Shamasbi et al., 2020) | Yes | No | Yes | PY | Yes | Yes | PY | Yes | Yes | Yes | Yes | Yes | No | Yes | No | Yes | Critically low |
| Heshmati et al., 2018 (Heshmati et al., 2019) | Yes | No | Yes | PY | Yes | Yes | Yes | Yes | Yes | No | Yes | Yes | No | Yes | No | Yes | Critically low |
| Hadi et al., 2020 (Hadi et al., 2020) | Yes | Yes | Yes | PY | Yes | Yes | PY | Yes | Yes | Yes | Yes | Yes | No | No | Yes | Yes | Critically low |
| Kazemi et al., 2021 (Kazemi et al., 2020) | Yes | Yes | Yes | PY | Yes | Yes | PY | Yes | Yes | Yes | Yes | Yes | Yes | Yes | Yes | Yes | High |
| Li et al., 2021 (Li et al., 2021) | Yes | Yes | Yes | PY | Yes | Yes | Yes | Yes | Yes | Yes | Yes | Yes | Yes | Yes | Yes | Yes | High |
| Miao et al., 2020 (Miao et al., 2021) | Yes | No | Yes | PY | Yes | Yes | PY | Yes | Yes | Yes | Yes | Yes | Yes | Yes | Yes | Yes | Low |
| Tabrizi et al., 2019 (Tabrizi et al., 2019) | Yes | No | Yes | PY | Yes | Yes | PY | Yes | Yes | Yes | Yes | Yes | No | Yes | Yes | Yes | Low |
| Zangh et al. 2023 (Zhang et al., 2023) | Yes | Yes | Yes | PY | Yes | Yes | PY | Yes | Yes | Yes | Yes | Yes | No | Yes | Yes | Yes | Low |

**Supplementary Table 5.** AMSTAR quality assessment of meta-analyses of randomized controlled trials.

PY, partially yes. Q1: Did the research questions and inclusion criteria for the review include the components of PICO?, Q2: 2. Did the report of the review contain an explicit statement that the review methods were established prior to the conduct of the review and did the report justify any significant deviations from the protocol?; Q3, Did the review authors explain their selection of the study designs for inclusion in the review?; Q4, Did the review authors use a comprehensive literature search strategy?; Q5, Did the review authors perform study selection in duplicate?; Q6, Did the review authors perform data extraction in duplicate?; Q7, Did the review authors provide a list of excluded studies and justify the exclusions?; Q8, Did the review authors describe the included studies in adequate detail?; Q9, Did the review authors use a satisfactory technique for assessing the risk of bias?; Q10, Did the review authors report on the sources of funding?; Q11, Did the review authors use appropriate methods for statistical combination of results?; Q12, Did the review authors assess the potential impact of RoB in individual studies on the results?; Q13, Did the review authors account for RoB in individual studies when interpreting/ discussing the results of the review?; Q14, Did the review authors provide a satisfactory explanation for, and discussion of, any heterogeneity?; Q15, Did the review authors carry out an adequate investigation of publication bias?; Q16, Did the review authors report any potential sources of conflict of interest?

| **Supplementary Table 6: GRADE evidence table for the effects of probiotic supplementation in women with PCOS.** | | | | | | | | | | | |
| --- | --- | --- | --- | --- | --- | --- | --- | --- | --- | --- | --- |
| Certainty assessment | | | | | | | No of patients | | Effect | Certainty | Importance |
| No of studies | Design | Risk of bias | Inconsistency | Indirectness | Imprecision | Other  considerations | Treatment group | Control group | WMD (95%CI) |  |  |
| Body weight | | | | | | | | | | | |
| 4 | Randomized trials | not serious | Serious ^a^ | not serious | Serious ^b^ | none | 134 | 134 | MD **0.25 kg higher** (1.37 lower to 1.88 higher) | ⨁⨁◯◯ Low | IMPORTANT |
| BMI | | | | | | | | | | | |
| 5 | Randomized trials | not serious | Serious ^c^ | not serious | Serious ^d^ | none | 194 | 174 | MD **0.44 kg/m2 higher** (0.23 lower to 1.12 higher) | ⨁⨁◯◯ Low | IMPORTANT |
| Waist circumference | | | | | | | | | | | |
| 2 | Randomized trials | not serious | not serious ^e^ | Serious ^f^ | Serious ^g^ | none | 74 | 74 | MD **0.86 cm higher** (0.38 higher to 1.33 higher) | ⨁⨁◯◯ Low | IMPORTANT |
| Hip circumference | | | | | | | | | | | |
| 1 | Randomized trials | not serious | not serious | Serious ^h^ | Serious ^i^ | none | 44 | 44 | MD **0.6 cm lower** (1.09 lower to 2.29 higher) | ⨁⨁◯◯ Low | IMPORTANT |
| Fasting glucose concentration | | | | | | | | | | | |
| 4 | Randomized trials | not serious | Serious ^j^ | not serious | Serious ^k^ | none | 170 | 150 | MD **7.05 mg/dL lower** (13.6 lower to 0.51 lower) | ⨁⨁◯◯ Low | IMPORTANT |
| Fasting insulin | | | | | | | | | | | |
| 4 | Randomized trials | not serious | Serious ^l^ | not serious | Serious ^m^ | none | 170 | 150 | MD **0.4 µIU/ml lower** (0.94 lower to 0.15 higher) | ⨁⨁◯◯ Low | IMPORTANT |
| HOMA-IR | | | | | | | | | | | |
| 4 | Randomized trials | not serious | not serious ^n^ | not serious | Serious ^o^ | none | 170 | 150 | MD **0.29 lower** (0.57 lower to 0.02 lower) | ⨁⨁⨁◯ Moderate | IMPORTANT |
| QUICKI | | | | | | | | | | | |
| 3 | Randomized trials | not serious | Serious ^p^ | not serious | Serious ^q^ | none | 110 | 110 | MD **0.01 higher** (0 to 0.01 higher) | ⨁⨁◯◯ Low | IMPORTANT |
| Triglycerides | | | | | | | | | | | |
| 3 | Randomized trials | not serious | Serious ^r^ | not serious | Serious ^s^ | none | 134 | 114 | MD **39.51 mg/dl lower** (95.42 lower to 16.4 higher) | ⨁⨁◯◯ Low | IMPORTANT |
| Very low density lipoprotein | | | | | | | | | | | |
| 1 | Randomized trials | not serious | not serious | Serious ^t^ | Serious ^u^ | none | 30 | 30 | MD **50.4 mg/dl lower** (9.91 lower to 0.89 lower) | ⨁⨁◯◯ Low | IMPORTANT |
| Total cholesterol | | | | | | | | | | | |
| 3 | Randomized trials | not serious | Serious ^v^ | not serious | Serious ^w^ | none | 134 | 114 | MD **4.29 mg/dl lower** (19.62 lower to 11.04 higher) | ⨁⨁◯◯ Low | IMPORTANT |
| HDL cholesterol | | | | | | | | | | | |
| 3 | Randomized trials | not serious | Serious ^x^ | not serious | Serious ^y^ | none | 134 | 114 | MD **3.8 mg/dl lower** (8.93 lower to 1.32 higher) | ⨁⨁◯◯ Low | IMPORTANT |
| LDL cholesterol | | | | | | | | | | | |
| 3 | Randomized trials | not serious | Serious ^z^ | not serious | Serious ^aa^ | none | 134 | 114 | MD **6.2 mg/dl higher** (5.38 lower to 17.77 higher) | ⨁⨁◯◯ Low | IMPORTANT |
| Total testosterone | | | | | | | | | | | |
| 1 | Randomized trials | not serious | not serious | Serious ^ab^ | Serious ^ac^ | none | 30 | 30 | MD **0.4 ng/dl lower** (0.73 lower to 0.07 lower) | ⨁⨁◯◯ Low | IMPORTANT |
| Dehydroepiandrosterone sulfate | | | | | | | | | | | |
| 1 | Randomized trials | not serious | not serious | Serious ^ad^ | Serious ^ae^ | none | 30 | 30 | MD **0.17 μg/mL higher** (0.01 lower to 0.35 higher) | ⨁⨁◯◯ Low | IMPORTANT |
| Sex hormone-binding globulin | | | | | | | | | | | |
| 1 | Randomized trials | not serious | not serious | serious^af^ | Serious ^ag^ | none | 30 | 30 | MD **25.4 nmol/l higher** (12.5 higher to 38.3 higher) | ⨁⨁◯◯ Low | IMPORTANT |
| C-reactive protein | | | | | | | | | | | |
| 2 | Randomized trials | not serious | Serious ^ah^ | Serious ^ai^ | Serious ^aj^ | none | 80 | 80 | MD **0.92 mg/l higher** (0.57 lower to 2.4 higher) | ⨁◯◯◯ Very low | IMPORTANT |
| high-sensitivity C-reactive protein | | | | | | | | | | | |
| 3 | Randomized trials | not serious | Serious ^ak^ | not serious | Serious ^al^ | none | 120 | 100 | MD **0.5 mg/l higher** (1.92 lower to 2.93 higher) | ⨁⨁◯◯ Low | IMPORTANT |
| Nitric oxide | | | | | | | | | | | |
| 1 | Randomized trials | not serious | not serious | Serious ^am^ | Serious ^an^ | none | 30 | 30 | MD **1.8 μmol/l higher** (1.49 lower to 5.09 higher) | ⨁⨁◯◯ Low | IMPORTANT |
| Total antioxidant capacity | | | | | | | | | | | |
| 1 | Randomized trials | not serious | not serious | Serious ^ao^ | Serious ^ap^ | none | 30 | 30 | MD **107.1 mmol/l higher** (8.95 higher to 205.25 higher) | ⨁⨁◯◯ Low | IMPORTANT |
| GSH | | | | | | | | | | | |
| 1 | Randomized trials | not serious | not serious | Serious ^aq^ | Serious ^ar^ | none | 30 | 30 | MD **70.8 μmol/l higher** (5.39 lower to 146.99 higher) | ⨁⨁◯◯ Low | IMPORTANT |
| Malondialdehyde | | | | | | | | | | | |
| 1 | Randomized trials | not serious | not serious | Serious ^as^ | Serious ^at^ | none | 30 | 30 | MD **1.1 μmol/l higher** (0.59 higher to 1.61 higher) | ⨁⨁◯◯ Low | IMPORTANT |
| Hirsutism score | | | | | | | | | | | |
| 1 | Randomized trials | not serious | not serious | Serious ^au^ | Serious ^av^ | none | 30 | 30 | MD **1.5 lower** (2.15 lower to 0.85 lower) | ⨁⨁◯◯ Low | IMPORTANT |

CI: confidence interval; MD: mean difference

Explanations

a. Serious inconsistency since I2 = 97.1%, Phet <0.001. Downgraded.

b. Optimal information size did not meet. The effect size (WMD: 0.25) did not surpass the minimal clinically important difference for body weight (MCID: 2.5 kg). Downgraded.

c. Serious inconsistency since I2 = 94.3%, Phet <0.001. Downgraded.

d. Optimal information size met. The effect size (WMD: 0.44) did not surpass the minimal clinically important difference for BMI (MCID: 0.95 kg/m2). Downgraded.

e. Not serious inconsistency since I2 = 0%, Phet = 0.496. Not downgraded.

f. Serious indirectness since only 2 trials were available. Downgraded.

g. Optimal information size did not meet. The effect size (WMD: 0.86) did not surpass the minimal clinically important difference for WC (MCID: 2 cm). Downgraded.

h. Serious indirectness since only 1 trial was available. Downgraded.

i. Optimal information size did not meet. The effect size (WMD: -0.60) did not surpass the minimal clinically important difference for hip circumference (MCID: -3.60 cm). Downgraded.

j. Serious inconsistency since I2 = 93.7%, Phet <0.001. Downgraded.

k. Optimal information size met. The effect size (WMD: - 7.05) did not surpass the minimal clinically important difference for FBS (MCID: -28.5 mg/dL). Downgraded.

l. Serious inconsistency since I2 = 82.6%, Phet =0.01. Downgraded.

m. Optimal information size met. The effect size (WMD: -0.40) did not surpass the minimal clinically important difference for FI (MCID: -0.72 µIU/ml). Downgraded.

n. Not serious inconsistency since I2 = 33.8%, Phet = 0.209. Not downgraded.

o. Optimal information size met. The effect size (WMD: -0.29) surpassed the minimal clinically important difference for HOMA-IR (MCID: -0.05), but the upper bound of the 95%CI overlapped the MCID for HOMA-IR (95%CI: -0.57, -0.02). Downgraded.

p. Serious inconsistency since I2 = 62%, Phet =0.072. Downgraded.

q. Optimal information size did not meet. The effect size (WMD: 0.01) did not surpass the minimal clinically important difference for QUICKI (MCID: 0.07). Downgraded.

r. Serious inconsistency since I2 = 97.5%, Phet <0.001. Downgraded.

s. Optimal information size did not meet. The effect size (WMD: -39.51) surpassed the minimal clinically important difference for TG (MCID: -8 mg/dl), but the upper bound of the 95%CI overlapped the MCID for TG (95%CI: -95.42, 16.40). Downgraded.

t. Serious indirectness since only 1 trial was available. Downgraded.

u. Optimal information size did not meet. The effect size (WMD: -50.40) surpassed the minimal clinically important difference for VLDL (MCID: -4.3 mg/dl), but the upper bound of the 95%CI overlapped the MCID for VLDL (95%CI: -9.91, -0.89). Downgraded.

v. Serious inconsistency since I2 = 89.4%, Phet <0.001. Downgraded.

w. Optimal information size did not meet. The effect size (WMD: -4.29) did not surpass the minimal clinically important difference for TC (MCID: -10 mg/dl). Downgraded.

x. Serious inconsistency since I2 = 53.5%, Phet =0.117. Downgraded.

y. Optimal information size did not meet. The effect size (WMD: -3.80) did not surpass the minimal clinically important difference for HDL cholesterol (MCID: -3.87 mg/dl). Downgraded.

z. Serious inconsistency since I2 = 99%, Phet <0.001. Downgraded.

aa. Optimal information size did not meet. The effect size (WMD: 6.20) surpassed the minimal clinically important difference for LDL cholesterol (MCID: 3.87 mg/dl), but the lower bound of the 95%CI overlapped the MCID for LDL cholesterol (95%CI: -5.38, 17.77). Downgraded.

ab. Serious indirectness since only 1 trial was available. Downgraded.

ac. Optimal information size did not meet. The effect size (WMD: -0.40) surpassed the minimal clinically important difference for TT (MCID: -0.2 ng/ml), but the upper bound of the 95%CI overlapped the MCID for TT (95%CI: -0.73, -0.07). Downgraded.

ad. Serious indirectness since only 1 trial was available. Downgraded.

ae. Optimal information size did not meet. The effect size (WMD: 0.17) did not surpass the minimal clinically important difference for DHEAS (MCID: 0.6 μg/mL). Downgraded.

af. Serious indirectness since only 1 trial was available. Downgraded.

ag. Optimal information size did not meet. The effect size (WMD: 25.40) surpassed the minimal clinically important difference for SHBG (MCID: 15.95 nmol/l), but the lower bound of the 95%CI overlapped the MCID for SHBG (95%CI: 12.50, 38.30). Downgraded.

ah. Serious inconsistency since I2 = 73.3%, Phet =0.053. Downgraded.

ai. Serious indirectness since only 2 trials were available. Downgraded.

aj. Optimal information size did not meet. The effect size (WMD: 0.92) surpassed the minimal clinically important difference for CRP (MCID: 0.5 mg/l), but the upper bound of the 95%CI overlapped the MCID for CRP (95%CI: -0.57, 2.40). Downgraded.

ak. Serious inconsistency since I2 = 99.1%, Phet <0.001. Downgraded.

al. Optimal information size did not meet. The effect size (WMD: 0.50) did not surpass the minimal clinically important difference for hs-CRP (MCID: 0.5 mg/l). Downgraded.

am. Serious indirectness since only 1 trial was available. Downgraded.

an. Optimal information size did not meet. The effect size (WMD: 1.80) surpassed the minimal clinically important difference for NO (MCID: 1.7 μmol/l), but the lower bound of the 95%CI overlapped the MCID for NO (95%CI: -1.49, 5.09). Downgraded.

ao. Serious indirectness since only 1 trial was available. Downgraded.

ap. Optimal information size did not meet. The effect size (WMD: 107.10) surpassed the minimal clinically important difference for TAC (MCID: 0.08 mmol/l) and the lower and upper bounds of the 95%CI did not overlap the MCID for TAC (95%CI: 8.95, 205.25). Downgraded.

aq. Serious indirectness since only 1 trial was available. Downgraded.

ar. Optimal information size did not meet. The effect size (WMD: 70.80) surpassed the minimal clinically important difference for GSH (MCID: 37.8 μmol/l), but the lower bound of the 95%CI overlapped the MCID for GSH (95%CI: -5.39, 146.99). Downgraded.

as. Serious indirectness since only 1 trial was available. Downgraded.

at. Optimal information size did not meet. The effect size (WMD: 1.10) surpassed the minimal clinically important difference for MDA (MCID: 0.59 μmol/l), but the lower bound of the 95%CI overlapped the MCID for MDA (95%CI: 0.59, 1.61). Downgraded.

au. Serious indirectness since only 1 trial was available. Downgraded.

av. Optimal information size did not meet. The effect size (WMD: -1.50) did not surpass the minimal clinically important difference for mf-G (MCID: -2.15). Downgraded.

| **Supplementary Table 7: GRADE evidence table for the effects of synbiotic supplementation in women with PCOS.** | | | | | | | | | | | |
| --- | --- | --- | --- | --- | --- | --- | --- | --- | --- | --- | --- |
| Certainty assessment | | | | | | | No of patients | | Effect | Certainty | Importance |
| No of studies | Design | Risk of bias | Inconsistency | Indirectness | Imprecision | Other  considerations | Treatment group | Control group | WMD (95%CI) |  |  |
| Body weight | | | | | | | | | | | |
| 3 | Randomized trials | not serious | not serious ^a^ | not serious | Serious ^b^ | none | 106 | 106 | MD **0.19 kg lower** (0.79 lower to 0.42 higher) | ⨁⨁⨁◯ Moderate | IMPORTANT |
| BMI | | | | | | | | | | | |
| 3 | Randomized trials | not serious | Serious ^c^ | not serious | Serious ^d^ | none | 106 | 106 | MD **0.06 kg/m2 lower** (0.33 lower to 0.21 higher) | ⨁⨁◯◯ Low | IMPORTANT |
| Waist circumference | | | | | | | | | | | |
| 1 | Randomized trials | not serious | not serious ^e^ | Serious ^f^ | Serious ^g^ | none | 46 | 46 | MD **2.7 cm lower** (4.28 lower to 1.12 lower) | ⨁⨁◯◯ Low | IMPORTANT |
| Hip circumference | | | | | | | | | | | |
| 1 | Randomized trials | not serious | not serious ^h^ | Serious ^i^ | Serious ^j^ | none | 46 | 46 | MD **0.03 cm lower** (1.75 lower to 1.69 higher) | ⨁⨁◯◯ Low | IMPORTANT |
| Fasting glucose concentration | | | | | | | | | | | |
| 2 | Randomized trials | not serious | not serious ^k^ | Serious ^l^ | Serious ^m^ | none | 76 | 76 | MD **1.94 mg/dl lower** (3.95 lower to 0.08 higher) | ⨁⨁◯◯ Low | IMPORTANT |
| Fasting insulin | | | | | | | | | | | |
| 2 | Randomized trials | not serious | not serious ^n^ | Serious ^o^ | Serious ^p^ | none | 76 | 76 | MD **0.9 µIU/ml lower** (1.24 lower to 0.57 lower) | ⨁⨁◯◯ Low | IMPORTANT |
| HOMA-IR | | | | | | | | | | | |
| 2 | Randomized trials | not serious | not serious ^q^ | Serious ^r^ | Serious ^s^ | none | 76 | 76 | MD **0.82 lower** (1.09 lower to 0.56 lower) | ⨁⨁◯◯ Low | IMPORTANT |
| QUICKI | | | | | | | | | | | |
| 2 | Randomized trials | not serious | Serious ^t^ | Serious ^u^ | Serious ^v^ | none | 76 | 76 | MD **0.01 higher** (0 to 0.01 higher) | ⨁◯◯◯ Very low | IMPORTANT |
| Triglycerides | | | | | | | | | | | |
| 2 | Randomized trials | not serious | Serious ^w^ | Serious ^x^ | Serious ^y^ | none | 74 | 72 | MD **15.37 mg/dl lower** (22.53 lower to 8.21 lower) | ⨁◯◯◯ Very low | IMPORTANT |
| Very low density lipoprotein | | | | | | | | | | | |
| 1 | Randomized trials | not serious | not serious | Serious ^z^ | Serious ^aa^ | none | 30 | 30 | MD **4.4 mg/dl lower** (7.19 lower to 1.61 lower) | ⨁⨁◯◯ Low | IMPORTANT |
| Total cholesterol | | | | | | | | | | | |
| 2 | Randomized trials | not serious | not serious ^ab^ | Serious ^ac^ | Serious ^ad^ | none | 74 | 72 | MD **10.57 mg/dl lower** (20.83 lower to 0.31 lower) | ⨁⨁◯◯ Low | IMPORTANT |
| HDL cholesterol | | | | | | | | | | | |
| 2 | Randomized trials | not serious | Serious ^ae^ | Serious ^af^ | Serious ^ag^ | none | 74 | 72 | MD **21.58 mg/dl lower** (41.62 lower to 1.53 lower) | ⨁◯◯◯ Very low | IMPORTANT |
| LDL cholesterol | | | | | | | | | | | |
| 2 | Randomized trials | not serious | not serious ^ah^ | Serious ^ai^ | Serious ^aj^ | none | 74 | 72 | MD **3.02 mg/dl higher** (2.57 lower to 8.62 higher) | ⨁⨁◯◯ Low | IMPORTANT |
| Total testosterone | | | | | | | | | | | |
| 2 | Randomized trials | not serious | not serious ^ak^ | Serious ^al^ | Serious ^am^ | none | 76 | 76 | MD **0.13 ng/ml lower** (0.18 lower to 0.09 lower) | ⨁⨁◯◯ Low | IMPORTANT |
| Dehydroepiandrosterone sulfate | | | | | | | | | | | |
| 1 | Randomized trials | not serious | not serious | Serious ^an^ | Serious ^ao^ | none | 30 | 30 | MD **0.3 μg/ml lower** (0.72 lower to 0.12 higher) | ⨁⨁◯◯ Low | IMPORTANT |
| Sex hormone-binding globulin | | | | | | | | | | | |
| 1 | Randomized trials | not serious | not serious | Serious ^ap^ | Serious ^aq^ | none | 30 | 30 | MD **19.3 nmol/l higher** (2.26 higher to 36.34 higher) | ⨁⨁◯◯ Low | IMPORTANT |
| high-sensitivity C-reactive protein | | | | | | | | | | | |
| 2 | Randomized trials | not serious | Serious ^ar^ | Serious ^as^ | Serious ^at^ | none | 74 | 72 | MD **0.15 mg/l lower** (0.39 lower to 0.09 higher) | ⨁◯◯◯ Very low | IMPORTANT |
| Nitric oxide | | | | | | | | | | | |
| 1 | Randomized trials | not serious | not serious | Serious ^au^ | Serious ^av^ | none | 30 | 30 | MD **5.2 μmol/l higher** (1.52 higher to 8.88 higher) | ⨁⨁◯◯ Low | IMPORTANT |
| Total antioxidant capacity | | | | | | | | | | | |
| 2 | Randomized trials | not serious | Serious ^aw^ | Serious ^ax^ | Serious ^ay^ | none | 74 | 72 | MD **0.1 mmol/l lower** (0.42 lower to 0.23 higher) | ⨁◯◯◯ Very low | IMPORTANT |
| GSH | | | | | | | | | | | |
| 1 | Randomized trials | not serious | not serious | Serious ^az^ | Serious ^ba^ | none | 30 | 30 | MD **2.6 μmol/l lower** (49.7 lower to 44.5 higher) | ⨁⨁◯◯ Low | IMPORTANT |
| Malondialdehyde | | | | | | | | | | | |
| 2 | Randomized trials | not serious | not serious ^bb^ | Serious ^bc^ | Serious ^bd^ | none | 74 | 72 | MD **0.27 μmol/l lower** (0.45 lower to 0.09 higher) | ⨁⨁◯◯ Low | IMPORTANT |
| Hirsutism score | | | | | | | | | | | |
| 1 | Randomized trials | not serious | not serious | Serious ^be^ | Serious ^bf^ | none | 30 | 30 | MD **1.2 lower** (2.11 lower to 0.29 lower) | ⨁⨁◯◯ Low | IMPORTANT |

CI: confidence interval; MD: mean difference

Explanations

a. Not serious inconsistency since I2 = 40.2%, Phet = 0.170. Not downgraded.

b. Optimal information size did not meet. The effect size (WMD: -0.19) did not surpass the minimal clinically important difference for body weight (MCID: -2.5 kg). Downgraded.

c. Serious inconsistency since I^2^ = 56.8%, Phet =0.074. Downgraded.

d. Optimal information size did not meet. The effect size (WMD: -0.06) did not surpass the minimal clinically important difference for BMI (MCID: -0.95 kg/m2). Downgraded.

e. Not serious inconsistency since I^2^ = 0%, Phet = 0.747. Not downgraded.

f. Serious indirectness since only 1 trial was available. Downgraded.

g. Optimal information size did not meet. The effect size (WMD: -2.70) surpassed the minimal clinically important difference for WC (MCID: -2 cm), but the upper bound of the 95%CI overlapped the MCID for WC (95%CI: -4.28, -1.12). Downgraded.

h. Not serious inconsistency since I^2^ = 0%, Phet = 0.964. Not downgraded.

i. Serious indirectness since only 1 trial was available. Downgraded.

j. Optimal information size did not meet. The effect size (WMD: -0.03) did not surpass the minimal clinically important difference for hip circumference (MCID: -3.60 cm). Downgraded.

k. Not serious inconsistency since I^2^ = 0%, Phet = 0.722. Not downgraded.

l. Serious indirectness since only 2 trials were available. Downgraded.

m. Optimal information size did not meet. The effect size (WMD: -1.94) did not surpass the minimal clinically important difference for FBS (MCID: -28.5 mg/dl). Downgraded.

n. Not serious inconsistency since I^2^ = 0%, Phet = 0.479. Not downgraded.

o. Serious indirectness since only 2 trials were available. Downgraded.

p. Optimal information size met. The effect size (WMD: -0.90) surpassed the minimal clinically important difference for FI (MCID: -0.72 µIU/ml), but the upper bound of the 95%CI overlapped the MCID for FI (95%CI: -1.24, -0.57). Downgraded.

q. Not serious inconsistency since I2 = 0%, Phet = 0.438. Not downgraded.

r. Serious indirectness since only 2 trials were available. Downgraded.

s. Optimal information size did not meet. The effect size (WMD: -0.82) surpassed the minimal clinically important difference for HOMA-IR (MCID: -0.05) and the lower and upper bounds of the 95%CI did not overlap the MCID for HOMA-IR (95%CI: -1.09, -0.56). Downgraded.

t. Serious inconsistency since I2 = 85.6%, Phet =0.001. Downgraded.

u. Serious indirectness since only 2 trials were available. Downgraded.

v. Optimal information size did not meet. The effect size (WMD: 0.01) did not surpass the minimal clinically important difference for QUICKI (MCID: 0.07). Downgraded.

w. Not serious inconsistency since I^2^ = 0%, Phet = 0.554. Not downgraded.

x. Serious indirectness since only 2 trials were available. Downgraded.

y. Optimal information size did not meet. The effect size (WMD: -15.37) surpassed the minimal clinically important difference for TG (MCID: -8 mg/dl), and the lower and upper bound of the 95%CI did not overlap the MCID for TG (95%CI: -22.53, -8.21). Downgraded.

z. Serious indirectness since only 1 trial was available. Downgraded.

aa. Optimal information size did not meet. The effect size (WMD: -4.40) did not surpass the minimal clinically important difference for VLDL (MCID: -4.3 mg/dl). Downgraded.

ab. Not serious inconsistency since I^2^ = 35.3%, Phet = 0.217. Not downgraded.

ac. Serious indirectness since only 2 trials were available. Downgraded.

ad. Optimal information size did not meet. The effect size (WMD: -10.57) surpassed the minimal clinically important difference for TC (MCID: -10 mg/dl), but the upper bound of the 95%CI overlapped the MCID for TC (95%CI: -20.83, -0.31). Downgraded.

ae. Serious inconsistency since I^2^ = 80.4%, Phet = 0.006. Downgraded.

af. Serious indirectness since only 2 trials were available. Downgraded.

ag. Optimal information size did not meet. The effect size (WMD: -21.58) surpassed the minimal clinically important difference for HDL cholesterol (MCID: -3.87 mg/dl), but the upper bound of the 95%CI overlapped the MCID for HDL cholesterol (95%CI: -41.62, -1.53). Downgraded.

ah. Not serious inconsistency since I^2^ = 47.0%, Phet = 0.151. Not downgraded.

ai. Serious indirectness since only 2 trials were available. Downgraded.

aj. Optimal information size did not meet. The effect size (WMD: 3.02) did not surpass the minimal clinically important difference for LDL cholesterol (MCID: 3.87 mg/dl). Downgraded.

ak. Not serious inconsistency since I^2^ = 22.7%, Phet = 0.274. Not downgraded.

al. Serious indirectness since only 2 trials were available. Downgraded.

am. Optimal information size did not meet. The effect size (WMD: -0.13) did not surpass the minimal clinically important difference for TT (MCID: -0.2 ng/ml). Downgraded.

an. Serious indirectness since only 1 trial was available. Downgraded.

ao. Optimal information size did not meet. The effect size (WMD: -0.30) did not surpass the minimal clinically important difference for DHEAS (MCID: -1.1 μg/mL). Downgraded.

ap. Serious indirectness since only 1 trial was available. Downgraded.

aq. Optimal information size did not meet. The effect size (WMD: 19.3) did not surpass the minimal clinically important difference for SHBG (MCID: 24.3 nmol/l). Downgraded.

ar. Serious inconsistency since I^2^ = 90.0%, Phet <0.001. Downgraded.

as. Serious indirectness since only 2 trials were available. Downgraded.

at. Optimal information size did not meet. The effect size (WMD: -0.15) did not surpass the minimal clinically important difference for hs-CRP (MCID: -0.5 mg/l). Downgraded.

au. Serious indirectness since only 1 trial was available. Downgraded.

av. Optimal information size did not meet. The effect size (WMD: 5.20) surpassed the minimal clinically important difference for NO (MCID: 1.7 μmol/l), but the lower bound of the 95%CI overlapped the MCID for NO (95%CI: 1.52, 8.88). Downgraded.

aw. Serious inconsistency since I^2^ = 62.5%, Phet =0.070. Downgraded.

ax. Serious indirectness since only 2 trials were available. Downgraded.

ay. Optimal information size did not meet. The effect size (WMD: -0.10) surpassed the minimal clinically important difference for TAC (MCID: 0.08 mmol/l), but the upper bounds of the 95%CI overlapped the MCID for TAC (95%CI: -0.42, 0.23). Downgraded.

az. Serious indirectness since only 1 trial was available. Downgraded.

ba. Optimal information size did not meet. The effect size (WMD: -2.60) did not surpass the minimal clinically important difference for GSH (MCID: -37.8 μmol/l). Downgraded.

bb. Not serious inconsistency since I^2^ = 40.4%, Phet = 0.187. Not downgraded.

bc. Serious indirectness since only 2 trials were available. Downgraded.

bd. Optimal information size did not meet. The effect size (WMD: -0.27) did not surpass the minimal clinically important difference for MDA (MCID: -0.59 μmol/l). Downgraded.

be. Serious indirectness since only 1 trial was available. Downgraded.

bf. Optimal information size did not meet. The effect size (WMD: -1.20) did not surpass the minimal clinically important difference for mf-G (MCID: -2.15). Downgraded.

| **Supplementary Table 8: GRADE evidence table for the effects of prebiotic supplementation in women with PCOS.** | | | | | | | | | | | |
| --- | --- | --- | --- | --- | --- | --- | --- | --- | --- | --- | --- |
| Certainty assessment | | | | | | | No of patients | | Effect | Certainty | Importance |
| No of studies | Design | Risk of bias | Inconsistency | Indirectness | Imprecision | Other  considerations | Treatment group | Control group | WMD (95%CI) |  |  |
| Body weight | | | | | | | | | | | |
| 1 | Randomized trials | not serious | not serious | Serious ^a^ | Serious ^b^ | none | 31 | 31 | MD **2.8 kg lower** (6.83 lower to 1.23 higher) | ⨁⨁◯◯ Low | IMPORTANT |
| BMI | | | | | | | | | | | |
| 1 | Randomized trials | not serious | not serious | Serious ^c^ | Serious ^d^ | none | 31 | 31 | MD **1.4 kg/m2 lower** (2.95 lower to 0.15 higher) | ⨁⨁◯◯ Low | IMPORTANT |
| Waist circumference | | | | | | | | | | | |
| 1 | Randomized trials | not serious | not serious | Serious ^e^ | Serious ^f^ | none | 31 | 31 | MD **5.1 cm lower** (8.6 lower to 1.6 lower) | ⨁⨁◯◯ Low | IMPORTANT |
| Hip circumference | | | | | | | | | | | |
| 1 | Randomized trials | not serious | not serious | Serious ^g^ | Serious ^h^ | none | 31 | 31 | MD **4.6 cm lower** (7.47 lower to 1.73 lower) | ⨁⨁◯◯ Low | IMPORTANT |
| Fasting glucose concentration | | | | | | | | | | | |
| 1 | Randomized trials | not serious | not serious | Serious ^i^ | Serious ^j^ | none | 31 | 31 | MD **15.14 mg/dl lower** (20.38 lower to 9.9 lower) | ⨁⨁◯◯ Low | IMPORTANT |
| Triglycerides | | | | | | | | | | | |
| 1 | Randomized trials | not serious | not serious | Serious ^k^ | Serious ^l^ | none | 31 | 31 | MD **31.12 mg/dl lower** (49.63 lower to 12.61 lower) | ⨁⨁◯◯ Low | IMPORTANT |
| Total cholesterol | | | | | | | | | | | |
| 1 | Randomized trials | not serious | not serious | Serious ^m^ | Serious ^n^ | none | 31 | 31 | MD **34.83 mg/dl lower** (52.47 lower to 17.19 lower) | ⨁⨁◯◯ Low | IMPORTANT |
| HDL cholesterol | | | | | | | | | | | |
| 1 | Randomized trials | not serious | not serious | Serious ^o^ | Serious ^p^ | none | 31 | 31 | MD **37.65 mg/dl lower** (52.09 lower to 22.69 lower) | ⨁⨁◯◯ Low | IMPORTANT |
| LDL cholesterol | | | | | | | | | | | |
| 1 | Randomized trials | not serious | not serious | Serious ^q^ | Serious ^r^ | none | 31 | 31 | MD **7.2 mg/dl higher** (1.63 lower to 11.4 higher) | ⨁⨁◯◯ Low | IMPORTANT |
| Dehydroepiandrosterone sulfate | | | | | | | | | | | |
| 1 | Randomized trials | not serious | not serious | Serious ^s^ | Serious ^t^ | none | 31 | 31 | MD **0.84 μg/ml lower** (1.52 lower to 0.16 lower) | ⨁⨁◯◯ Low | IMPORTANT |
| high-sensitivity C-reactive protein | | | | | | | | | | | |
| 1 | Randomized trials | not serious | not serious | Serious ^u^ | Serious ^v^ | none | 31 | 31 | MD **1.94 mg/l lower** (3.27 lower to 0.61 lower) | ⨁⨁◯◯ Low | IMPORTANT |
| Hirsutism score | | | | | | | | | | | |
| 1 | Randomized trials | not serious | not serious | Serious ^w^ | Serious ^x^ | none | 31 | 31 | MD **1.68 lower** (3.19 lower to 0.17 lower) | ⨁⨁◯◯ Low | IMPORTANT |

CI: confidence interval; MD: mean difference

Explanations

a. Serious indirectness since only 1 trial was available. Downgraded.

b. Optimal information size did not meet. The effect size (WMD: -2.80) surpassed the minimal clinically important difference for body weight (MCID: -2.5 kg), but the upper bound of the 95%CI overlapped the MCID for body weight (95%CI: -6.83, 1.23). Downgraded.

c. Serious indirectness since only 1 trial was available. Downgraded.

d. Optimal information size did not meet. The effect size (WMD: -1.40) surpassed the minimal clinically important difference for BMI (MCID: -0.95 kg/m2), but the upper bound of the 95%CI overlapped the MCID for BMI (95%CI: -2.95, 0.15). Downgraded.

e. Serious indirectness since only 1 trial was available. Downgraded.

f. Optimal information size did not meet. The effect size (WMD: -5.10) surpassed the minimal clinically important difference for WC (MCID: -2 cm), but the upper bound of the 95%CI overlapped the MCID for WC (95%CI: -8.60, -1.60). Downgraded.

g. Serious indirectness since only 1 trial was available. Downgraded.

h. Optimal information size did not meet. The effect size (WMD: -4.60) surpassed the minimal clinically important difference for hip circumference (MCID: -3.60 cm), but the upper bound of the 95%CI overlapped the MCID for hip circumference (95%CI: -7.47, -1.73). Downgraded.

i. Serious indirectness since only 1 trial was available. Downgraded.

j. Optimal information size did not meet. The effect size (WMD:- 15.14) did not surpass the minimal clinically important difference for FBS (MCID: -28.5 mg/dL). Downgraded.

k. Serious indirectness since only 1 trial was available. Downgraded.

l. Optimal information size did not meet. The effect size (WMD: -31.12) surpassed the minimal clinically important difference for TG (MCID: -8 mg/dl), and the lower and upper bound of the 95%CI did not overlap the MCID for TG (95%CI: -49.63, -12.61). Downgraded.

m. Serious indirectness since only 1 trial was available. Downgraded.

n. Optimal information size did not meet. The effect size (WMD: -34.83) surpassed the minimal clinically important difference for TC (MCID: -10 mg/dl), and the lower and upper bound of the 95%CI did not overlap the MCID for TC (95%CI: -52.47, -17.19). Downgraded.

o. Serious indirectness since only 1 trial was available. Downgraded.

p. Optimal information size did not meet. The effect size (WMD: -37.65) surpassed the minimal clinically important difference for HDL cholesterol (MCID: -3.87 mg/dl), and the lower and upper bound of the 95%CI did not overlap the MCID for HDL cholesterol (95%CI: -52.09, -22.69). Downgraded.

q. Serious indirectness since only 1 trial was available. Downgraded.

r. Optimal information size did not meet. The effect size (WMD: 7.20) surpassed the minimal clinically important difference for LDL cholesterol (MCID: 3.87 mg/dl), but the lower bound of the 95%CI overlapped the MCID for LDL cholesterol (95%CI: -1.63, 11.40). Downgraded.

s. Serious indirectness since only 1 trial was available. Downgraded.

t. Optimal information size did not meet. The effect size (WMD: -0.84) surpassed the minimal clinically important difference for DHEAS (MCID: -0.6 μg/mL), but the upper bound of the 95%CI overlapped the MCID for DHEAS (95%CI: -1.52, -0.16). Downgraded.

u. Serious indirectness since only 1 trial was available. Downgraded.

v. Optimal information size did not meet. The effect size (WMD: -1.94) surpassed the minimal clinically important difference for hs-CRP (MCID: -0.5 mg/l), and the lower and upper bound of the 95%CI did not overlap the MCID for hs-CRP (95%CI: -3.27, -0.61). Downgraded.

w. Serious indirectness since only 1 trial was available. Downgraded.

x. Optimal information size did not meet. The effect size (WMD: -1.68) did not surpass the minimal clinically important difference for mf-G (MCID: -2.15). Downgraded.

**Supplementary Figure 1.** Funnel plot for evaluation publication bias in the studies reporting the effect of probiotic supplementation on body weight.

**
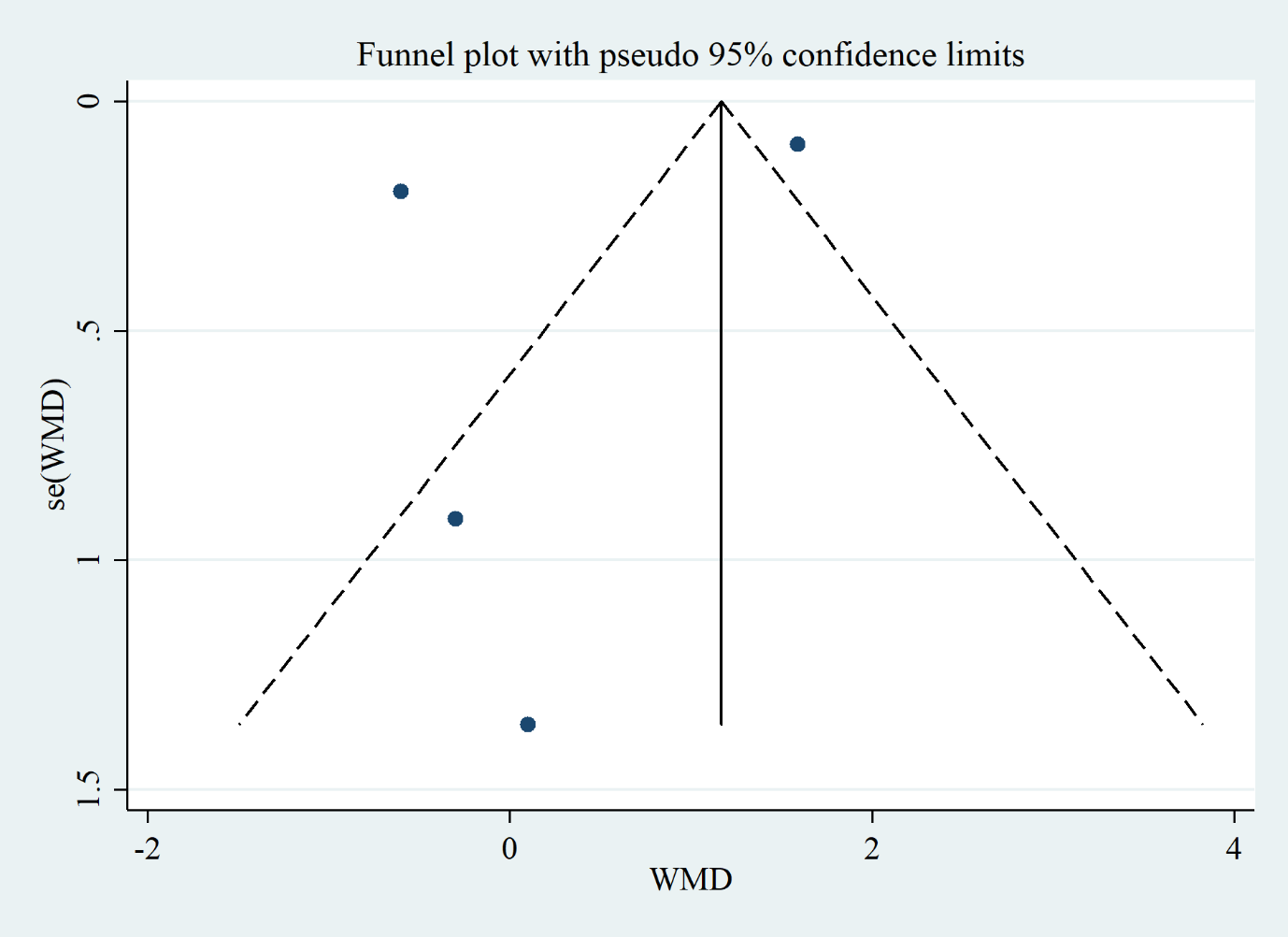
**

**Supplementary Figure 2.** Funnel plot for evaluation publication bias in the studies reporting the effect of probiotic supplementation on BMI.

**
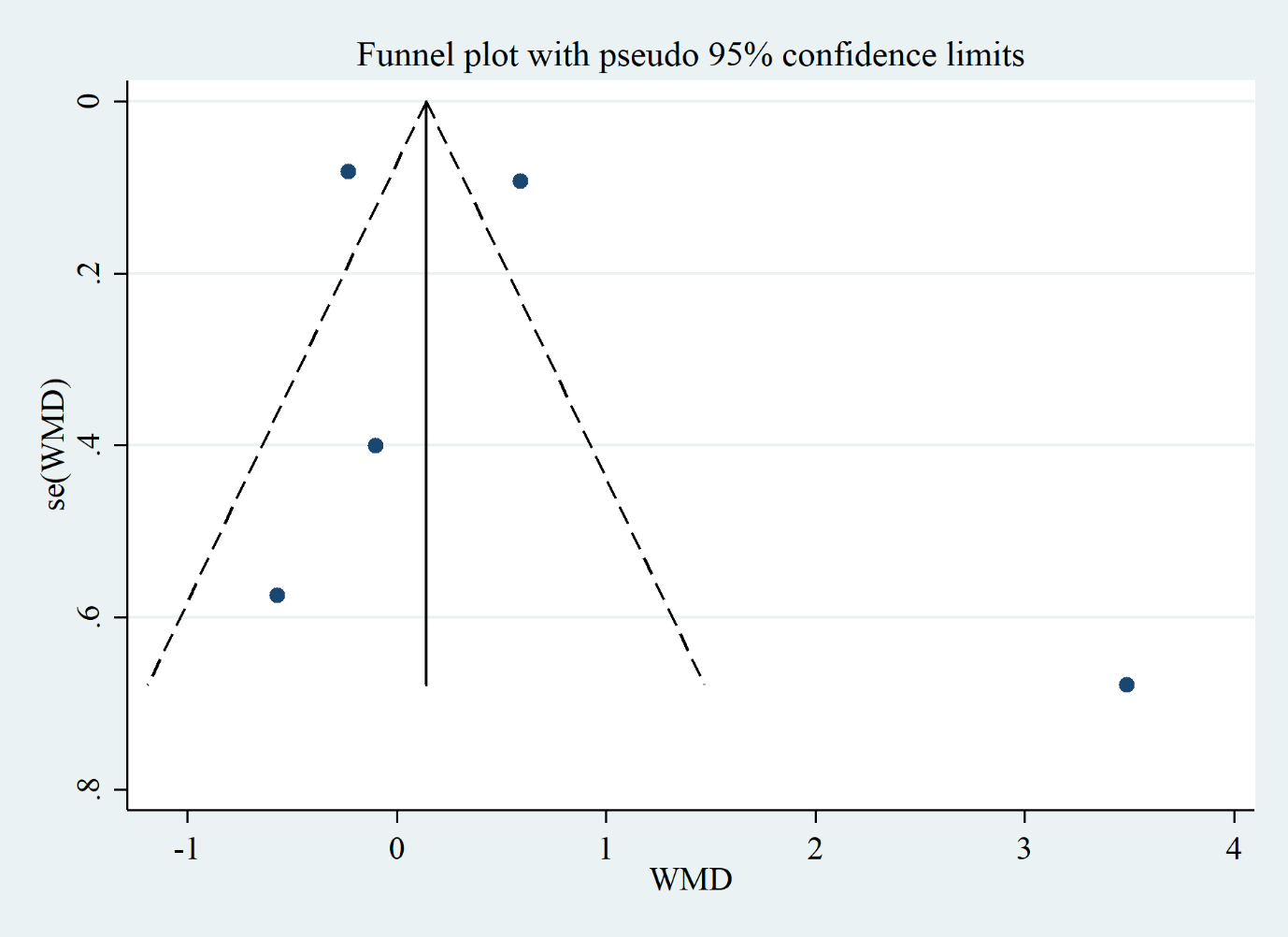
**

**Supplementary Figure 3.** Funnel plot for evaluation publication bias in the studies reporting the effect of probiotic supplementation on waist circumference. **
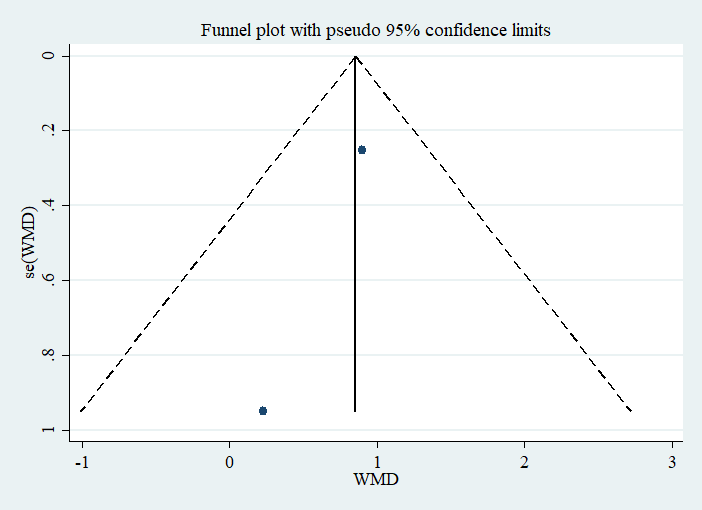
**

**Supplementary Figure 4.** Funnel plot for evaluation publication bias in the studies reporting the effect of probiotic supplementation on Fasting glucose concentration. **
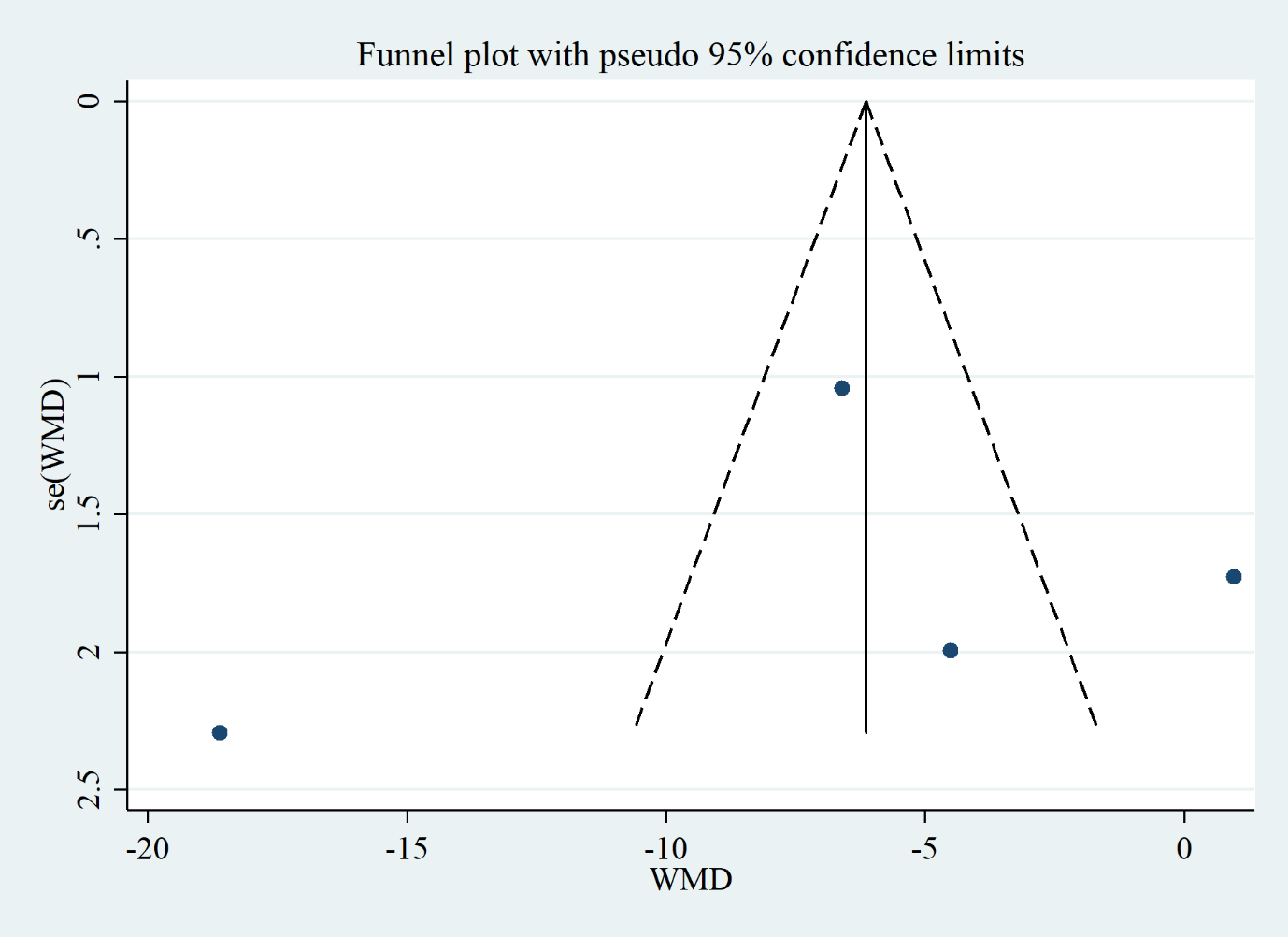
**

**Supplementary Figure 5.** Funnel plot for evaluation publication bias in the studies reporting the effect of probiotic supplementation on Fasting insulin. **
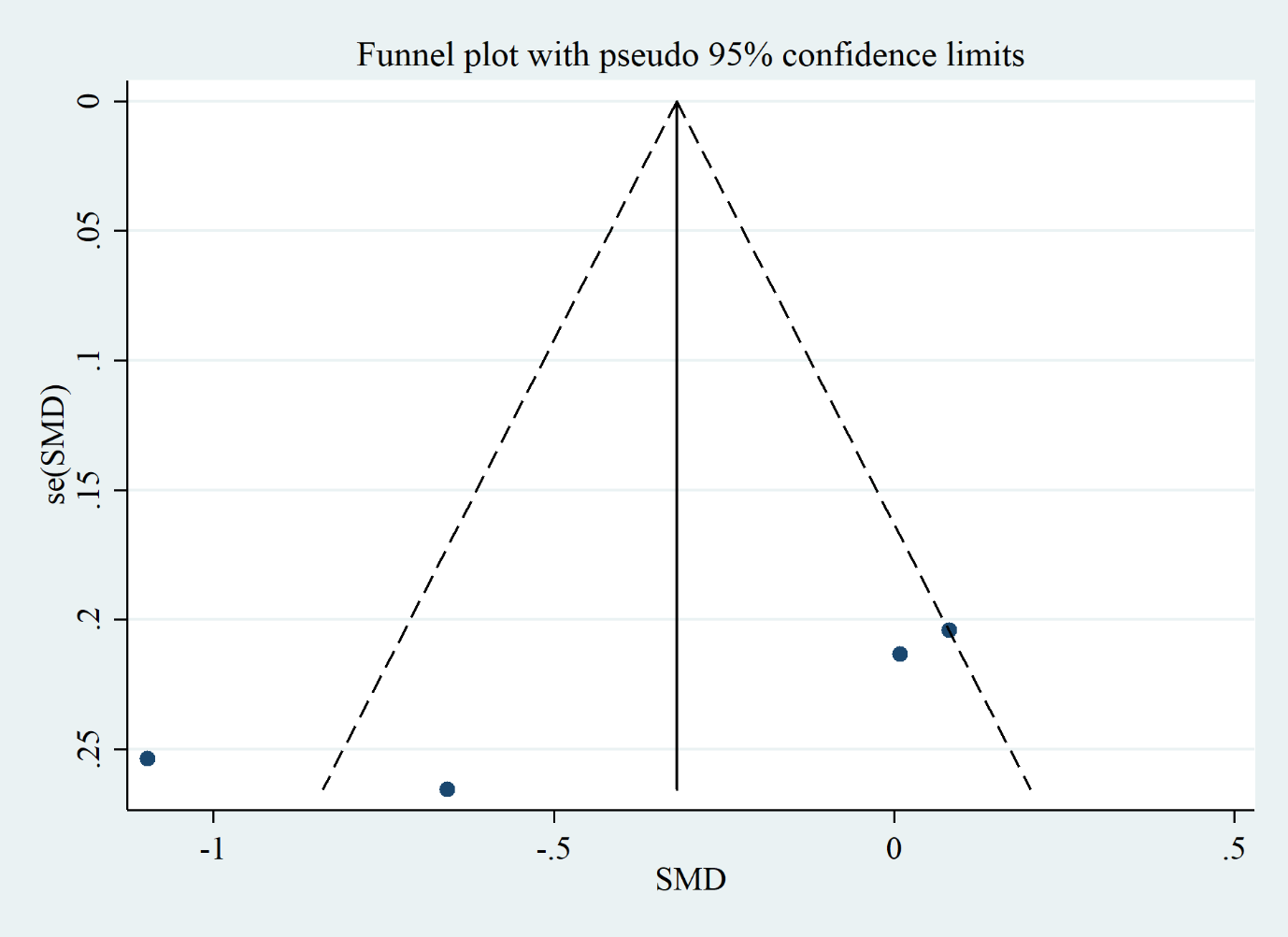
**

**Supplementary Figure 6.** Funnel plot for evaluation publication bias in the studies reporting the effect of probiotic supplementation on HOMA-IR. **
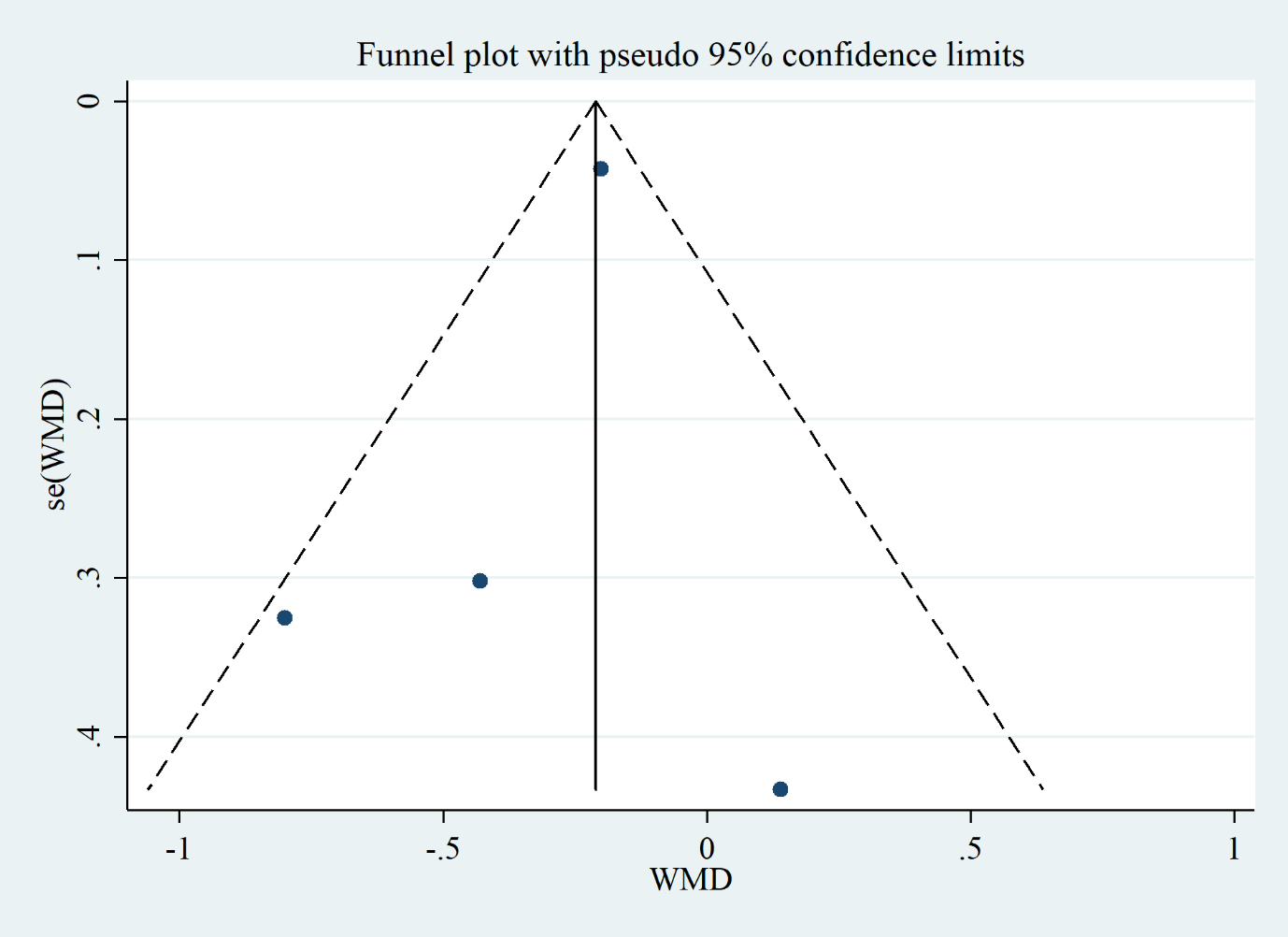
**

**Supplementary Figure 7.** Funnel plot for evaluation publication bias in the studies reporting the effect of probiotic supplementation on QUICKI. **
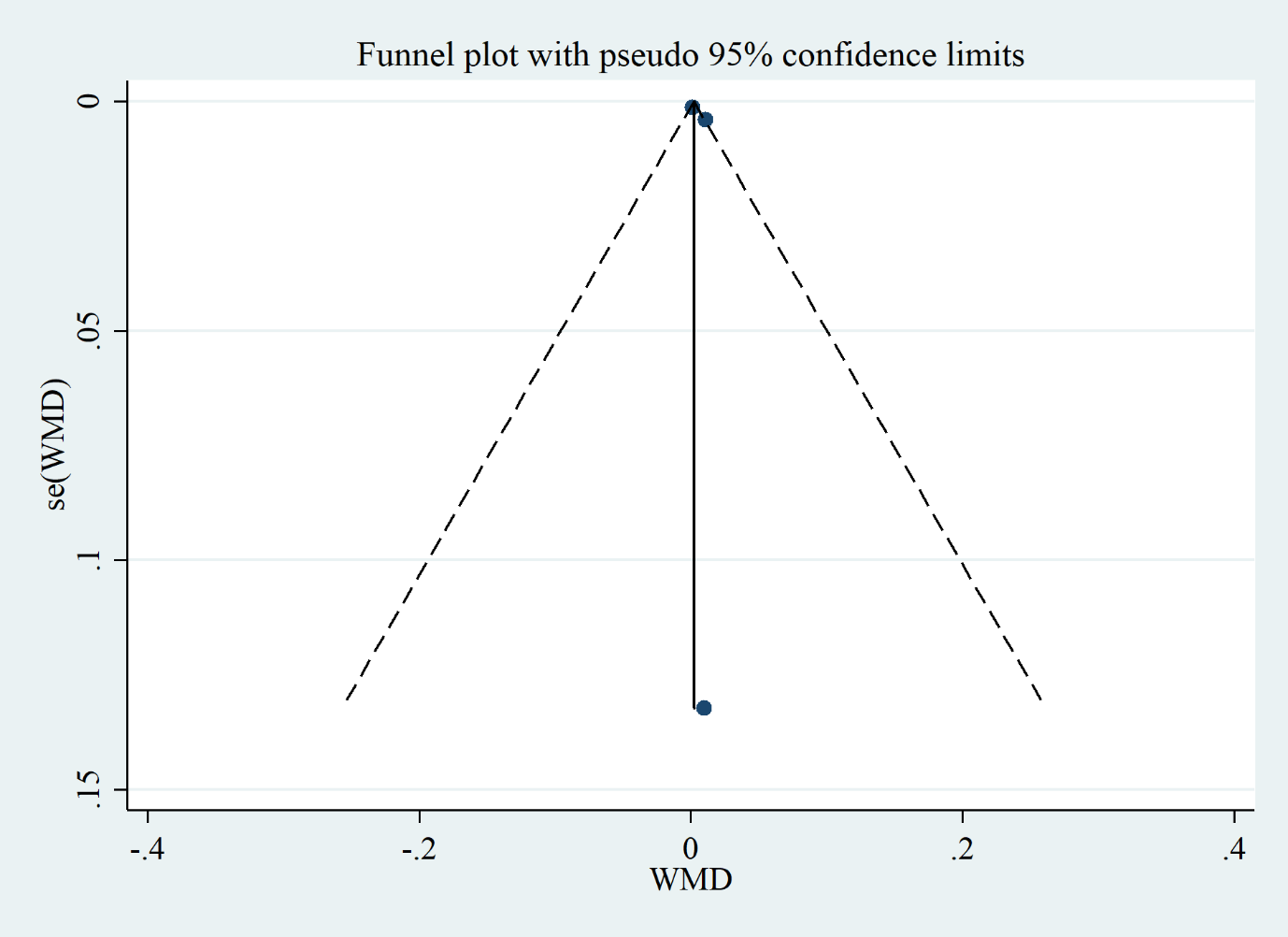
**

**Supplementary Figure 8.** Funnel plot for evaluation publication bias in the studies reporting the effect of probiotic supplementation on triglycerides. **
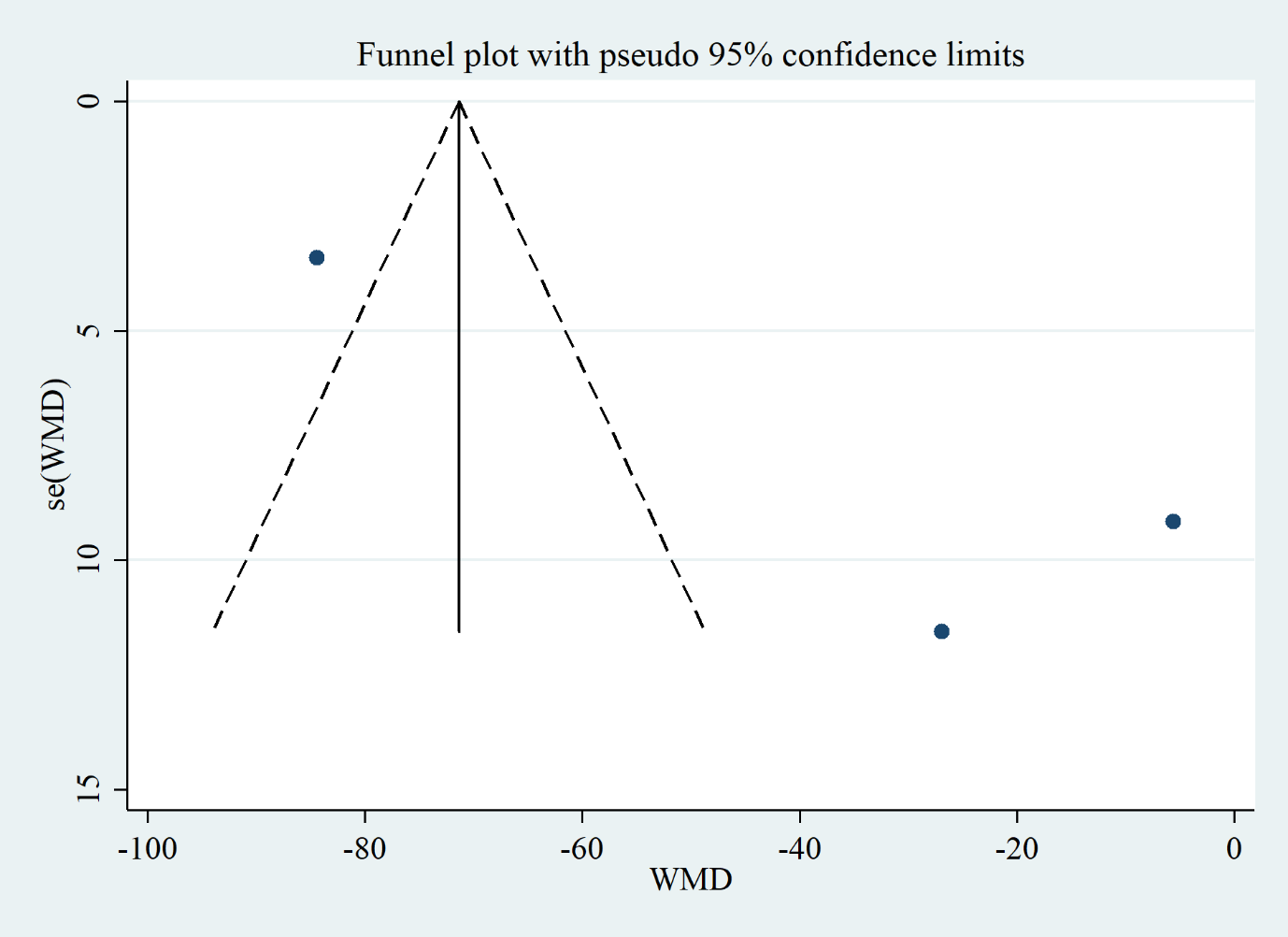
**

**Supplementary Figure 9.** Funnel plot for evaluation publication bias in the studies reporting the effect of probiotic supplementation on total cholesterol. **
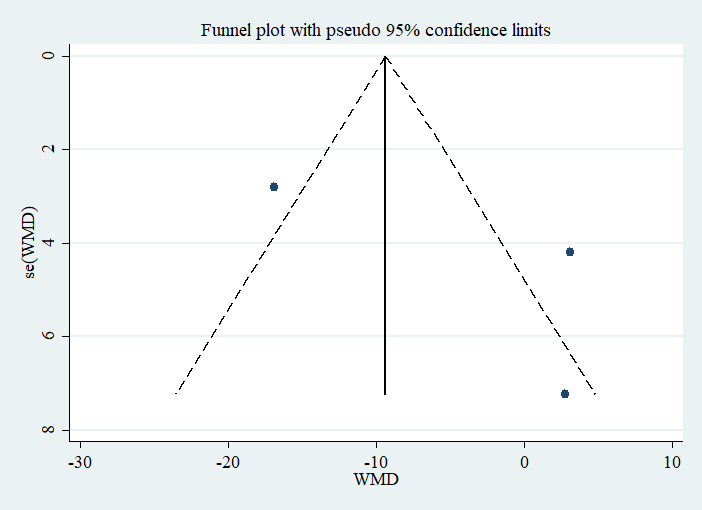
**

**Supplementary Figure 10.** Funnel plot for evaluation publication bias in the studies reporting the effect of probiotic supplementation on LDL cholesterol. **
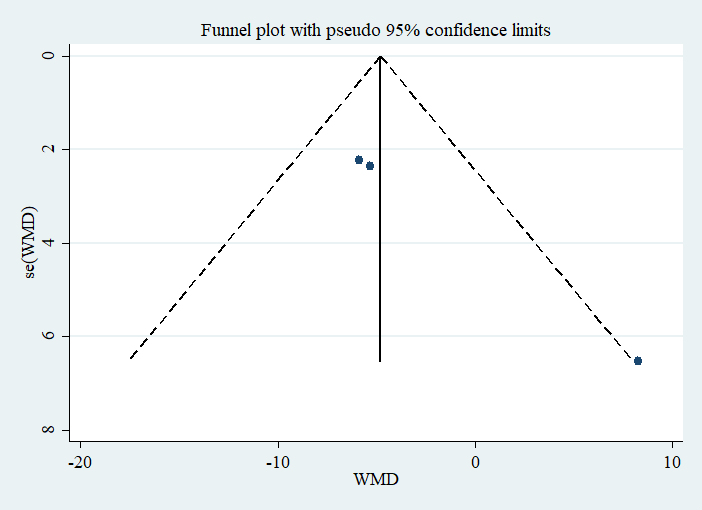
**

**Supplementary Figure 11.** Funnel plot for evaluation publication bias in the studies reporting the effect of probiotic supplementation on HDL cholesterol. **
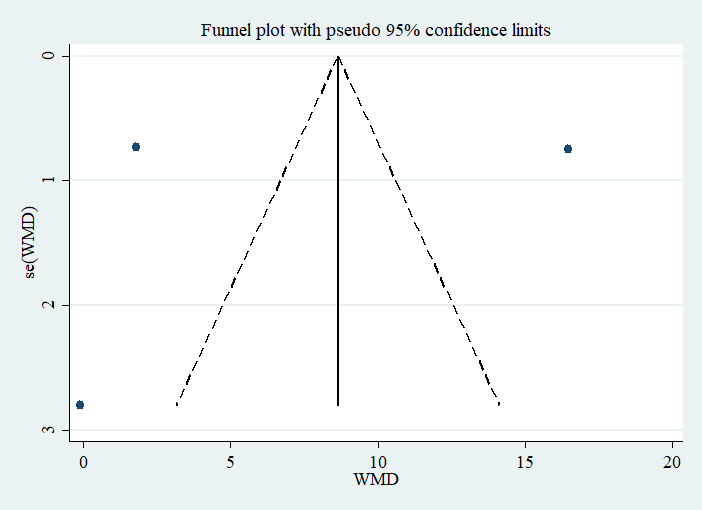
**

**Supplementary Figure 12.** Funnel plot for evaluation publication bias in the studies reporting the effect of probiotic supplementation on high-sensitivity C-reactive protein. **
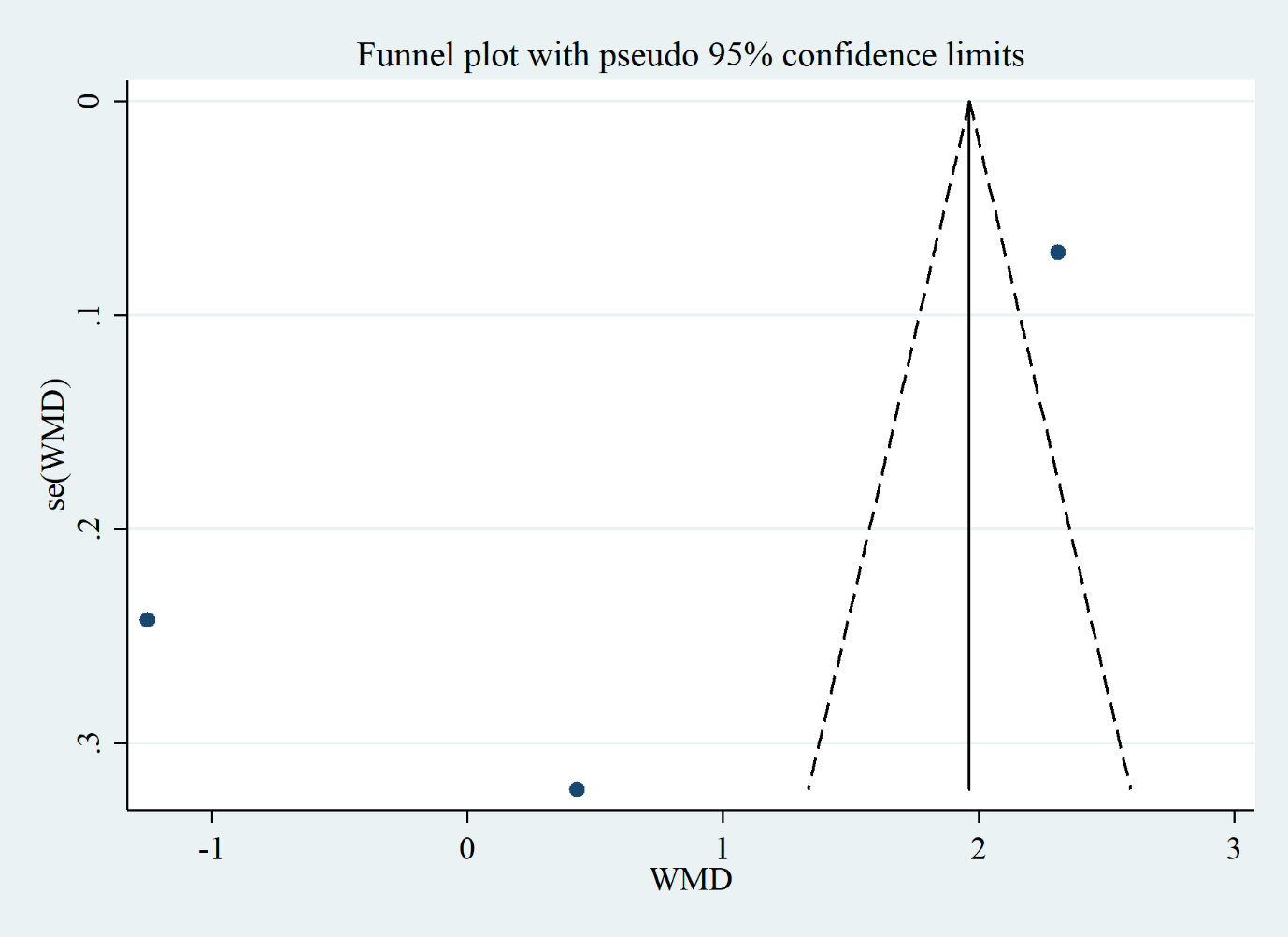
**

**Supplementary Figure 13.** Funnel plot for evaluation publication bias in the studies reporting the effect of probiotic supplementation on body weight. **
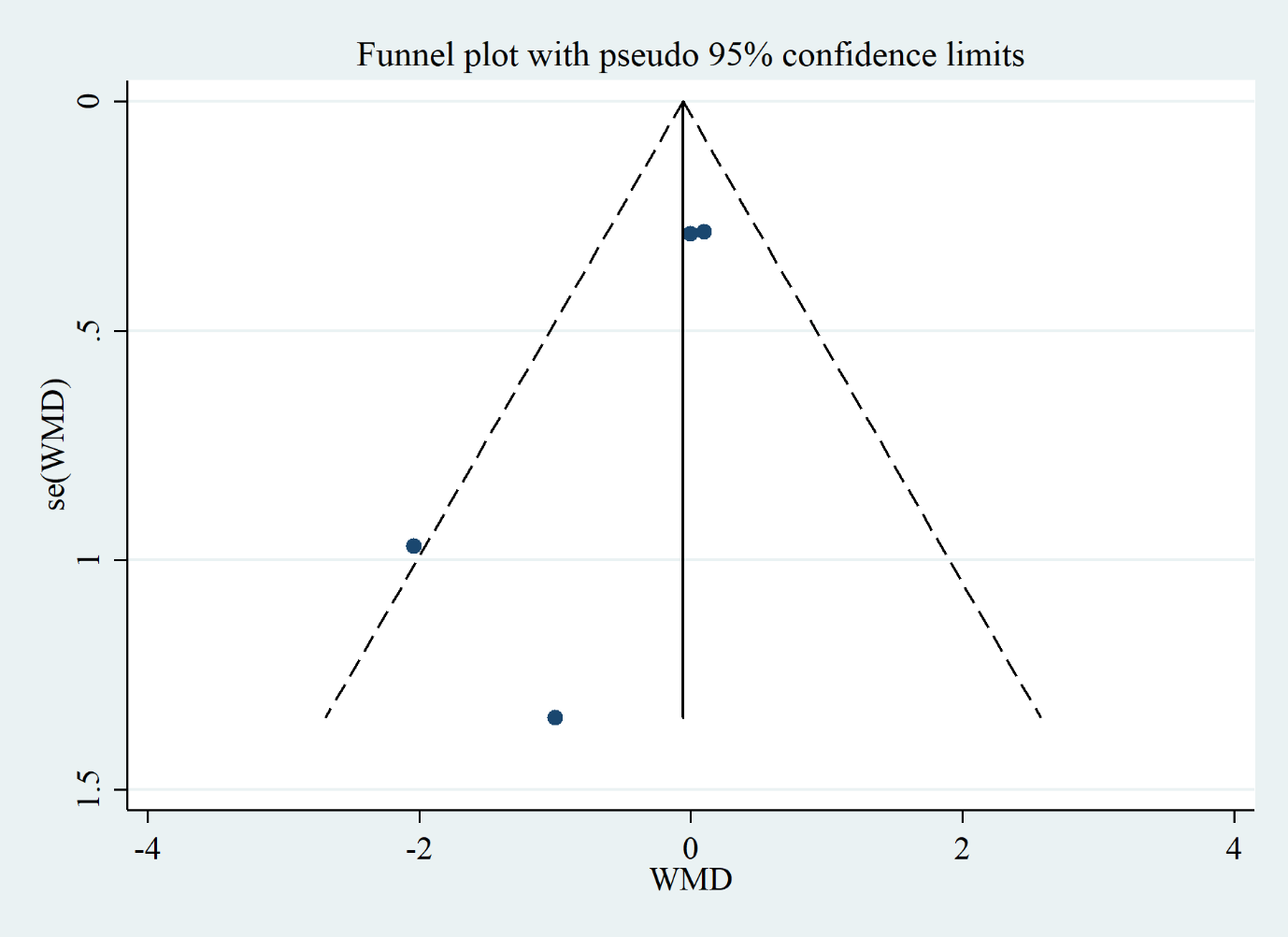
**

**Supplementary Figure 14.** Funnel plot for evaluation publication bias in the studies reporting the effect of probiotic supplementation on BMI. **
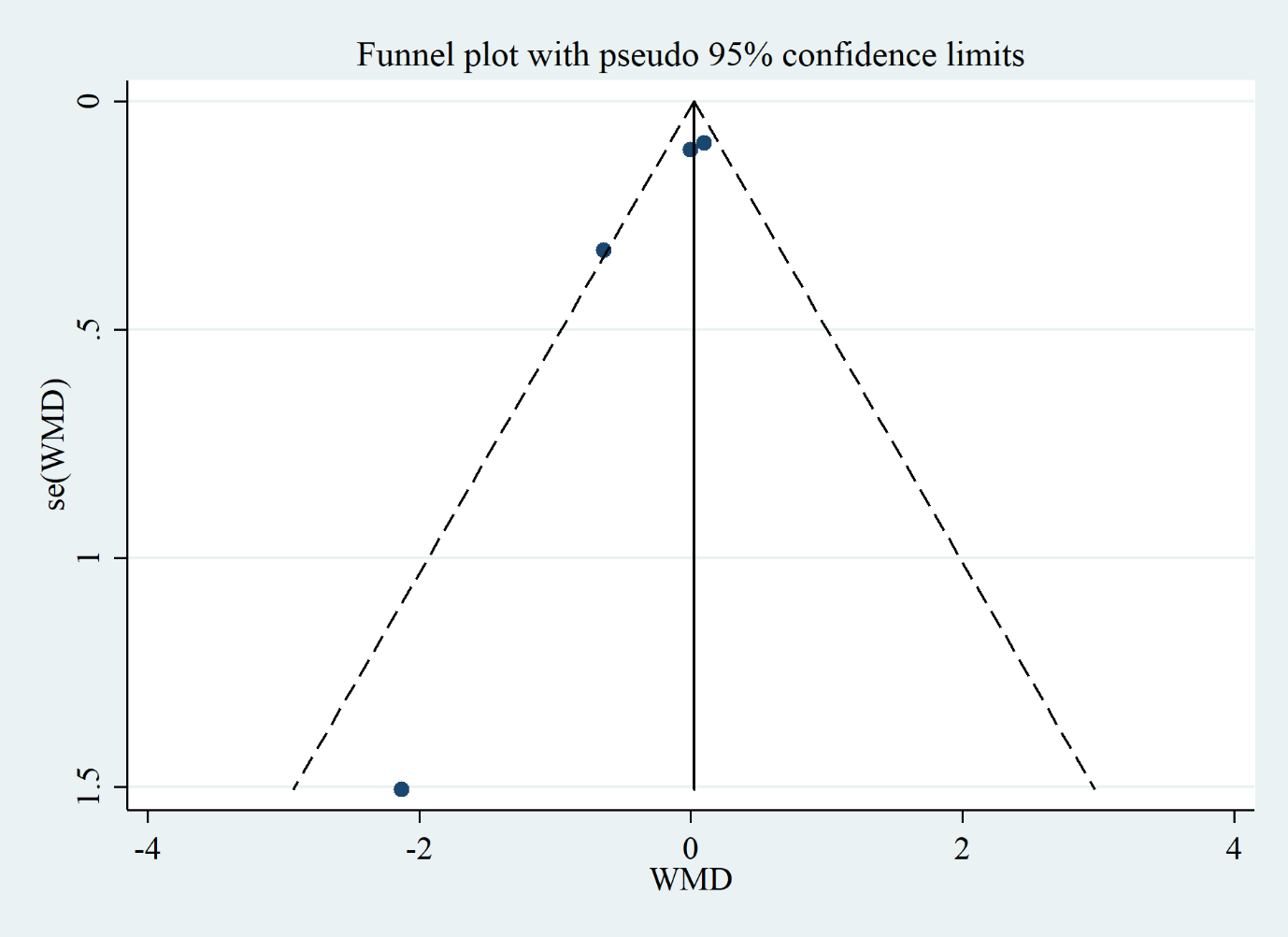
**

**Supplementary Figure 15.** Funnel plot for evaluation publication bias in the studies reporting the effect of probiotic supplementation on Fasting glucose concentration. **
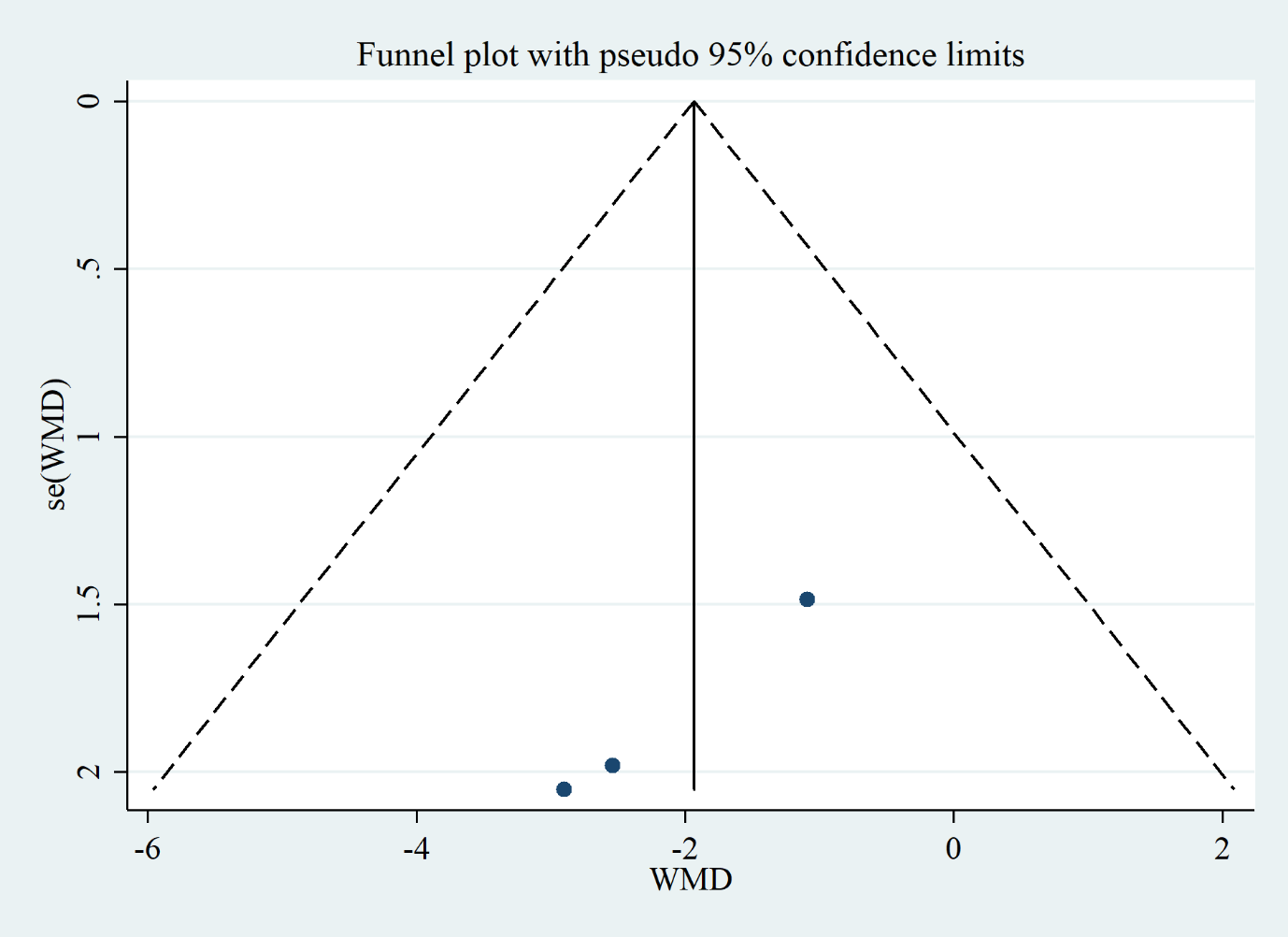
**

**Supplementary Figure 16.** Funnel plot for evaluation publication bias in the studies reporting the effect of probiotic supplementation on Fasting insulin. **
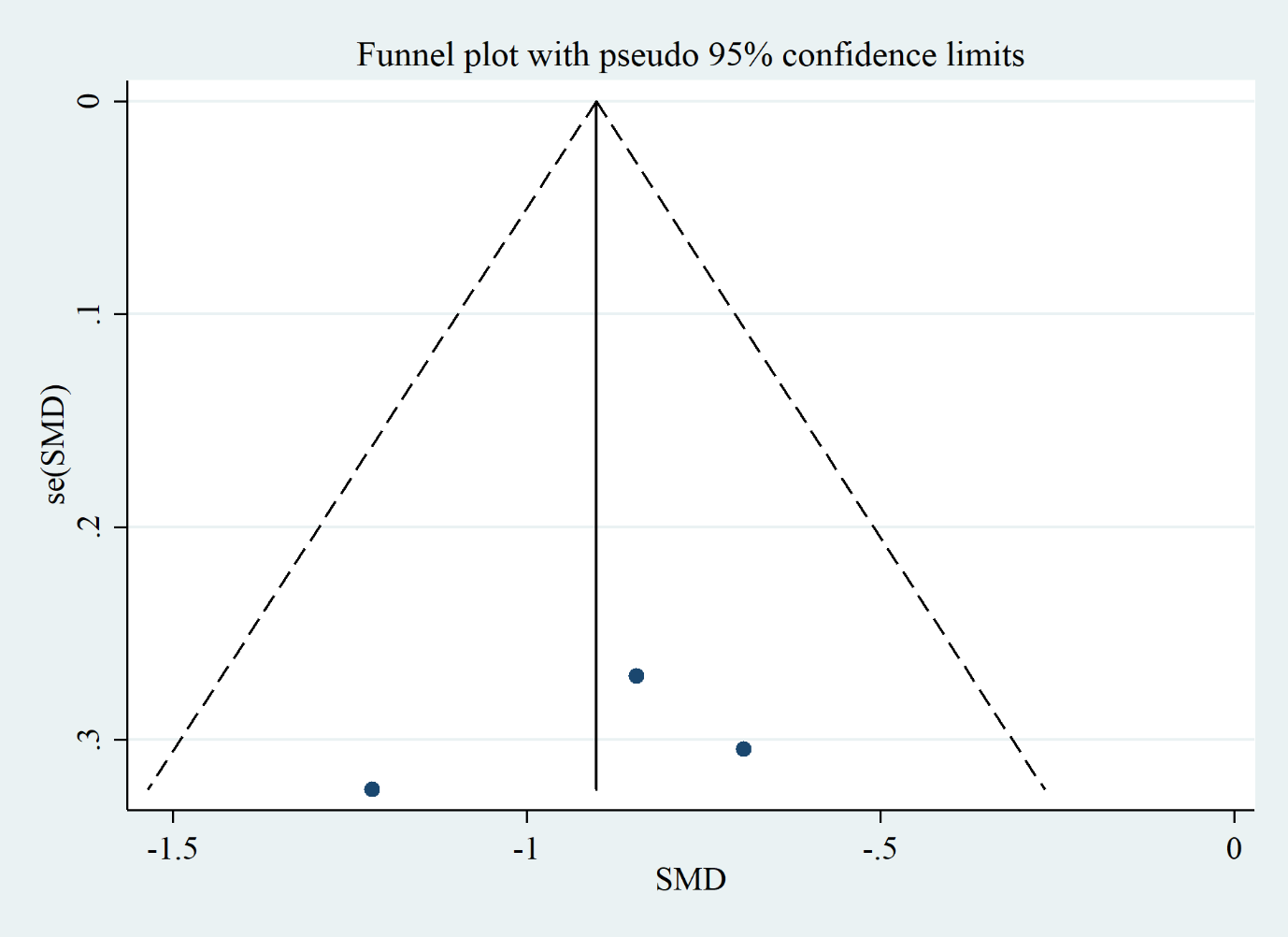
**

**Supplementary Figure 17.** Funnel plot for evaluation publication bias in the studies reporting the effect of probiotic supplementation on HOMA-IR. **
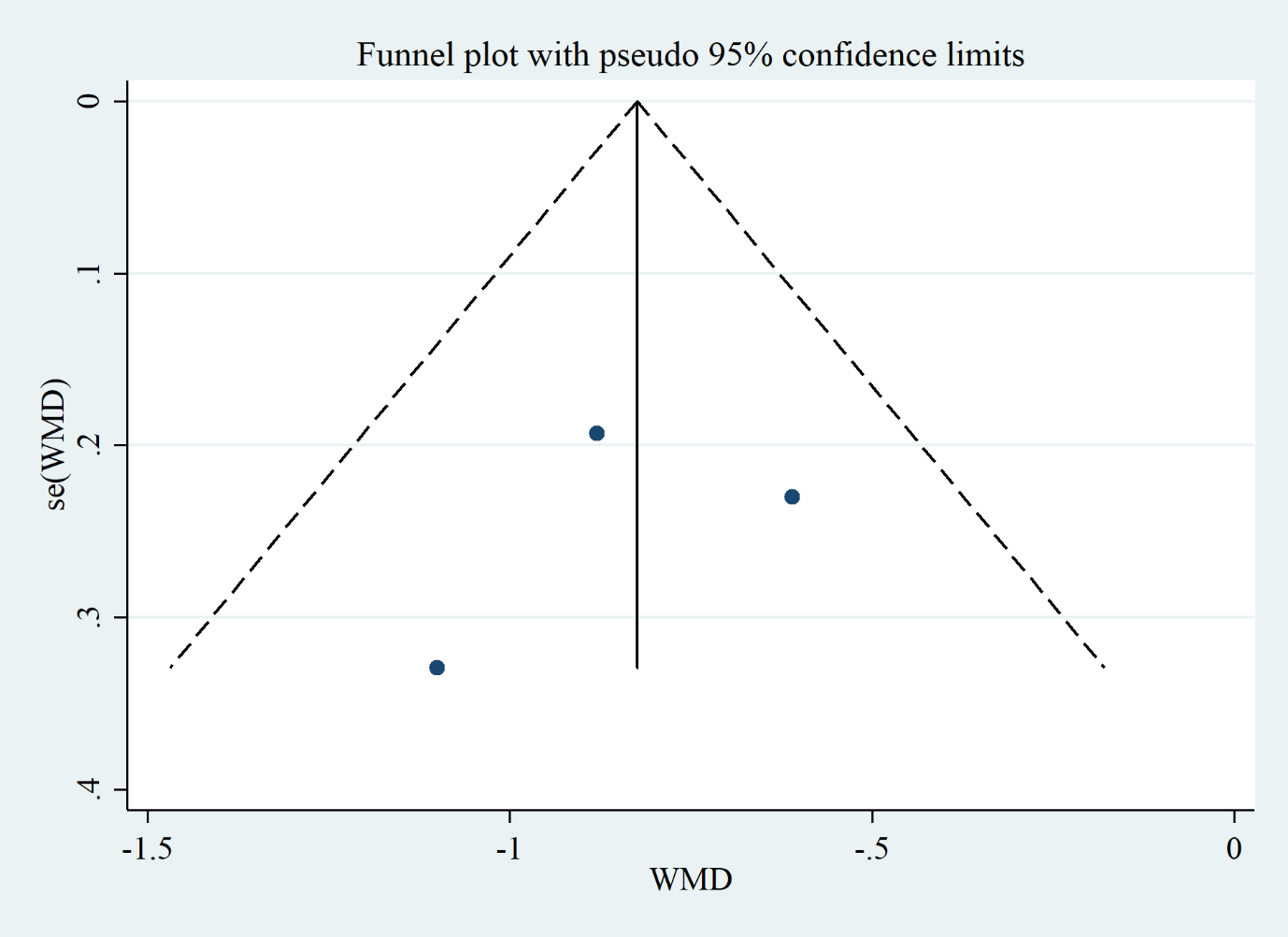
**

**Supplementary Figure 18.** Funnel plot for evaluation publication bias in the studies reporting the effect of probiotic supplementation on QUICKI. **
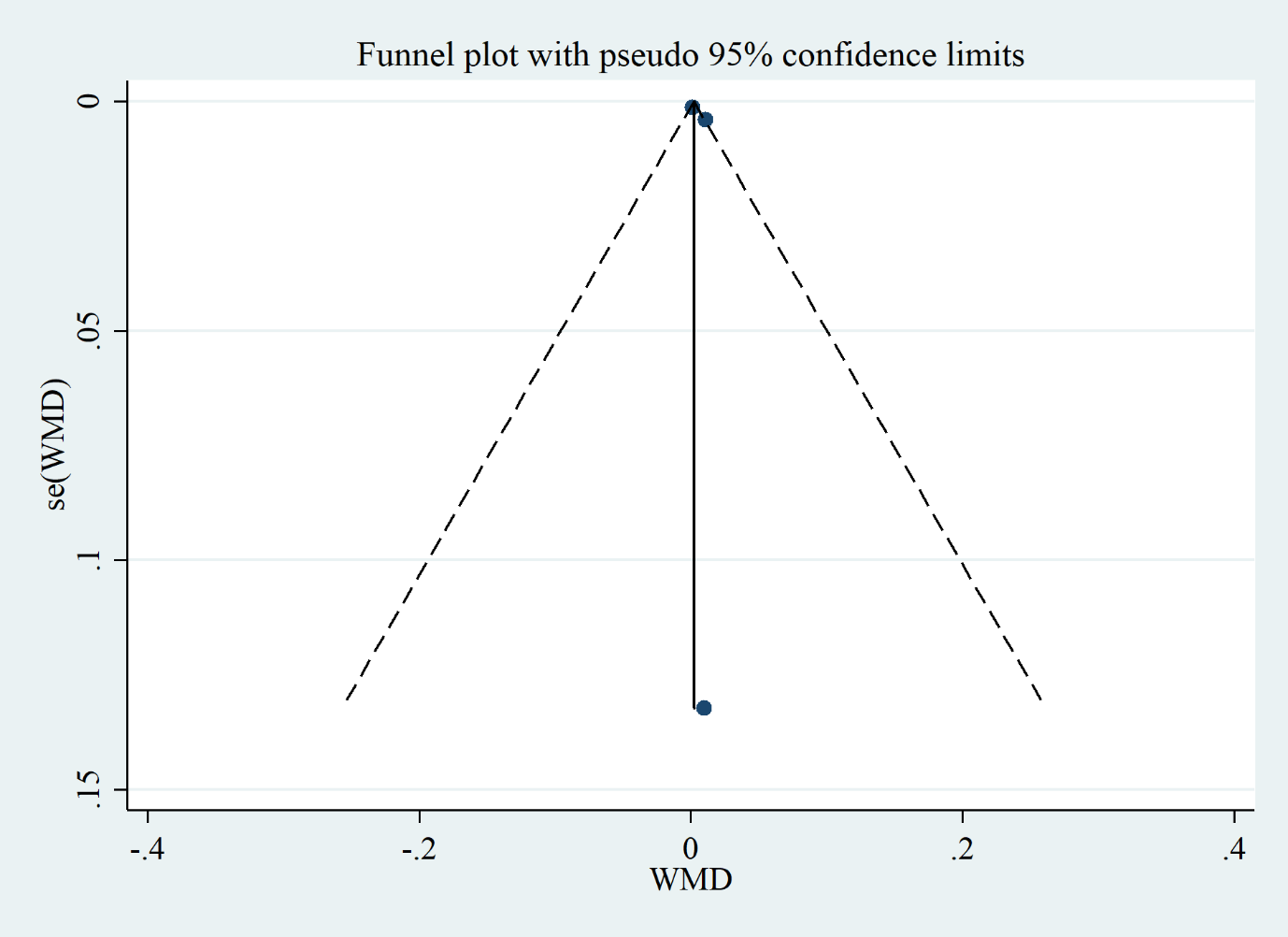
**

**Supplementary Figure 19.** Funnel plot for evaluation publication bias in the studies reporting the effect of probiotic supplementation on Triglycerides. **
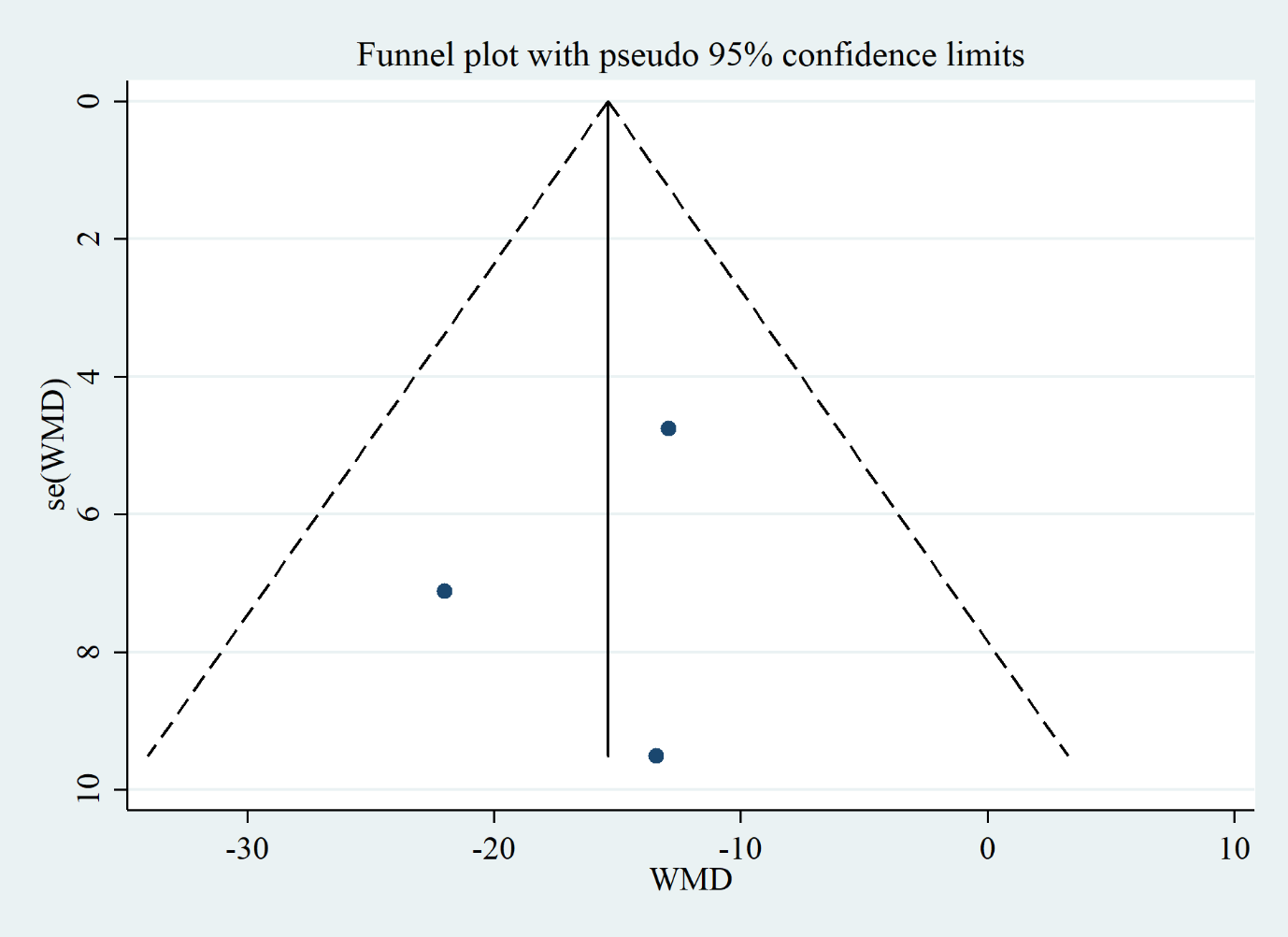
**

**Supplementary Figure 20.** Funnel plot for evaluation publication bias in the studies reporting the effect of probiotic supplementation on Total cholesterol. **
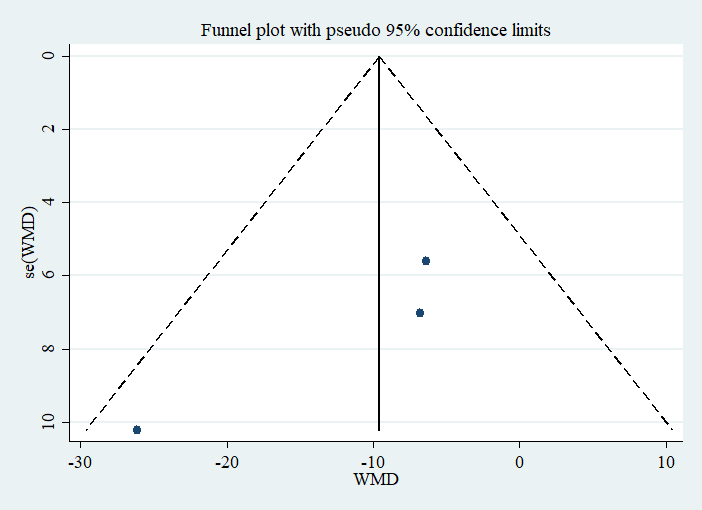
**

**Supplementary Figure 21.** Funnel plot for evaluation publication bias in the studies reporting the effect of probiotic supplementation on LDL cholesterol. **
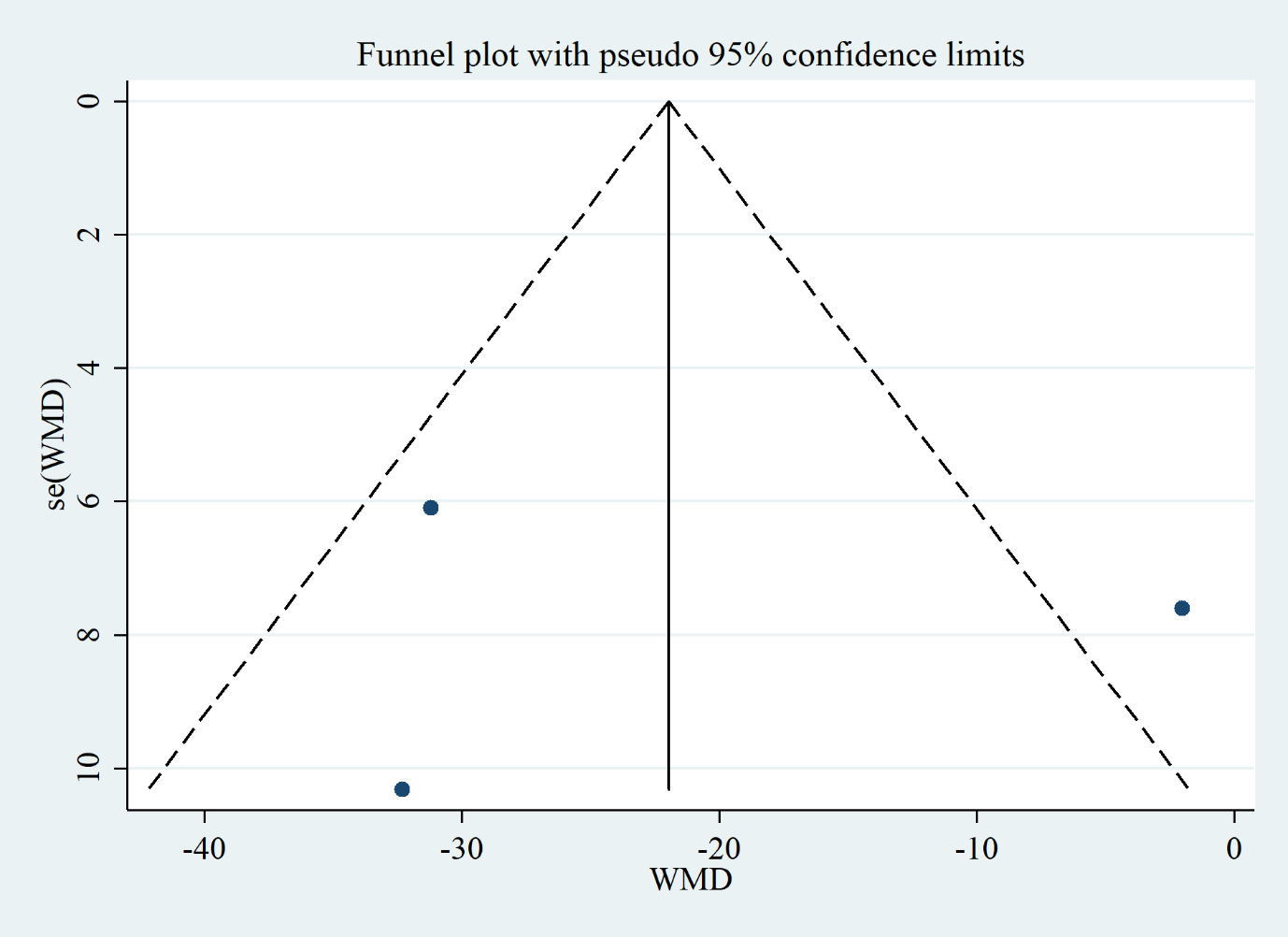
**

**Supplementary Figure 22.** Funnel plot for evaluation publication bias in the studies reporting the effect of probiotic supplementation on HDL cholesterol. **
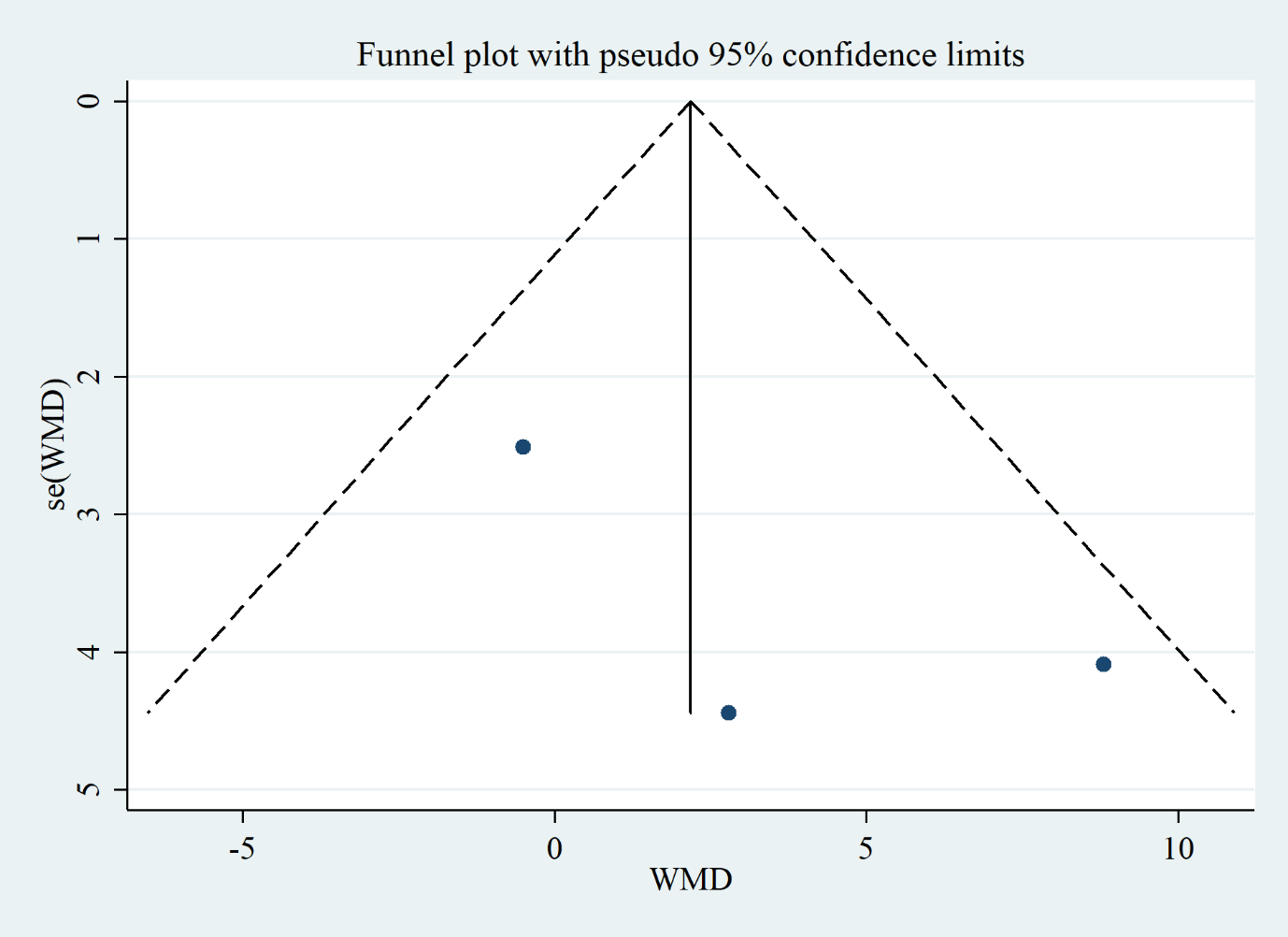
**

**Supplementary Figure 23.** Funnel plot for evaluation publication bias in the studies reporting the effect of probiotic supplementation on Total testosterone. **
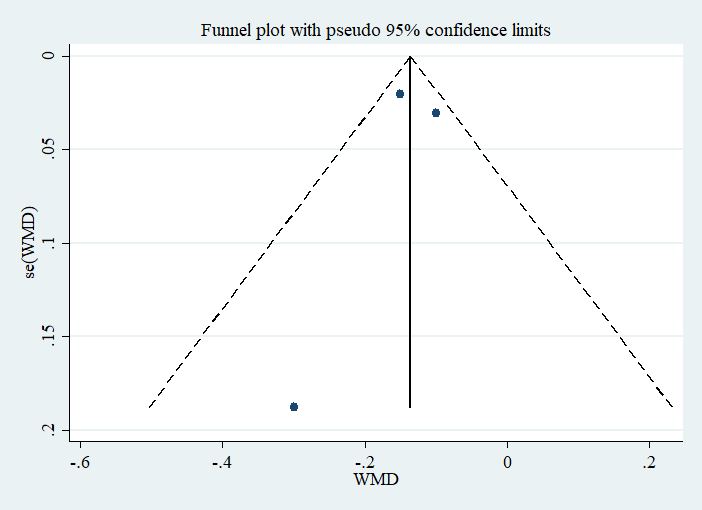
**

**Supplementary Figure 24.** Funnel plot for evaluation publication bias in the studies reporting the effect of probiotic supplementation on high-sensitivity C-reactive protein. **
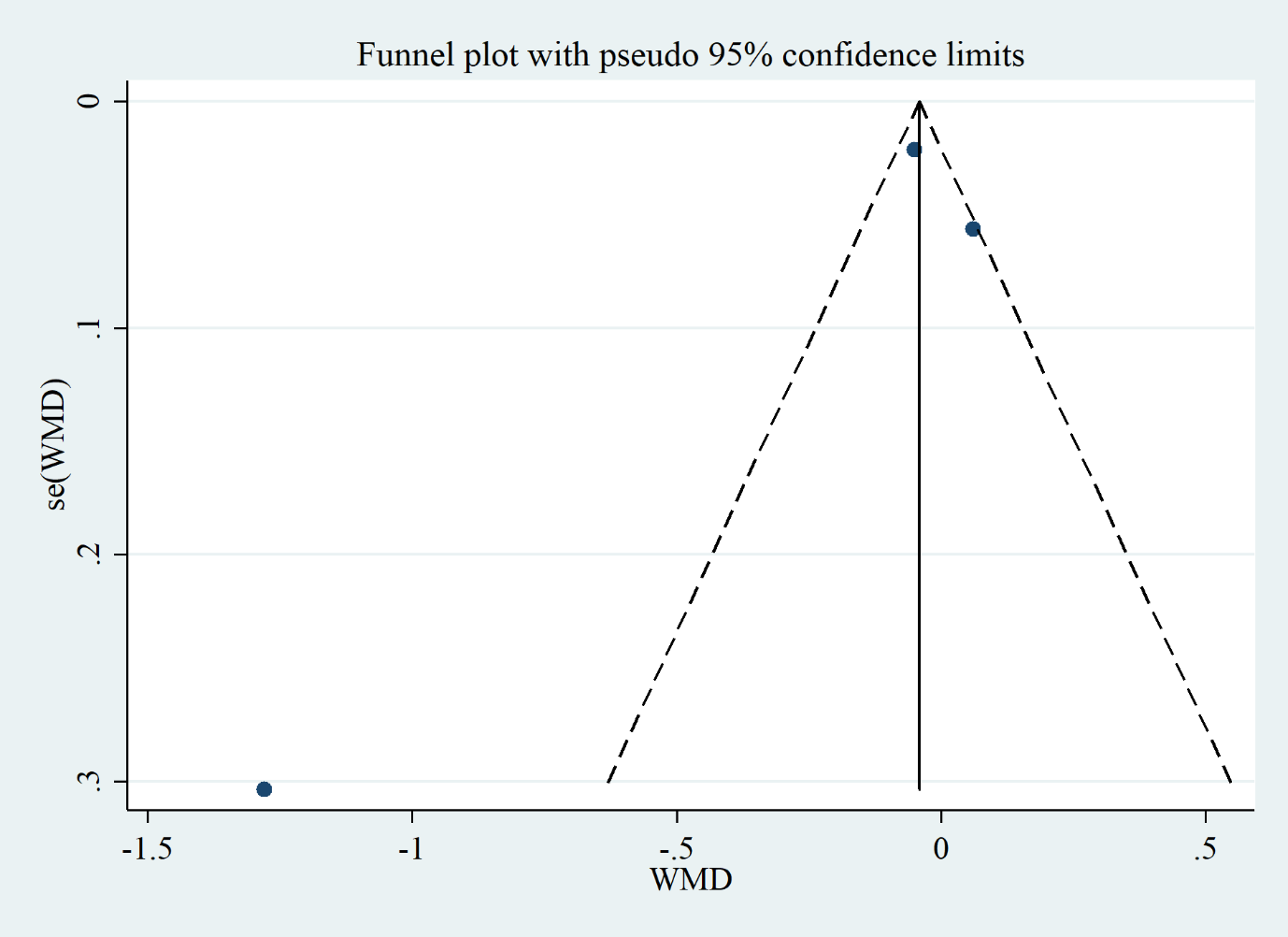
**

**Supplementary Figure 25.** Funnel plot for evaluation publication bias in the studies reporting the effect of probiotic supplementation on Total antioxidant capacity. **
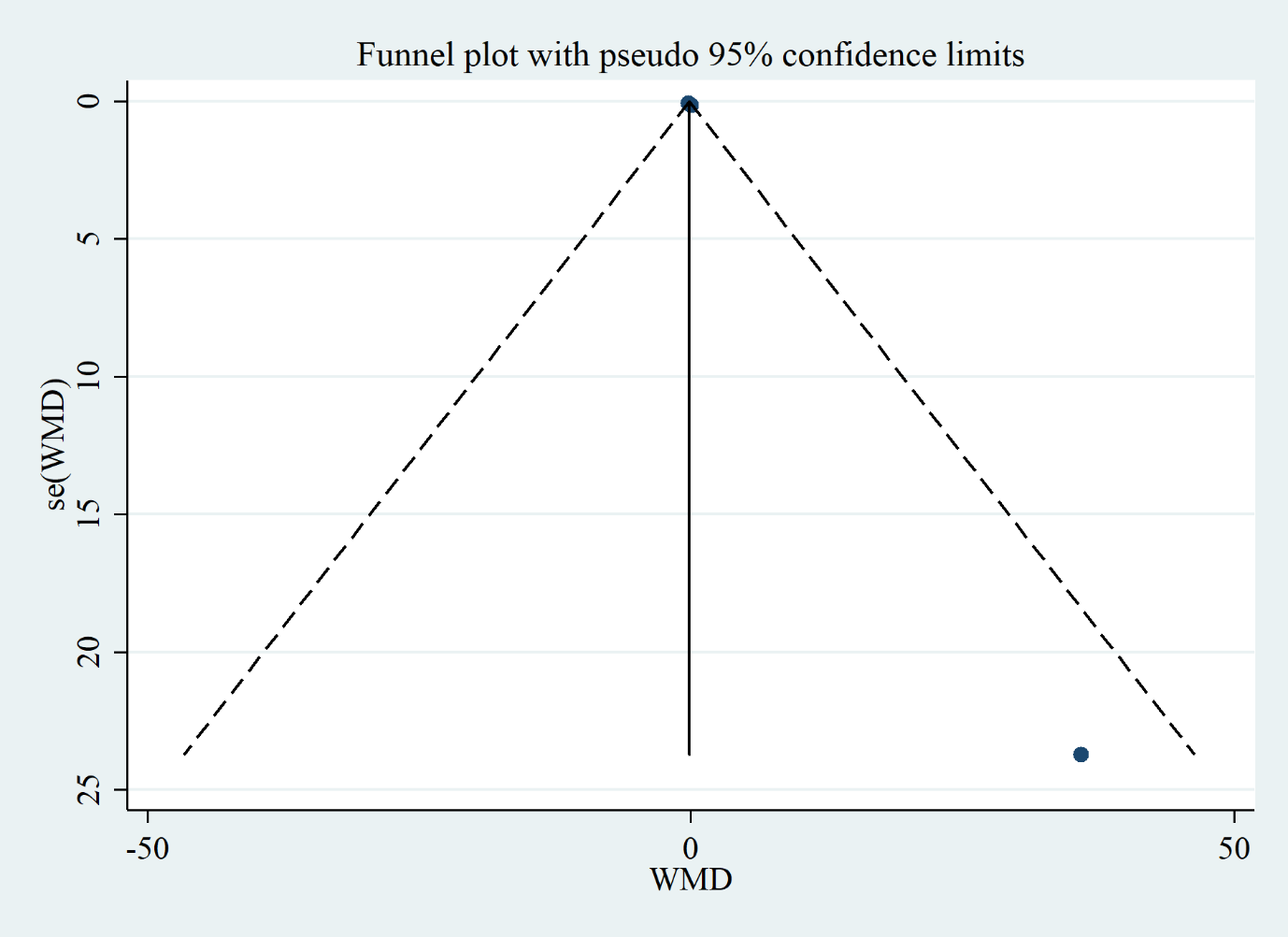
**

**Supplementary Figure 26.** Funnel plot for evaluation publication bias in the studies reporting the effect of probiotic supplementation on malondialdehyde. **
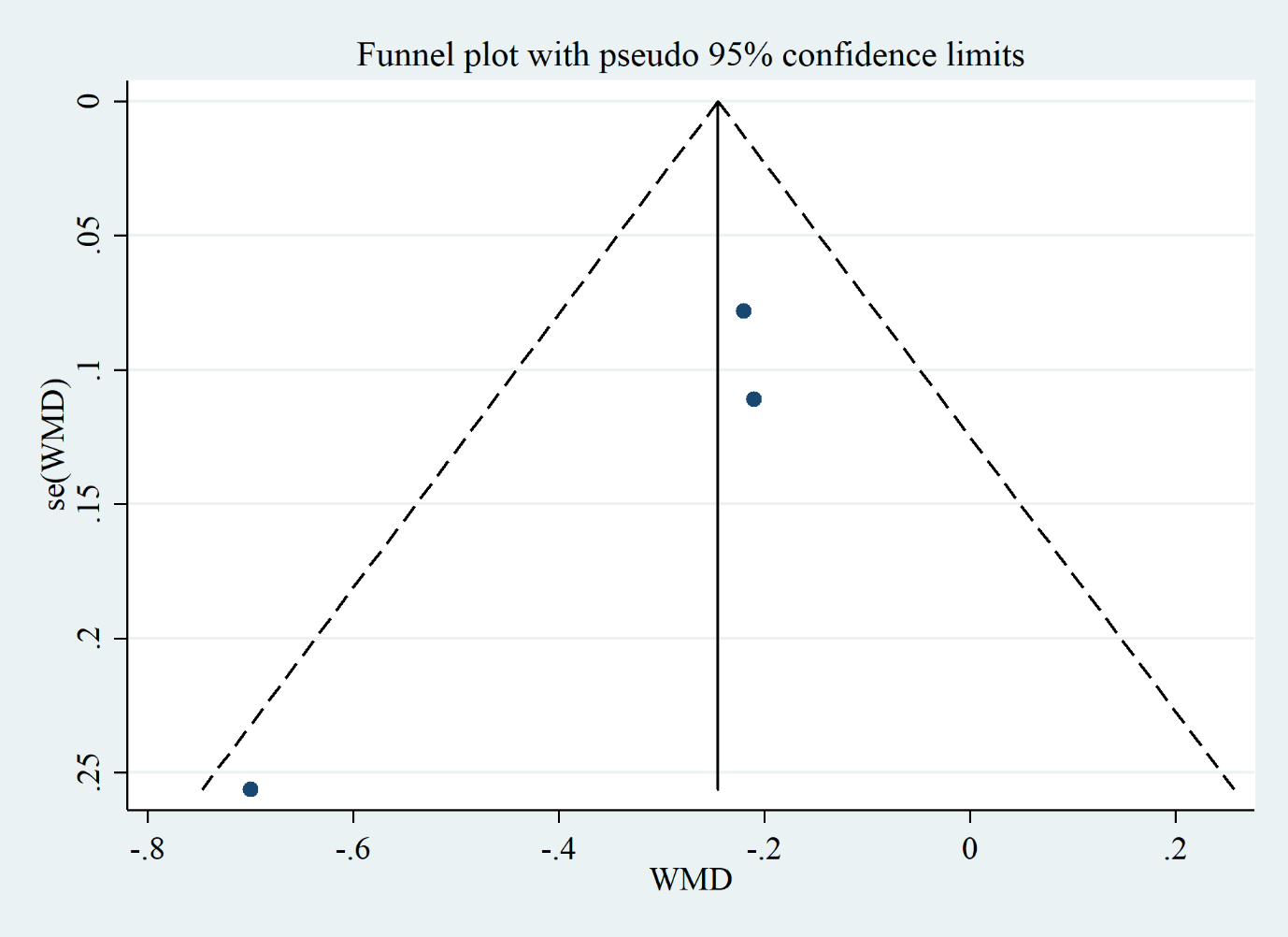
**

**Supplementary References**:

Ahmadi, S., Jamilian, M., Karamali, M., Tajabadi-Ebrahimi, M., Jafari, P., Taghizadeh, M., Memarzadeh, M.R., and Asemi, Z. (2017). Probiotic supplementation and the effects on weight loss, glycaemia and lipid profiles in women with polycystic ovary syndrome: a randomized, double-blind, placebo-controlled trial. *Human Fertility* 20**,** 254-261.

Cozzolino, M., Vitagliano, A., Pellegrini, L., Chiurazzi, M., Andriasani, A., Ambrosini, G., and Garrido, N. (2020). Therapy with probiotics and synbiotics for polycystic ovarian syndrome: a systematic review and meta-analysis. *Eur J Nutr* 59**,** 2841-2856.

Esmaeilinezhad, Z., Babajafari, S., Sohrabi, Z., Eskandari, M.-H., Amooee, S., and Barati-Boldaji, R. (2019). Effect of synbiotic pomegranate juice on glycemic, sex hormone profile and anthropometric indices in PCOS: A randomized, triple blind, controlled trial. *Nutrition, Metabolism and Cardiovascular Diseases* 29**,** 201-208.

Esmaeilinezhad, Z., Barati-Boldaji, R., Brett, N., De Zepetnek, J., Bellissimo, N., Babajafari, S., and Sohrabi, Z. (2020). The effect of synbiotics pomegranate juice on cardiovascular risk factors in PCOS patients: a randomized, triple-blinded, controlled trial. *Journal of endocrinological investigation* 43**,** 539-548.

Ge, L., Sadeghirad, B., Ball, G.D., Da Costa, B.R., Hitchcock, C.L., Svendrovski, A., Kiflen, R., Quadri, K., Kwon, H.Y., and Karamouzian, M. (2020). Comparison of dietary macronutrient patterns of 14 popular named dietary programmes for weight and cardiovascular risk factor reduction in adults: systematic review and network meta-analysis of randomised trials. *bmj* 369.

Ghanei, N., Rezaei, N., Amiri, G.A., Zayeri, F., Makki, G., and Nasseri, E. (2018). The probiotic supplementation reduced inflammation in polycystic ovary syndrome: a randomized, double-blind, placebo-controlled trial. *Journal of functional foods* 42**,** 306-311.

Goldenberg, J.Z., Day, A., Brinkworth, G.D., Sato, J., Yamada, S., Jönsson, T., Beardsley, J., Johnson, J.A., Thabane, L., and Johnston, B.C. (2021). Efficacy and safety of low and very low carbohydrate diets for type 2 diabetes remission: systematic review and meta-analysis of published and unpublished randomized trial data. *bmj* 372.

Hadi, A., Moradi, S., Ghavami, A., Khalesi, S., and Kafeshani, M. (2020). Effect of probiotics and synbiotics on selected anthropometric and biochemical measures in women with polycystic ovary syndrome: a systematic review and meta-analysis. *European Journal of Clinical Nutrition* 74**,** 543-547.

Heshmati, J., Farsi, F., Yosaee, S., Razavi, M., Rezaeinejad, M., Karimie, E., and Sepidarkish, M. (2019). The Effects of Probiotics or Synbiotics Supplementation in Women with Polycystic Ovarian Syndrome: a Systematic Review and Meta-Analysis of Randomized Clinical Trials. *Probiotics Antimicrob Proteins* 11**,** 1236-1247.

Jovanovski, E., Mazhar, N., Komishon, A., Khayyat, R., Li, D., Mejia, S.B., Khan, T., Jenkins, A.L., Smircic-Duvnjak, L., and Sievenpiper, J.L. (2020). Effect of viscous fiber supplementation on obesity indicators in individuals consuming calorie-restricted diets: a systematic review and meta-analysis of randomized controlled trials. *European journal of nutrition***,** 1-12.

Karamali, M., Eghbalpour, S., Rajabi, S., Jamilian, M., Bahmani, F., Tajabadi-Ebrahimi, M., Keneshlou, F., Mirhashemi, S.M., Chamani, M., and Gelougerdi, S.H. (2018). Effects of probiotic supplementation on hormonal profiles, biomarkers of inflammation and oxidative stress in women with polycystic ovary syndrome: a randomized, double-blind, placebo-controlled trial. *Archives of Iranian medicine* 21**,** 1-7.

Karimi, E., Heshmati, J., Shirzad, N., Vesali, S., Hosseinzadeh-Attar, M.J., Moini, A., and Sepidarkish, M. (2020). The effect of synbiotics supplementation on anthropometric indicators and lipid profiles in women with polycystic ovary syndrome: a randomized controlled trial. *Lipids in health and disease* 19**,** 1-9.

Kazemi, A., Soltani, S., Ghorabi, S., Keshtkar, A., Daneshzad, E., Nasri, F., and Mazloomi, S.M. (2020). Effect of probiotic and synbiotic supplementation on inflammatory markers in health and disease status: A systematic review and meta-analysis of clinical trials. *Clinical Nutrition* 39**,** 789-819.

Li, Y., Tan, Y., Xia, G., and Shuai, J. (2021). Effects of probiotics, prebiotics, and synbiotics on polycystic ovary syndrome: a systematic review and meta-analysis. *Critical Reviews in Food Science and Nutrition*.

Miao, C., Guo, Q., Fang, X., Chen, Y., Zhao, Y., and Zhang, Q. (2021). Effects of probiotic and synbiotic supplementation on insulin resistance in women with polycystic ovary syndrome: a meta-analysis. *J Int Med Res* 49**,** 3000605211031758.

Nasri, K., Jamilian, M., Rahmani, E., Bahmani, F., Tajabadi-Ebrahimi, M., and Asemi, Z. (2018). The effects of synbiotic supplementation on hormonal status, biomarkers of inflammation and oxidative stress in subjects with polycystic ovary syndrome: a randomized, double-blind, placebo-controlled trial. *BMC endocrine disorders* 18**,** 1-8.

Norman, G.R., Sloan, J.A., and Wyrwich, K.W. (2003). Interpretation of changes in health-related quality of life: the remarkable universality of half a standard deviation. *Medical care***,** 582-592.

Rashad, N.M., Amal, S., Amin, A.I., and Soliman, M.H. (2017). Effects of probiotics supplementation on macrophage migration inhibitory factor and clinical laboratory feature of polycystic ovary syndrome. *Journal of Functional Foods* 36**,** 317-324.

Revicki, D., Hays, R.D., Cella, D., and Sloan, J. (2008). Recommended methods for determining responsiveness and minimally important differences for patient-reported outcomes. *Journal of clinical epidemiology* 61**,** 102-109.

Samimi, M., Dadkhah, A., Kashani, H.H., Tajabadi-Ebrahimi, M., Hosseini, E.S., and Asemi, Z. (2019). The effects of synbiotic supplementation on metabolic status in women with polycystic ovary syndrome: a randomized double-blind clinical trial. *Probiotics and antimicrobial proteins* 11**,** 1355-1361.

Shamasbi, S.G., Dehgan, P., Charandabi, S.M.-A., Aliasgarzadeh, A., and Mirghafourvand, M. (2019). The effect of resistant dextrin as a prebiotic on metabolic parameters and androgen level in women with polycystic ovarian syndrome: a randomized, triple-blind, controlled, clinical trial. *European journal of nutrition* 58**,** 629-640.

Shamasbi, S.G., Dehghan, P., Charandabi, S.M.-A., Aliasgarzadeh, A., and Mirghafourvand, M. (2018). Effect of prebiotic on anthropometric indices in women with polycystic ovarian syndrome: a triple-blind, randomized, controlled clinical trial. *Iranian Red Crescent Medical Journal* 20.

Shamasbi, S.G., Ghanbari-Homayi, S., and Mirghafourvand, M. (2020). The effect of probiotics, prebiotics, and synbiotics on hormonal and inflammatory indices in women with polycystic ovary syndrome: a systematic review and meta-analysis. *European Journal of Nutrition* 59**,** 433-450.

Shoaei, T., Heidari-Beni, M., and Tehrani, H.G. (2015). Effects of probiotic supplementation on pancreatic β-cell function and c-reactive protein in women with polycystic ovary syndrome: a randomized double-blind placebo-controlled clinical trial. *International journal of preventive medicine* 6.

Tabrizi, R., Ostadmohammadi, V., Akbari, M., Lankarani, K.B., Vakili, S., Peymani, P., Karamali, M., Kolahdooz, F., and Asemi, Z. (2019). The Effects of Probiotic Supplementation on Clinical Symptom, Weight Loss, Glycemic Control, Lipid and Hormonal Profiles, Biomarkers of Inflammation, and Oxidative Stress in Women with Polycystic Ovary Syndrome: a Systematic Review and Meta-analysis of Randomized Controlled Trials. *Probiotics Antimicrob Proteins*.

Zhang, C., Sheng, Y., Jiang, J., Xue, Y., Yu, L., Tian, F., Zhao, J., Zhang, H., Jin, J., Zhai, Q.J.F.S., and Wellness, H. (2023). Probiotics supplementation for management of type II diabetes risk factors in adults with polycystic ovarian syndrome: a meta-analysis of randomized clinical trial. 12**,** 1053-1063.
